# Structural basis of DNA gyrase inhibition by antibacterial QPT-1, anticancer drug etoposide and moxifloxacin

**DOI:** 10.1038/ncomms10048

**Published:** 2015-12-07

**Authors:** Pan F. Chan, Velupillai Srikannathasan, Jianzhong Huang, Haifeng Cui, Andrew P. Fosberry, Minghua Gu, Michael M. Hann, Martin Hibbs, Paul Homes, Karen Ingraham, Jason Pizzollo, Carol Shen, Anthony J. Shillings, Claus E. Spitzfaden, Robert Tanner, Andrew J. Theobald, Robert A. Stavenger, Benjamin D. Bax, Michael N. Gwynn

**Affiliations:** 1 Antibacterial Discovery Performance Unit, Infectious Diseases, Therapy Area Unit, GlaxoSmithKline, 1250 South Collegeville Road, Collegeville, Pennsylvania 19426-0989, USA; 2 Platform Technology and Science, GlaxoSmithKline, Medicines Research Centre, Gunnels Wood Road, Stevenage, Hertfordshire SG1 2NY, UK

## Abstract

New antibacterials are needed to tackle antibiotic-resistant bacteria. Type IIA topoisomerases (topo2As), the targets of fluoroquinolones, regulate DNA topology by creating transient double-strand DNA breaks. Here we report the first co-crystal structures of the antibacterial QPT-1 and the anticancer drug etoposide with *Staphylococcus aureus* DNA gyrase, showing binding at the same sites in the cleaved DNA as the fluoroquinolone moxifloxacin. Unlike moxifloxacin, QPT-1 and etoposide interact with conserved GyrB TOPRIM residues rationalizing why QPT-1 can overcome fluoroquinolone resistance. Our data show etoposide's antibacterial activity is due to DNA gyrase inhibition and suggests other anticancer agents act similarly. Analysis of multiple DNA gyrase co-crystal structures, including asymmetric cleavage complexes, led to a ‘pair of swing-doors' hypothesis in which the movement of one DNA segment regulates cleavage and religation of the second DNA duplex. This mechanism can explain QPT-1's bacterial specificity. Structure-based strategies for developing topo2A antibacterials are suggested.

## Abstract (web summary)

Type IIA topoisomerases (topo2As) create transient double-strand DNA breaks. Here, the authors report structures showing how QPT-1 binds in the DNA/topo2A complex at the same site as the fluoroquinolone moxifloxacin, and discuss the potential for developing new classes of antibiotics.

## Graphical abstract

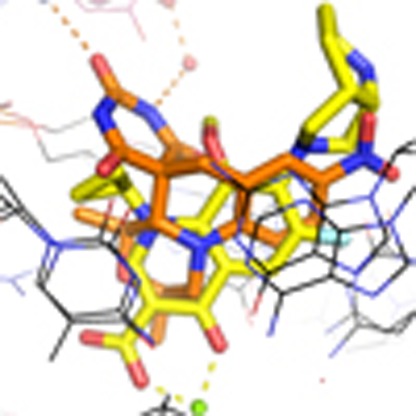

Fluoroquinolone antibacterials and several classes of anti-cancer agents function by generation of ‘poison' complexes between type IIA topoisomerases (topo2As) and DNA^1^,^2^,^3^,^4^,^5^. Structures of such ‘poison' complexes such as the fluoroquinolone moxifloxacin with DNA and a bacterial topo2A^6^, and of the anticancer drug etoposide with DNA and human Top2β^7^, showed the drugs bound in the DNA at the cleavage sites making specific interactions with the proteins and inhibiting DNA religation (Fig. 1). Two new classes of antibacterials that target the DNA gate of bacterial topo2As are currently in phase II clinical trials: gepotidacin (pronounced Jepo- tide- a- cin, formerly GSK2140944 (ref. 8)), a novel bacterial topoisomerase inhibitor (NBTI) related to GSK299423 (ref. 9), and AZD0914 (refs 10, 11, 12, 13), a derivative of QPT-1 (quinoline pyrimidine trione-1)^14^ (Supplementary Fig. 1).

Bacteria have two well-conserved topo2As, DNA gyrase and topoisomerase IV (topo IV), which allow for dual targeting by antibacterials, affording reduced spontaneous resistance^15^. DNA gyrase consists of two subunits, GyrA and GyrB, and functions as an A_2_B_2_ tetramer, as does topo IV, which consists of ParC and ParE subunits. Topo2As regulate DNA topology^16^ by creating a four base-pair-staggered double-stranded break (DSB) in one DNA duplex, passing another DNA duplex through this break and then resealing the break (Fig. 1). The two catalytic gates of the enzyme, the amino-terminal ATP gate and the central DNA-cleavage gate, are each targeted by multiple antibacterial agents^5^. No inhibitors of the ATPase domain (ATP gate) are currently in clinical use; in contrast, the highly successful quinolone/fluoroquinolone antibacterials^1^ have been in clinical use for nearly 50 years, with new members of this class currently in development^5^. The primary interaction of fluoroquinolones with the protein is via a water–metal ion bridge^6^,^17^,^18^ to two conserved residues on GyrA (Ser84 and Glu88 in *S. aureus* GyrA); these two residues are the most commonly mutated in clinical isolates resistant to fluoroquinolones^1^,^19^. Residues equivalent to Ser84 and Glu88 are conserved in bacterial topo2As, but the corresponding residues in mammalian topo2As are different, accounting for much of the specificity of fluoroquinolones^1^,^17^. Eukaryotic topo2As (such as human Top2α and Top2β, and yeast Topo II) function as homodimers, with regions equivalent to GyrB and GyrA encoded at the N- and carboxy-terminal ends, respectively, of a single polypeptide. Human topo2As are the targets of several anticancer agents^2^ including doxorubicin, amascrine, mitoxantrone^20^ and etoposide, with the latter in clinical use for over 30 years^7^. A high-resolution structure of etoposide with hTop2β^7^ showed the drug bound in the cleaved DNA making interactions with Gln778 and Met782.

QPT-1 (Fig. 1) represents a novel class of antibacterial compounds targeting bacterial DNA gyrase and topo IV, with a broad spectrum of antimicrobial activity and good selectivity with respect to human Top2α^14^,^21^. The class is reported to overcome target-mediated fluoroquinolone resistance and this has attracted significant industry effort, with several hundred analogues reported in the scientific and patent literature from multiple companies^5^. However, the structural and mechanistic basis of action of this class and the mechanism by which it overcomes fluoroquinolone cross-resistance have not previously been reported.

In this study we show that etoposide and other anticancer agents also have a surprising level of activity against a range of bacteria, and that this is due to inhibition of DNA gyrase. We also report the first co-crystal structures of DNA-cleavage complexes for QPT-1, as well as co-crystal structures for etoposide and moxifloxacin. Our structures include the first asymmetric topo2A DNA-cleavage complexes, showing that inhibitors can stabilize an asymmetric conformation of the DNA gate. Out of the diversity of inhibitors discussed, important generalizable themes and insights emerge for overcoming fluoroquinolone resistance using new chemistries and binding strategies, including targeting the GyrB TOPRIM domain.

## Results

### Structures of *S. aureus* gyrase DNA-cleavage complexes

**Figure 1:**
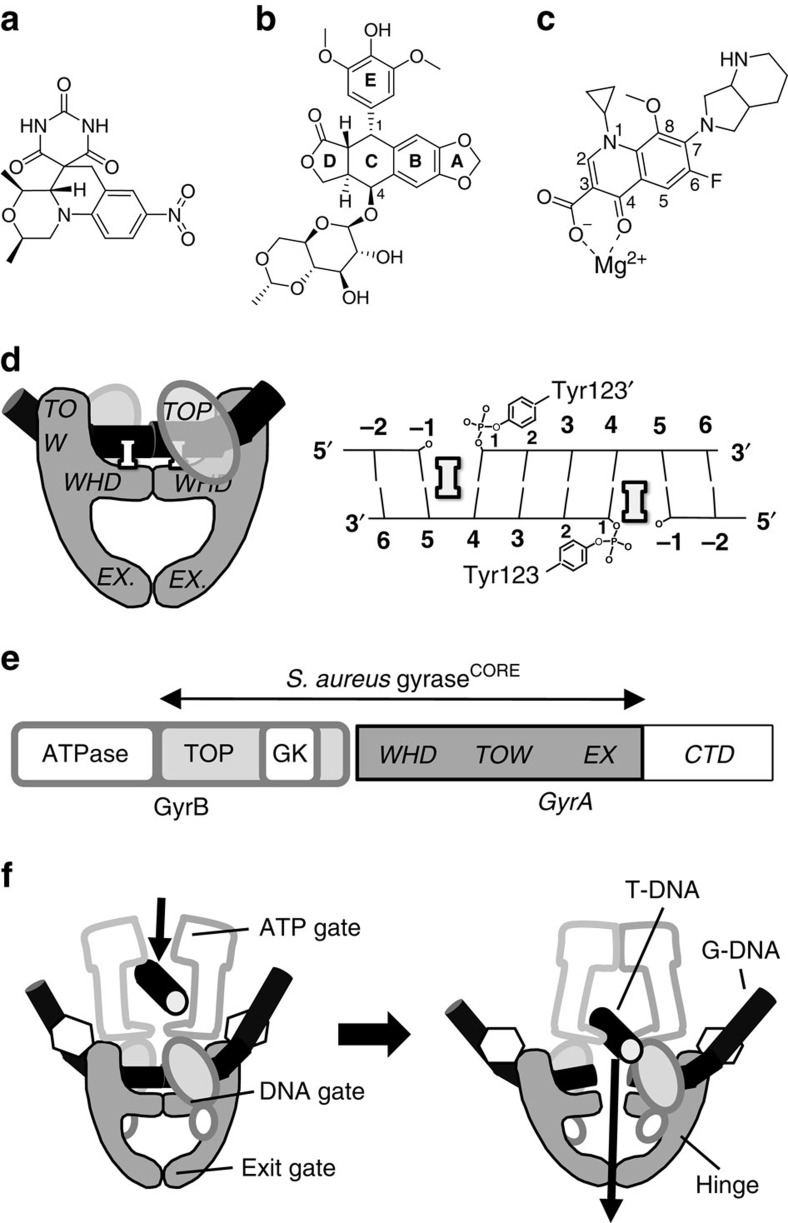
Schematics of *S. aureus* DNA gyrase cleavage complexes with inhibitors. Chemical structures of (**a**) QPT-1, (**b**) etoposide and (**c**) moxifloxacin (with associated magnesium ion). (**d**) Schematic of structure of *S. aureus* gyrase^CORE^ DNA-cleavage complex containing two inhibitors (**I**) binding in the cleaved DNA-blocking re-ligation. By convention, nucleotides are numbered relative to the cleavage sites. (**e**) DNA gyrase consists of two subunits: GyrB and GyrA. The *S. aureus* gyrase^CORE^ construct used to determine crystal structures reported in this study is a fusion of the C-terminal TOPRIM (TOP) domain of GyrB with the N-terminal winged helical domain (WHD), tower (TOW) and exit gate (EX) domains from GyrA. The small Greek Key (GK) domain has been deleted^9^. (**f**) Schematic of DNA gyrase showing how the gate DNA (G-DNA) is cleaved to allow passage of the transport DNA (T-DNA), to regulate DNA topology.

**Figure 2:**
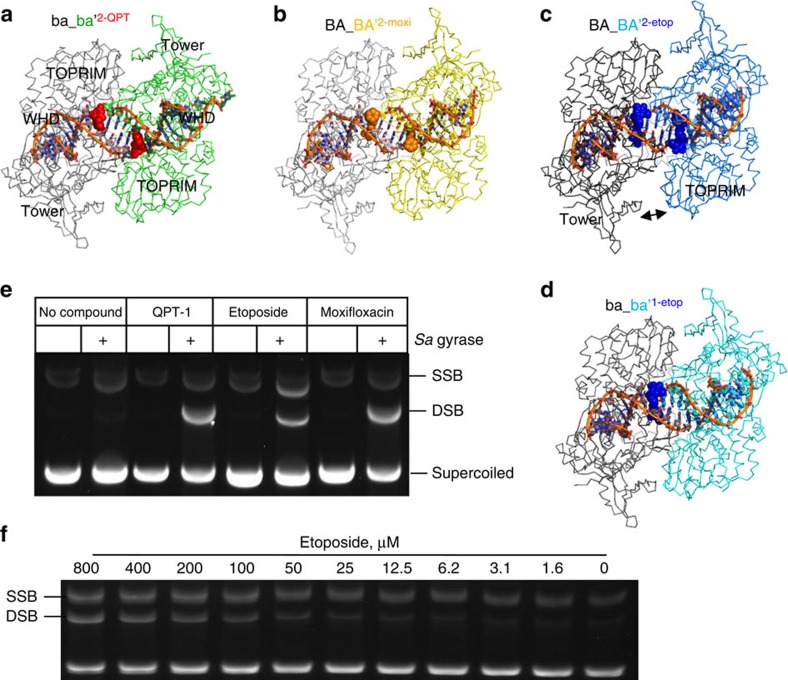
*S. aureus* gyrase DNA-cleavage activity and crystal structures with three different inhibitors. (**a**) The structure of the 2.5 Å ba_ba'^2−QPT^ complex viewed down the twofold axis. QPT-1 (red space fill) with gyrase^core^ (grey/green Cα trace) and cleaved DNA (backbone trace, orange; sticks, carbons grey/green; oxygens, red; nitrogens, blue; phosphorus, orange) with two QPT-1 molecules bound. The three structural domains that make up the DNA gate are labelled. It is noteworthy that the WHD (winged helical domain) is underneath the DNA in this view. (**b**) BA_BA'^2–moxi^ complex of moxifloxacin (2.95 Å; orange space fill) with gyrase^core^ and DNA with two moxifloxacin molecules bound. (**c**) BA_BA'^2–etop^ complex of etoposide (2.8 Å; blue space fill) with gyrase^core^ and DNA with two etoposide molecules bound. It is noteworthy that this complex is asymmetric, with the labelled Tower and TOPRIM domains further apart than in other complexes shown (double arrow). (**d**) ba_ba'^1–etop^ complex of etoposide (2.45 Å; blue space fill) with gyrase^core^ and doubly nicked DNA, which has only one etoposide bound. Apart from the compound, the complex is C2 symmetric. (**e**) DNA-cleavage assay showing moxifloxacin and QPT-1 stabilize DSB with *S. aureus* DNA gyrase, whereas etoposide stabilizes both SSB and DSB. Each compound (100 μM) was incubated with supercoiled pBR322 and full-length *S. aureus* gyrase as described in Methods. (**f**) The etoposide-stabilized SSB and DSB persist over a wide range of etoposide concentrations with *S. aureus* DNA gyrase.

**Figure 3:**
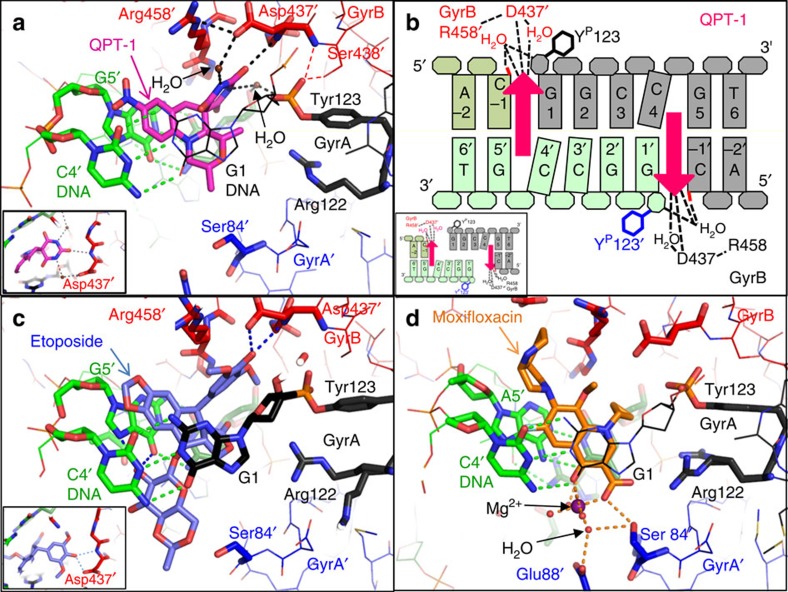
Binding sites for QPT-1, etoposide and moxifloxacin in *S. aureus* DNA gyrase complexes. (**a**) QPT-1 structure (2.5 Å) with *S. aureus* gyrase^CORE^. The central oxygen of the barbituric acid moiety of QPT-1 (pink carbons) interacts with the main-chain N–H of Asp437 of GyrB (dotted black lines). Water molecules (small red spheres) mediate interactions from the barbituric acid nitrogens of QPT-1 to the protein. DNA shown in green or black carbons. The second GyrB/A subunits are labelled as prime. A different view of the Asp437 interaction of GyrB with barbituric acid of QPT-1 is shown in the inner panel. (**b**) Schematic showing how QPT-1 binds in the cleaved DNA and interacts with GyrB via its barbituric acid moiety (arrowhead). The insert panel shows the DNA gate opening. (**c**) Etoposide structure (2.8 Å) with *S. aureus* gyrase^CORE^. It is noteworthy that the central oxygen of the dimethoxy phenol group of etoposide (blue carbons) interacts with both the side-chain and main-chain N–H of Asp437 of GyrB. A different view of the Asp437 interaction with the dimethoxy phenol of etoposide is shown in the inner panel. (**d**) Moxifloxacin structure (2.95 Å) with *S. aureus* gyrase^CORE^. Moxifloxacin (orange carbons) interacts with Ser84 and Glu88 of GyrA via the magnesium–water ion bridge (dotted orange lines). In all structures shown, a phosphotyrosine linkage exists between Tyr123 of GyrA and DNA (G1 nucleotide is shown as black, light sticks so that compound is not hidden).

As part of a structure-based approach to develop novel bacterial topo2A pharmacophores, we co-crystallized and determined structures^22^ of DNA-cleavage complexes of the *S. aureus* B27-A56(GKdel) fusion truncate (gyrase^CORE^) with QPT-1, etoposide and moxifloxacin (Figs 1, 2, 3, Table 1, Supplementary Fig. 2 and Supplementary Table 1). In DNA-cleavage assays, QPT-1 and moxifloxacin stabilized DSBs with *S. aureus* DNA gyrase, whereas etoposide stabilized both single-stranded breaks (SSB) and DSBs (Fig. 2e,f and Table 2). Compound-stabilized DNA-cleavage activities of the *S. aureus* gyrase^CORE^ protein were comparable to that of the wild-type protein (Supplementary Figs 3 and 4), indicating the gyrase^CORE^ truncate used in crystal studies was functionally active.

All DNA-cleavage complex structures solved with QPT-1 and moxifloxacin contain two inhibitor molecules bound (Fig. 2 for structures of complexes). With etoposide and moxifloxacin, we solved 2.80 and 2.95 Å DNA-cleavage complex structures in a P2_1_ unit cell with two complexes in the asymmetric unit (Table 1). With QPT-1, in addition to solving a 3.15-Å structure in this P2_1_ cell, we also solved a 2.50-Å QPT-1 structure in a P6_1_ cell with one complex in the asymmetric unit; the three QPT-1 complexes were similar but not identical (see bottom line of Table 1 (for nomenclature) and below). In all the cleavage complexes reported here, there was clear density for compounds at both cleavage sites (Supplementary Fig. 2) and for the phosphotyrosine bond formed between Tyr123/Tyr123' of GyrA and the DNA (residues with a ‘ suffix are from the second subunit in the dimer).

In addition to the *S. aureus* cleavage complexes described, we also solved a 2.45-Å structure with a single etoposide bound (Fig. 2d and Supplementary Fig. 2k). The DNA used for this structure was doubly nicked, with artificial breaks in the DNA backbone at both cleavage sites. We further solved a 2.65-Å binary complex (Table 1) with this DNA (without compound). The basic binding modes for the three inhibitors are shown in Fig. 3. Small variations in the binding modes for the same compound in different complexes were observed (see below). In the 2.95-Å *S. aureus* moxifloxacin structure (Fig. 3d) the critical interactions via the water–ion bridge^18^ with GyrA Ser84 and Glu88 are similar to those we described with a Gram-negative topo IV^6^. The binding mode for etoposide in *S. aureus* (Fig. 3c) is similar to that observed in the 2.1 Å human topo2β complex^7^, which has two etoposide molecules bound.

### QPT-1 binding is at the same site as moxifloxacin

**Figure 4:**
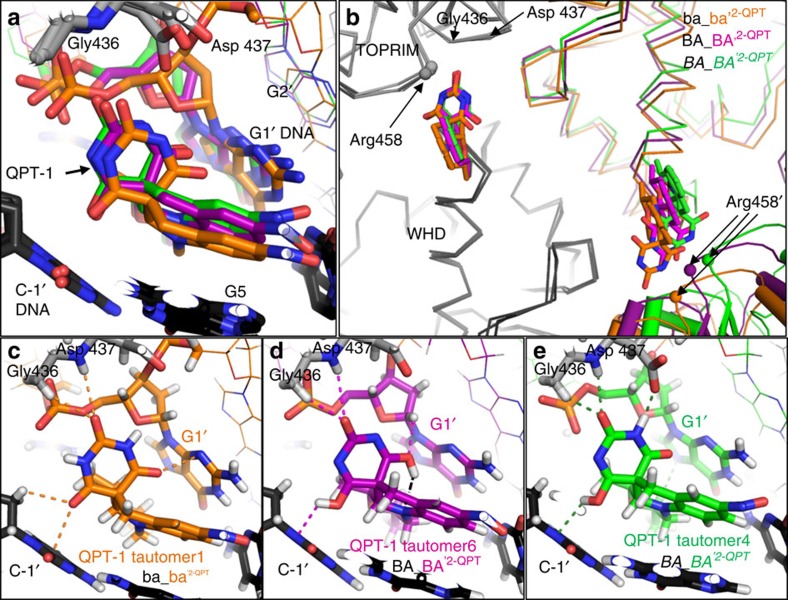
Comparison of QPT-1-binding sites. (**a**) Close-up of QPT-1-binding site from ba_ba'^2–QPT^, BA_BA'^2–QPT^ and *BA_BA'*^2–QPT^ complexes superposed as in **b**. The differences in the relative positions of the G1′ nucleotides are worth noting. Hydrogens are not shown. (**b**) The three QPT-1 cleaved complexes ba_ba'^2–QPT^, BA_BA'^2–QPT^ and *BA_BA'*^*2–QPT*^, each with two QPT-1 molecules bound, were superposed using Cα atoms from both the TOPRIM and WHD domains of the ba (BA, *BA*) subunits (grey). Protein subunits are shown as Cα traces, except for the TOPRIM domain shown in coloured cartoon representation (bottom right). For clarity, the DNA is not shown. Relative shifts of up to 4.1 Å are observed in the relative position of Arg 458' (small coloured spheres, bottom right). (**c**–**e**) Close-ups of each of the QPT-1-binding sites shown in **a**, but showing hydrogen atoms (white). The barbituric acid moiety has been modelled in three different tautomeric forms in the three binding sites. Dotted lines represent interactions between tautomers of QPT-1 and GyrB or DNA. It is worth noting that an internal H-bond is shown for tautomer 6 in **d**.

The co-crystal structures with QPT-1 are novel and demonstrate for the first time that the functional inhibition of topo2As by QPT-1 is similar to that of fluoroquinolones, despite a very different chemical structure, supporting the biochemical data in which QPT-1 induces double-strand cleavage of DNA mediated by DNA gyrase (Fig. 2a,e and Supplementary Fig. 2). Similar to the fluoroquinolones, QPT-1 is bound between the +1 and −1 bases at the DNA cleavage site inhibiting DNA re-ligation. This establishes that the clinically validated inhibition mechanism of the fluoroquinolones is achievable with very different interactions from a completely different chemotype (Fig. 3a,d). QPT-1 is bound in overlapping space with the fluoroquinolones but the binding mode is strikingly different (Fig. 3). QPT-1 interacts with residues in the TOPRIM domain of GyrB, with the barbituric acid moiety of QPT-1 (Supplementary Fig. 1) packing under Arg458 and making a hydrogen bond to the main-chain N–H of Asp437 (Fig. 3a), and quite a close interaction with the main-chain CH_2_ of Gly436 (Fig. 4). QPT-1 also makes an indirect hydrogen bond via a water molecule to the side chain of Asp437. The interaction of QPT-1 with Asp437 somewhat resembles that made by the central hydroxyl of etoposide (Fig. 3a,c inserts and Supplementary Fig. 2). Etoposide-resistant strains of *S. aureus* with a D437N or D437A mutation (see below and Table 2b) are also resistant to QPT-1. Arg458 and Asp437 are from two sequence motifs, PLRGK and EGDSA, which are conserved in topo2As from bacteria to man (Supplementary Fig. 5) and a similar interaction is observed in the etoposide-human Top2β complex^7^. This sequence conservation may explain the cross-activity of etoposide between bacteria and man but does not readily explain the high bacterial selectivity of QPT-1 against human Top2α (Table 2 and Supplementary Fig. 3).

The three different QPT-1 cleavage complexes have the DNA gate closed in different ways (Fig. 4). These complexes (ba_ba'^2–QPT^, BA_BA'^2–QPT^ and *BA_BA'*^*2*−*QPT*^; see Table 1) come from the 2.5- and 3.15-Å QPT-1 co-crystal structures, which were obtained under the same crystallization conditions^22^. When the QPT-1-binding sites are compared (Fig. 4a and Supplementary Fig. 6), the largest difference is seen in the relative position of the G1 nucleotide, which is covalently attached to the catalytic tyrosine from the opposing subunit (Fig. 3b). One possible explanation for this spatial diversity may be the range of tautomeric forms that the barbituric acid moiety of QPT-1 can adopt as the DNA gate moves at the dimer interface (Fig. 4, Supplementary Fig. 6 and Supplementary Table 2). Barbituric acid^23^ and its derivatives^24^ are known to crystallize in several different keto or enol tautomeric forms, stabilized by hydrogen bonding in different crystal lattices^24^. In docking experiments, eight tautomers of QPT-1 were evaluated in the two binding sites of each QPT-1 complex and tautomers were tentatively assigned to the different QPT-1-binding sites as described in Methods. As the central keto oxygen of the barbituric acid moiety of QPT-1 points between the NH of Asp437 and the CH_2_ of Gly436 of GyrB, this oxygen is unlikely to be protonated in any of the QPT-1-binding sites. In contrast, the other two oxygens on the barbituric acid were modelled as either protonated enol groups (tautomers 6 and 4 in Fig. 4d,e) or unprotonated keto groups (tautomers 1 and 4 in Fig. 4c,e). These two oxygens point towards the *π*-electrons of the G+1 and C−1 nucleotide bases^25^. The tertiary ‘anilino' nitrogen atom in the central ring of QPT-1 can also adopt different forms. In our structures, it is modelled in a near-planar *sp*^2^ configuration in five of the six QPT-1-binding sites, but in the sixth is modelled in a more tetrahedral *sp*^3^ configuration, accepting an internal hydrogen bond from a neighbouring OH group (tautomer 6 in Fig. 4d and Supplementary Table 2). The conformational flexibility of QPT-1, and its ability to adopt different tautomeric states, may allow QPT-1 to maintain favourable interactions with its binding site as the two subunits at the DNA gate move relatively to each other and the size and shape of the QPT-1-binding pocket changes.

**Figure 5:**
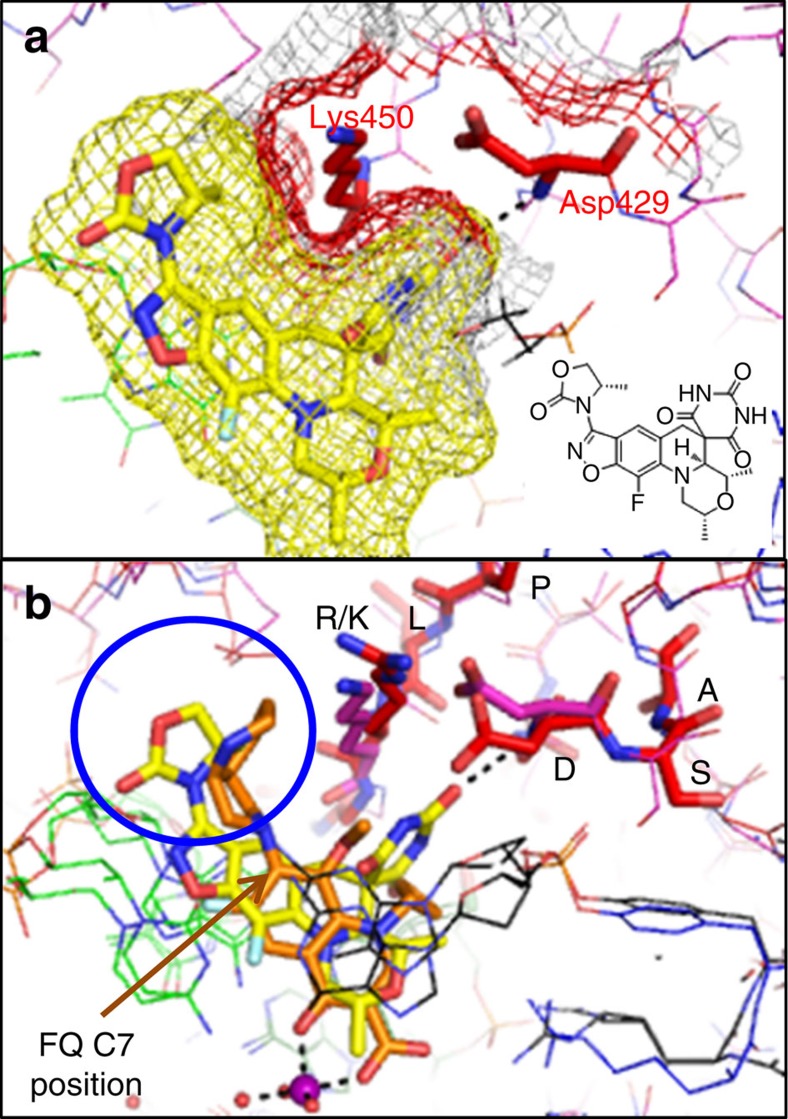
A model of AZD0914 in *N. gonorrhoeae* DNA gyrase. (**a**) AZD0914 modelled into a structure of *N. gonorrhoeae* DNA gyrase (see Methods for details). Mutations of Lys450 and Asp429 have been found to give low levels of resistance in laboratory-generated resistant bacteria^12^. A mesh molecular surface around the compound (yellow) and Lys450 and Asp429 (red) are shown. (**b**) The model of AZD0914 in *N. gonorrhoeae* DNA gyrase is shown superposed on the crystal structure of moxifloxacin in *S. aureus* DNA gyrase. It is noteworthy that Lys450 (equivalent to Arg458 in *S. aureus*) and Asp429 (Asp437 in *S. aureus*) are from two conserved sequence motifs: PL**R**/**K**GK and EG**D**SA. The blue circle highlights that the region occupied by the substituent from the C7 position of the fluoroquinolone (FQ) is also occupied by a substituent in AZD0914.

AZD0914 is a compound related to QPT-1 (refs 11, 26, 27 and Supplementary Fig. 1) that is currently in a phase II clinical trial for the treatment of *Neisseria gonorrhoeae*^10^,^12^,^13^. We built a model (see Methods) of AZD0914 in a DNA-cleavage complex with *N. gonorrhoeae* DNA gyrase, based on the 2.5-Å QPT-1 crystal structure with *S. aureus* gyrase. Two residues, GyrB Asp429 and Lys450, which pack against AZD0914 in our model (Fig. 5a), have been observed to give low-level resistance when mutated in *N. gonorrhoeae* DNA gyrase^12^. It is interesting that the benzisoxazole group of AZD0914 has a vector pointing a pendant oxazole ring into a similar place as the C7 substituent from moxifloxacin (Fig. 5b), suggesting that structure–activity relationships from the highly variable C7 position of fluoroquinolones could be used to develop new analogues of QPT-1.

### Etoposide is an antibacterial via inhibition of DNA gyrase

Etoposide's activity against eukaryotic topo2As has been extensively studied^2^,^28^,^29^ including a structure in complex with human Top2β^7^. However, there are few reports on its inhibition of bacterial enzymes or on its antibacterial activity^30^,^31^ and activity against wild-type bacteria had not been demonstrated to be due to topoisomerase inhibition. We found that etoposide has activity against both *S. aureus* and *Escherichia coli* topo2As, through the stabilization of DNA-cleavage complexes, and that etoposide has dual-targeting activities for gyrase and topo IV (Table 2, Supplementary Fig. 4 and Supplementary Table 3). In our co-crystal structures of etoposide with *S. aureus* gyrase, the central hydroxyl of the dimethoxy phenol group of etoposide (ring E in Fig. 1b) interacts with the same main-chain N–H of Asp437 of GyrB (Fig. 3c) as QPT-1. Hence, etoposide and QPT-1, despite having very different chemotypes, are nearly isosteric, both interacting with GyrB Asp437 (Fig. 3a,c small insert panels). The potency of etoposide for DNA cleavage with *S. aureus* gyrase was weaker than that for human Top2α (Table 2), possibly due to the interaction of the glycosidic moiety of etoposide with Gln778 and Met782 in the human Top2β structure^7^. In contrast, in the *S. aureus* etoposide structures the equivalent residues, GyrA Ser84 and Glu88, do not make specific interactions with etoposide (Fig. 3c). However, mutation of the residue equivalent to Ser84 in *E. coli* DNA gyrase can enhance the activity of etoposide against the bacterial enzymes^31^,^32^, showing that a different amino acid at position 84 of the GyrA subunit of DNA gyrase can make a favourable interaction with etoposide.

An *in vitro* gyrase-dependent DNA replication assay^9^,^33^ using toluenized cells from *E. coli* gave an half-maximal inhibitory concentration (IC_50_) of 4.3 μM, showing that inhibition of bacterial topo2A with etoposide results in functional inhibition of bacterial DNA replication (Table 2). Etoposide demonstrates potent cytotoxicity to mammalian cells despite modest topo2A inhibitory action, likely to be due to the generation of highly lethal ‘poison' complexes between the enzyme and chromosomal DNA. Likewise, relatively weak inhibition of bacterial DNA gyrase results in significant *in vitro* DNA replication inhibition and whole-cell antibacterial activity (Table 2). Etoposide showed antibacterial activity against a range of Gram-positive pathogens and, similar to QPT-1, against efflux-deficient mutants of Gram-negative pathogens (Table 2 and Supplementary Table 4). Among Gram-positive pathogens, the most notable activity was against *Streptococcus pneumoniae*, with antibacterial activity retained against isogenic mutants harbouring mutations in GyrA/ParC that confer fluoroquinolone resistance (Supplementary Table 5). Whereas wild-type Gram-negative pathogens were generally insensitive to etoposide, their isogenic efflux-deficient mutants were highly sensitive, consistent with increased efflux leading to reduced access of etoposide to the intracellular target (Supplementary Table 4).

To genetically demonstrate that the antibacterial activity of etoposide was due to DNA gyrase inhibition, spontaneous mutants of *S. pneumoniae* and *S. aureus* resistant to etoposide were selected in the laboratory. DNA sequencing of gyrase and topo IV genes identified mutations in DNA gyrase, including the GyrB TOPRIM domain, in resistant mutants from both species (Table 2 and Supplementary Table 6). To prove that gyrase mutation was solely responsible for the observed etoposide resistance, two of the mutations (GyrB Δ L407–S410 and GyrB R447C) obtained in *S. pneumoniae* 1629 were transformed into *S. pneumoniae* R6. The resultant transformants contained the same mutation and were similarly resistant to etoposide as the original spontaneous mutant. This demonstrates for the first time that etoposide has an antibacterial activity against gyrase wild-type bacteria that is directly attributable to DNA gyrase inhibition.

The *S. aureus* and *S. pneumoniae* etoposide*-*resistant mutants were tested for their susceptibility to a selected range of other antibacterial and anticancer topo2A inhibitors (Table 3 and Supplementary Table 6) chosen for structural diversity and known structural basis of action against human topo2As^2^,^20^ (Supplementary Fig.1). The antibacterial spectrum of teniposide, a podophyllotoxin related to etoposide, was similar to that of etoposide against wild-type strains (Supplementary Table 4) and was similarly affected by etoposide-resistant mutants (Table 3 and Supplementary Table 6). Notable hypersensitivity in certain etoposide-resistant mutants were observed for mitoxantrone, amsacrine, daunorubicin, doxorubicin and ellipticine (Table 3 and Supplementary Table 6), suggesting that the antibacterial activity of these human topo2A inhibitors like etoposide, are due to gyrase inhibition, but that they do not inhibit the same conformational state of the enzyme^20^. The complex variety of drug resistance and hypersensitivity phenotypes for anticancer agents observed in the gyrase mutants has also been observed in eukaryotic topo2A mutants^34^,^35^ and mapping the positions of the resistant mutants onto structures shows that, although some are footprint mutations^7^, others are distant from the etoposide-binding sites.

### Structural analysis reveals asymmetric cleavage complexes

**Figure 6:**
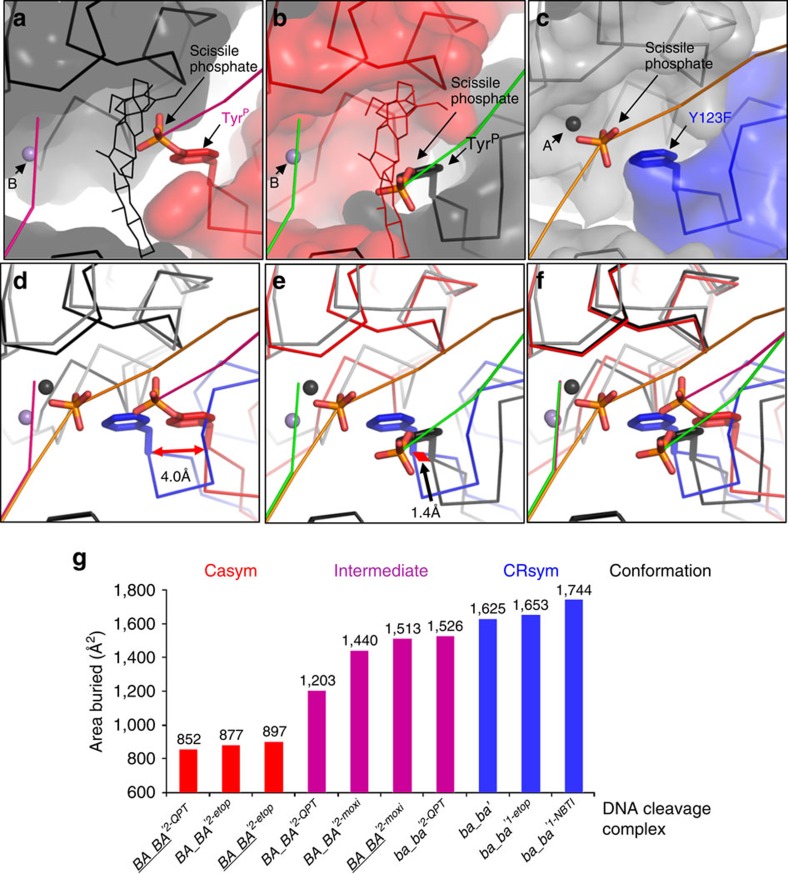
Comparison of active sites in asymmetric (Casym) and symmetric (CRsym) *S. aureus* DNA gyrase complexes. (**a**,**b**) Views of the two active sites in the Casym *BA_BA'*^*2–etop*^ etoposide complex (the complex is asymmetric; hence, the two active site are different; this common asymmetric conformation (Casym) is also seen in the BA_BA'^*2–etop*^ and *BA_BA'*^2–*QPT*^ complexes). The cleaved DNA backbone is shown as a pink (**a**) or green (**b**) line. Surfaces (red and black) are shown on the two protein subunits (catalytic Tyr^P^123 surface not shown for clarity). Etoposides are shown as thin black (**a**) or red (**b**) lines. The scissile phosphates are shown in red and orange sticks forming phosphotyrosine linkages with Tyr^P^123 of GyrA. A metal ion (small purple sphere) is present at the non-catalytic B site. (**c**) View of an active site in the CRsym ba_ba'^1–NBTI^ complex^9^ (the complex is C2 symmetric; hence, the other active site is the same). The catalytic tyrosine has been mutated to a phenylalanine; thus, the DNA (orange line) is not cleaved. The position of the scissile phosphate is shown in stick representation for orientation. A metal ion (small black sphere) is present at the catalytic A site. (**d**) Comparison of **a** and **c** shows a relative shift of 4.0 Å between the Cα positions of Tyr^P^/Phe 123. Protein surfaces and etoposide have been removed for clarity. (**e**) Comparison of **b** and **c** shows a relative shift of 1.4 Å between the Cα positions of Tyr^P^/Phe 123. (**f**) Comparison of **a**,**b** and **c**. (**g**) Area buried (Å^2^) between the two protein subunits at the DNA gate in nine *S. aureus* B27A56Gkdel complexes with doubly cleaved or doubly nicked DNA (this study) and one *S. aureus* B27A56Gkdel complex with uncleaved DNA (ba_ba'^1–NBTI^)^9^. Second complexes shown in italics in Table 1 are underlined here for clarity.

To analyse the different configurations observed at the DNA gate, 12 of our complexes of *S. aureus* DNA gyrase with DNA and 4 unrelated antibacterial compounds, were superposed (see Methods, Supplementary Figs 7 and 8, and Supplementary Tables 7 and 8 for details). A comprehensive comparative analysis revealed a range of conformations with two main clusters containing five and three structures, respectively, at either end of the range (Fig. 6g and Supplementary Fig. 7). We named the conformation represented by the first cluster of five structures, CRsym (for cleavage and/or re-ligation-competent symmetrical conformation, blue structures in Supplementary Fig. 7). These CRsym structures are ∼C2 symmetric and include a structure with the metal ion at the A (or 3′)-site^9^ (Fig. 6c). The presence of a metal ion (Mg^2+^) at the A (or 3′)-site is believed to be necessary for topo2As, to both cleave and re-ligate the DNA^9^,^36^,^37^ (see Supplementary Discussion). The catalytic CRsym-type conformation appears to be conserved between eukaryotic and prokaryotic topo2As (Supplementary Fig. 8). All the CRsym complexes have either one or no compound bound and have a larger area buried between the two subunits at the DNA gate (1,625–1,744 Å^2^) than is observed in the other seven complexes (Fig. 6g and Supplementary Fig. 7).

The second cluster consists of three asymmetric complexes, each of which has two compounds bound. We termed this cluster Casym (for common asymmetrical conformation, red structures in Supplementary Fig. 7). All three Casym structures (both 2.8 Å etoposide complexes with two compounds bound and one of the 3.15 Å QPT-1 complexes) have angles between two subunits at DNA gate of 173.5±0.6° (compared with 179.4°–179.9° for CRsym structures) indicative of a ‘half-open' conformation at the DNA gate (Supplementary Table 8). The Casym complexes have the smallest areas buried (852–897 Å^2^) between the two subunits at the DNA gate (Fig. 6g and Supplementary Figs 7 and 9). In a Casym complex, one active site is ‘open' (Fig. 6a) with relatively little contact between the two protein subunits, whereas the other active site (Fig. 6b) is closed and is quite similar to the configuration seen in a CRsym active conformation (Fig. 6e,f). Relative to a CRsym structure, the catalytic Tyr^P^ in the Casym etoposide complexes has shifted by 4 Å on the ‘open' half and only 1.4 Å on the closed half (Fig. 6d–f). To our knowledge, these are the first asymmetric topo2A DNA-cleavage complexes reported and show that inhibitors can stabilize an asymmetric conformation of the DNA gate. The area buried between the two subunits in the hTOP2β etoposide complex is also small (565 Å^2^)^7^ but the DNA gate has cracked open in a different way in the human etoposide complex (Supplementary Fig. 10e).

The four cleavage complexes (two QPT-1 and two moxifloxacin complexes) not in the CRsym or Casym clusters have a range of configurations (angles between two subunits at DNA gate of 177.5–178.9°) intermediate between those of the two clusters. Furthermore, these intermediate complexes have buried areas between those observed for the CRsym and Casym clusters (1,203–1,526 Å^2^, purple structures in Supplementary Fig. 7). The area buried at a protein:protein interface is sometimes used as a surrogate for stability^38^, suggesting that in the absence of compound the Casym half-open conformation would be the least stable of the *S. aureus* DNA gyrase complexes (Fig. 6).

Next, we examined the buried areas of our etoposide structures, to help explain why we capture cleaved complexes with either one or two molecules bound, and the observation that etoposide stabilizes both SSB and DSB (Fig. 2c–f). The CRsym complex with one etoposide bound has an area of some 1,653 Å^2^ buried between the two gyrase^CORE^ subunits at the DNA gate (ba_ba'^1–etop^ complex in Fig. 6g). In contrast, the two ‘half-open' Casym complexes with two etoposides bound have buried areas of only 877 and 897 Å^2^ (BA_BA'^2–etop^ and *BA_BA'*^*2–etop*^ complexes in Fig. 6g, respectively). This observation that the DNA gate can close much more fully (and presumably more stably) with one etoposide bound (Fig. 2d) than with two etoposides bound (Fig. 2c, see arrow) gives an explanation of why etoposide gives both SSBs and DSBs with *S. aureus* DNA gyrase (Fig. 2f and Supplementary Fig. 4). The two *S. aureus* gyrase complexes with two etoposides bound (BA_BA'^2–etop^ and *BA_BA'*^*2–etop*^) were both in the half-open Casym conformation. In contrast, *S. aureus* gyrase complexes with two QPT-1 or two moxifloxacins molecules bound can have relatively large areas buried between the two subunits at the DNA gate (1,203–1,526 Å^2^ in Fig. 6g), suggesting that with two QPT-1 or moxifloxacin molecules the DNA gate can close more completely (more stably) than with two etoposides bound (this might be because etoposide is less flat and keeps the −1 and +1 bases further apart). Consistent with the different stabilities of inhibitor complexes observed at the DNA gate, QPT-1 and moxifloxacin were more potent than etoposide in DNA gyrase enzyme assays (Table 2a).

## Discussion

The fluoroquinolones are the only class of topoisomerase inhibitors widely used as antibacterial drugs; however, their utility is threatened by target-mediated resistance. Our work reveals the structural basis for inhibition by QPT-1, the progenitor of an entirely different class of antibacterial^14^. We find that QPT-1, similar to the fluoroquinolones, binds between the +1 and −1 bases at the DNA-cleavage site, blocking the approach of the scissile phosphate to the 3′-hydroxyl of the cleaved DNA and thereby inhibiting DNA re-ligation. However, the QPT-1 binding mode is not identical to fluoroquinolones (which interact with the GyrA subunit via the magnesium ion water bridge) and had not been predicted in modelling studies^12^. In QPT-1, the barbituric acid moiety sits in a pocket between the DNA and protein, and interacts with the main-chain N–H of Asp437 from GyrB; this explains why QPT-1 is active against fluoroquinolone-resistant strains and suggests ways of developing new members of this class. Our observations of six QPT-1-related binding modes (from three QPT-1 cleavage complexes) show that, although the interaction of QPT-1 with the main-chain N–H of Asp437 is conserved, the interaction with the G1 base in more variable (Fig. 4). We propose that QPT-1 can maintain favourable interactions with slightly different binding pockets because: (i) the barbituric acid can adopt different keto/enol tautomers and (ii) the anilino nitrogen of the central ring of QPT-1 can adopt a more planar *sp*^2^ or a more tetrahedral *sp*^3^ conformation. As the barbituric acid is not confined to adopting a pyrimidinetrione tautomer, we suggest that a better generic name for the QPT-1 class of antibacterials would be quinoline barbituric acids.

Hundreds of QPT-1 analogues have been reported in the literature and patents^5^,^13^,^39^. Our structure rationalizes the reported structure–activity relationship, in which the barbituric acid of QPT-1 is essential for antibacterial activity, as this moiety makes a network of adaptable interactions with GyrB and the nucleotides on either side of the cleavage site. Modifications to this region are not well tolerated and frequently lead to significant decrease of antibacterial activity (in-house unpublished data). The (+)-enantiomer of QPT-1 is reported to have greatly reduced DNA gyrase cleavage activity (∼32-fold in Supplementary Fig. 3c) and antibacterial activity^14^ compared with the bioactive (−) QPT-1 enantiomer. This observed preference can be explained from our structure, as the dimethyl morpholino makes interactions with the G1 DNA base (Fig. 3a).

Modelling of AZD0914 into *N. gonorrhoeae* DNA gyrase based on our QPT-1 *S. aureus* gyrase structure rationalizes the low-level, target-mediated resistance reported for AZD0914 (ref. 12 and Fig. 5). Furthermore, our study reveals the binding pocket of QPT-1 overlays with moxifloxacin affording opportunities for scaffold hopping. The overlays suggest that the most variable region between QPT-1 and AZD0914 (Supplementary Fig. 1) could be replaced with substituents from the highly variable C7 position of the fluoroquinolones and related compounds. For example, introducing an aminomethyl pyrolidinyl group at the C7 position has been reported to enhance the activity of fluoroquinolones^40^,^41^; thus, it would be interesting to determine whether introducing the group at a similar position on the QPT-1/AZD0914 scaffold can produce more potent compounds. It might also be interesting to add a siderophore to a QPT-1 analogue, to see whether cell penetration can be improved and possibly to try to mimic the fluoroquinolone water–metal ion bridge^6^,^17^,^18^.

Another promising attribute for antibacterial development of the quinoline barbituric acid series is their excellent selectivity towards bacterial targets. QPT-1 does not have detectable activity against human Top2α (Table 2) and AZD9014 has low activity against human Top2β^42^ despite the fact that the amino acids that QPT-1 contacts are conserved between bacteria and man. To try to understand the bacterial selectivity of QPT-1, we performed an extensive analysis of multiple *S. aureus* DNA gyrase co-crystal structures (see Methods, Supplementary Figs 7 and 9, and Supplementary Table 8). This led us to propose a mechanism for DNA gyrase in which the DNA gate acts like a pair of swing–doors, in that: (i) it tends to remain closed until the T-DNA is pushed through^43^ and (ii) it swings closed immediately after the passage of the T-DNA (Supplementary Fig. 11 and Supplementary Discussion). This is consistent with observations of a closed DNA gate in electron microscopy and small-angle X-ray scattering (SAXS) studies of DNA gyrase complexes with DNA^44^,^45^. Our ‘swing-doors mechanism' for DNA gyrase is an extension of existing ‘standard' models for topo2A cleavage mechanisms^46^,^47^ but differs in a number of respects (as outlined below and detailed in Supplementary Discussion). The ‘swing-doors mechanism' proposes (see Supplementary Fig. 11) that at (a) the DNA gate can gently rock to and fro with G-DNA bound, and that the first cleavage step takes place when movement causes a metal ion (Mg^2+^) to occupy a catalytic ‘A' site (Supplementary Fig. 8). In the absence of a captured T-DNA segment, the tension in the stretched^9^, now singly cleaved G-DNA is then proposed to rock the DNA gate, to religate the cleaved DNA strand. Alternatively, at (b) in the presence of a captured T-DNA segment, and after the first cleavage step, the T-DNA attempts to push the DNA gate open and the enzyme is moved into the Casym ‘half-open' conformation where the second cleavage step now takes place (the phosphotyrosine from the cleaved strand is too far away from the metal-binding site for re-ligation to occur in this ‘half-open' Casym conformation). Thereafter, at (c) the first re-ligation step is proposed to be catalysed by Lys 581 from the conserved Y**K**GLG motif^41^,^48^, which is moved by the T-DNA interacting with the Greek Key domain (Supplementary Figs 12 and 13, the T-DNA is between the DNA gate and exit gate at this stage of the catalytic cycle). It is proposed that this ‘swing-doors' mechanism for DNA gyrase has evolved to limit, to the minimum possible, the amount of time that the gate DNA is cleaved (because both SSBs and DSBs in the DNA can be cytotoxic). Although there are some DNA-gate conformations that appear conserved between prokaryotic and eukaryotic topo2As (Supplementary Fig. 8a–c), the amino acids involved in protein–protein interactions at the DNA gate of prokaryotic and eukaryotic topo2As are not highly conserved (Supplementary Table 7). This lack of sequence conservation seems to be reflected in structural differences, for example, the human Top2β etoposide structure^7^ is in a symmetric ‘dropped trap door'^37^-like conformation, which we have not observed with gyrase, whereas the *S. aureus* gyrase complexes with two etoposides are in the asymmetric (Casym) conformation (which has not been observed in eukaryotic topo2A structures).

**Figure 7:**
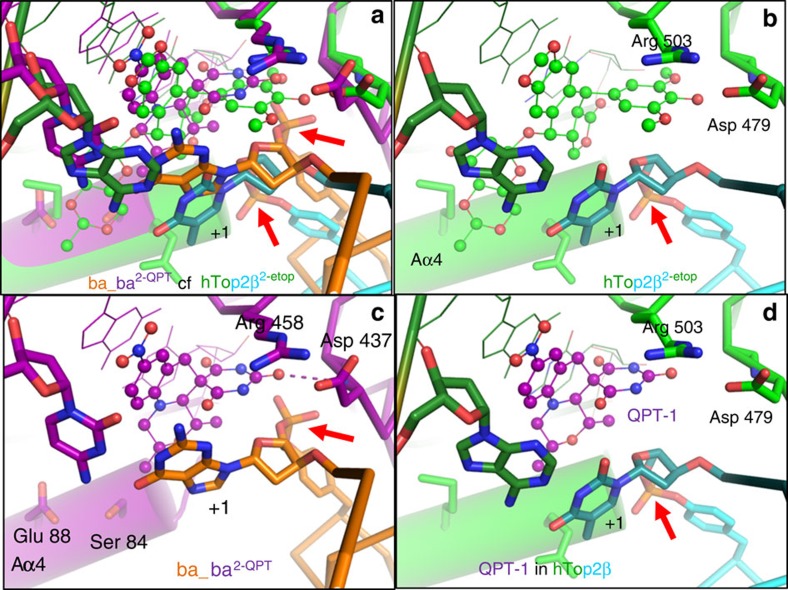
Comparison of a binding site of QPT-1 in *S. aureus* DNA gyrase with the etoposide-binding site in humanTop2β. (**a**) The 2.5-Å QPT-1 complex with *S. aureus* gyrase (ba_ba^2–QPT^) is shown superposed on the 2.16-Å etoposide complex with human Top2β (ref. 7) (pdb code: 3qx3). QPT-1 and etoposide carbons are shown as purple and green balls, respectively. (**b**) The 2.16-Å etoposide complex with human Top2β. The scissile phosphate is arrowed. The Aa4 helix shown as a green cylinder. (**c**) The 2.5-Å QPT-1 complex with *S. aureus* gyrase. The scissile phosphate is arrowed. It is noteworthy that QPT-1 does not contact Ser84 or Glu88 from the Aα4 helix (purple cylinder) but forms a key interaction with Asp437 (dotted lines). (**d**) An illustration of the human Top2β-binding pocket with QPT-1. The protein and DNA from the *S. aureus* complex and the etoposide from the human Top2β complex are not shown. It is noteworthy that the human Top2β pocket seems larger than the pocket in the gyrase complex with QPT-1.

A structural explanation for QPT-1 selectivity comes from a comparison of *S. aureus* QPT-1 and etoposide structures with the human Top2β etoposide structure^7^ (Fig. 7 and Supplementary Figs 10 and 14). This shows the following: (i) a dramatic difference in the position of the scissile phosphates linking the catalytic tyrosine to the cleaved DNA between the human and *S. aureus*-binding pockets (Fig. 7, arrowed) and (ii) a small relative movement of the main-chain N–H of Asp479 of hTop2β (equivalent to Asp437 of *S. aureus* GyrB) away from the key oxygen interaction of QPT-1 (Fig. 7a–c). One outcome of these observations is that with human htop2β the gap in the binding pocket between Asp479 /Arg503 and the +1 nucleotide (which is covalently attached to the catalytic tyrosine by the scissile phosphate) is larger (Fig. 7d versus Fig. 7c), and may be large enough to allow QPT-1 to exit as the DNA gate closes. Although QPT-1 interacts with GyrB residues conserved between bacteria and man, suggesting that bacteria might find it difficult to develop target-mediated drug resistance, laboratory-generated strains of bacteria with resistance to AZD0914 (ref. 12) have mutated some of these highly conserved residues (Fig. 5).

In this study we reveal the first structure of QPT-1 bound to a topo2A–DNA complex, the first structure of an etoposide-inhibited complex with a bacterial topoisomerase and the first model of an asymmetric cleavage complex. We also demonstrate the activity of etoposide and other anticancer agents against bacteria is due to the inhibition of DNA gyrase. Our co-crystal structure of a DNA-cleavage complex for QPT-1 reveals QPT-1 binds at the same site in the cleaved DNA as moxifloxacin; however, unlike the fluoroquinolones, QPT-1 interacts with GyrB TOPRIM residues. This binding rationalizes why QPT-1 is able to overcome target-mediated fluoroquinolone resistance. Furthermore, we provide a structural explanation of QPT-1's bacterial specificity by the observed differences in scissile phosphate positions in human versus bacterial complexes with etoposide. Collectively, we provide new structural insights and strategies for discovering new QPT-1 antibacterials that overcome quinolone resistance, while exploiting knowledge from the fluoroquinolone class.

## Methods

### Compounds

QPT-1 was synthesized using published procedures with slight modifications and the (−) and (+) enantiomers isolated as described^21^ in Supplementary Methods and Supplementary Fig. 16. The bioactive (−) enantiomer of QPT-1 was used in all studies, unless stated. Teniposide was purchased from LKT Labs Inc. Other compounds were obtained from Sigma.

### Protein expression and purification

The *S. aureus* fusion truncates used for crystallography, GyrB27–A56(GKdel) and GyrB27–A56(GKdel/Tyr123Phe), for simplicity renamed gyrase^CORE^ and gyrase^CORE^ (Y123F), respectively, were expressed in *E. coli* BL21 DE3* and purified in four steps as follows^9^: (1) centrifuged cell lysate in lysate buffer (20 mM Tris, 1 mM EDTA, 2 mM dithiothreitol (DTT), 0.2 mM phenylmethylsulfonyl fluoride (PMSF), 1 mM benzamidine, 1 mg ml^−1^ lysozyme, 1 μg ml^−1^ BLAP (a protease inhibitor cocktail consisting of 1 mg ml^−1^ each of bestatin, leupeptin, aprotinin and 2 mg ml^−1^ pepstatin) pH 8.0) was loaded onto a Q Sepharose FF column and washed back to baseline in Buffer 1A (20 mM Tris, 1 mM EDTA, 2 mM DTT, 0.2 mM PMSF, 1 mM benzamidine, 1 μg ml^−1^ BLAP pH 8.0), before being eluted with a gradient from Buffer 1A into Buffer 1B (20 mM Tris, 1 M NaCl, 1 mM EDTA, 2 mM DTT, 0.2 mM PMSF, 1 mM benzamidine, 1 μg ml^−1^ BLAP pH 8.0). (2) The purification pool from the first step was incubated with an additional 10 μl of benzonase for 30 min and then diluted using Buffer 2C (20 mM Tris, 1 mM EDTA, 2 mM DTT pH 8.0) to reduce the conductivity to ∼6 mScm, before being loaded onto a Heparin Sepharose column equilibrated in Buffer 2A (20 mM Tris, 50 mM NaCl, 1 mM EDTA, 2 mM DTT, 0.2 mM PMSF, 1 mM benzamidine, 1 μg ml^−1^ BLAP pH 8.0), and eluted with a gradient into Buffer 2B (20 mM Tris, 1 M NaCl, 1 mM EDTA, 2 mM DTT, 0.2 mM PMSF, 1 mM benzamidine, 1 μg ml^−1^ BLAP pH 8.0) over eight column volumes. (3) The pooled protein from the Heparin Sepharose column was diluted into Buffer 2C to reduce the conductivity to ∼6 mScm, before being loaded onto a 15Q anion exchange column equilibrated in Buffer 2A and elution with a gradient into 40% Buffer 2B over 12 column volumes. (4) The pooled fractions from step 3 were concentrated using a YM30 concentrator (yields and concentrations varied in different preps), before being loaded onto a 320-ml Superdex 200 column equilibrated and eluted in Buffer 4A (20 mM Hepes, 100 mM NaSO_4_, 5 mM MnCl_2_ pH 7.0). Post size-exclusion fractions were pooled and concentrated for crystallization. Full-length *S. aureus* GyrA and GyrB were expressed in *E. coli* and purified as described^9^.

### Gyrase-dependent DNA replication assay

This assay is an indirect method for measuring target potency of bacterial cells that are undergoing DNA replication through gyrase-dependent extension of existing replication forks, as determined by incorporation of ^33^P-dTTP into DNA^33^,^49^. Briefly, *E. coli* ATCC 25922 was grown to log phase (OD_600 nm_∼0.6) at 37 °C, resuspended in one-tenth volume of cold 0.05 M potassium phosphate buffer (pH 7.5) and treated with 1% toluene for 10 min at room temperature, to render cells permeable to nucleotide substrates and cofactors. Toluenized cells were washed in cold buffer and resuspended to 1/25 of the starting volume. DNA gyrase activity was determined by measuring gyrase-dependent DNA replication in the toluenized cells by the ATP-dependent incorporation of radiolabelled ^33^P-TTP. Reactions typically contained at final concentrations per well: 17% v/v toluenized *E. coli* cells, 0.2 μCi ^33^P-TTP, 33 μM dATP, 33 μM dGTP, 33 μM dCTP, 33 μM dTTP, 1.3 mM ATP, 70 mM KPO_4_, 2 mM DTT and 13 mM MgSO_4_. Reactions were incubated at 37 °C for ∼1 h, stopped with 10% trichloroacetic acid and Surface Proximity Assay beads added for scintillation counting.

### DNA cleavage assays

*S. aureus* DNA gyrase cleavage assays were performed according to published methods^9^. Briefly, 250 ng of supercoiled pBR322 plasmid DNA (Topogen) was incubated with either 50 nM of full-length *S. aureus* DNA gyrase or 200 nM of *S. aureus* gyrase^CORE^ protein in 16 μl reactions containing 35 mM Tris-HCl pH 7.5, 24 mM KCl, 4 mM MgCl_2_, 5 mM DTT, 360 μg ml^−1^ BSA, 6.5% (w/v) glycerol and 1.5 mM ATP, in the presence or absence of various concentrations of inhibitor, at 37 °C for 1 h. DNA was released by further incubation with 0.2% (w/v) SDS and 0.1 mg ml^−1^ proteinase K at 37 °C for 60 min. BlueJuice gel loading buffer (Invitrogen) was added. Samples were analysed by electrophoresis in 1% w/v agarose gel containing 0.5 μg ml^−1^ ethidium bromide followed by photography under ultraviolet illumination. DNA bands were quantified by densitometry using UVP gel imaging software. Values were fitted by nonlinear regression with GraFit software (Erythacus). CC_50_, defined as the concentration of compound needed to generate linear DNA to 50% of the maximal level, were determined from a minimum of two independent experiments. Each sample contained compound dissolved in 2% (v/v) dimethylsulfoxide. See Supplementary Fig. 15 for representative gels.

For DNA-cleavage assays of other topoisomerase enzymes, the same general procedure was used as described above with modifications. For *S. aureus* topo IV DNA-cleavage reactions, 30 nM of *S. aureus* topo IV enzyme (Inspiralis) was incubated with 250 ng supercoiled pBR322 (Inspiralis) in a 20-μl reaction supplemented with 1.5 mM ATP according to vendor's instructions. For *E. coli* DNA gyrase-cleavage assay, 100 nM of *E. coli* full-length DNA gyrase subunits A and B (both N-terminally 6-His-tagged) produced in-house (unpublished) were reconstituted at 30 °C for 30 min and then incubated with 250 ng supercoiled pBR322 DNA in a 20-μl reaction containing 35 mM Tris pH 7.5, 24 mM KCl, 4 mM MgCl_2_, 2 mM DTT, 0.1 mg ml^−1^ albumin, 6.5% glycerol, 1.8 mM spermidine and 1.5 mM ATP, and analysed as described^9^. For *E. coli* topo IV DNA-cleavage reaction, 31 nM of *E. coli* topo IV (Inspiralis) was incubated with 250 ng supercoiled pBR322 in buffer supplemented with 1 mM ATP according to the manufacturer's instructions. For human Top2α DNA-cleavage assays, glutathione *S*-transferase-tagged human Top2α was produced as described previously^50^ except Buffer D was modified to contain 350 mM NaCl and 5% glycerol, and *in vitro* cleavage assays performed using 200 nM of the enzyme^50^.

### Topoisomerase inhibition assays

*In vitro* topoisomerase activity assays were performed as described with minor modifications^9^. Briefly, the supercoiling activity of *S. aureus* DNA gyrase was measured in reactions typically containing 3.7 nM of wild-type *S. aureus* DNA gyrase, 500 ng relaxed pBR322 plasmid DNA (Inspiralis), 35 mM Tris-HCl pH 7.5, 24 mM KCl, 700 mM potassium glutamate, 4 mM MgCl_2_, 2 mM DTT, 1.8 mM spermidine, 6.5% (w/v) glycerol, 0.1 mg ml^−1^ BSA and 1 mM ATP. For *E. coli* DNA gyrase supercoiling assays, 3.4 nM of wild-type *E. coli* DNA gyrase (Inspiralis) was incubated with 500 ng relaxed pBR322 plasmid DNA in a 30-μl reaction containing the same buffer mixture but without the potassium glutamate. For *S. aureus* topo IV decatenation assays, 4 nM of *S. aureus* topo IV was incubated with 200 ng kinetoplast DNA in buffer as described by the manufacturer (Inspiralis). In *E. coli* topo IV decatenation assay, 0.125 nM of *E. coli* topo IV was incubated with kDNA as described in the protocol (Inspiralis). Human Top2α relaxation assays were performed as reported using 2 nM of enzyme^50^. All reactions were incubated at 37 °C for 1 h and stopped by the addition of BlueJuice gel loading buffer (Invitrogen). Reactions were analysed by electrophoresis in 1% w/v agarose gels followed by ethidium bromide staining. IC_50_ values, defined as the amount of compound that causes half-maximal inhibition, were determined by quantifying the DNA products using densitometry (VisionWorks, UVP) and fitting the data by nonlinear regression with GraFit software (Erythacus). Compound IC_50_ values reported are the averages of two to three determinations.

### Minimum inhibitory concentrations

Antibacterial minimum inhibitory concentrations (MICs) were determined from two independent experiments using broth microdilution methods according to Clinical and Laboratory Standards Institute guidelines^51^. The MIC was the lowest concentration of an antibacterial that showed no visible growth after incubation at 37 °C for 18–24 h, with a starting inoculum of ∼5.5 × 10^5^ colony-forming units per ml. Bacterial strains used were from GSK's culture collection.

### Resistant mutant isolation

Bacterial cells were plated onto Mueller–Hinton agar plates (for *S*. *aureus* RN4220) or Mueller–Hinton+5% sheep blood agar plates (for *S*. *pneumoniae* 1629) containing 2 × or 4 × MIC of etoposide. After 48 h incubation at 37 °C, resistant colonies were purified on etoposide-containing plates and then on plates without etoposide. The quinolone resistance-determining region (QRDR) or full-length of *gyrA*/*B* and *parC*/*E* were amplified by PCR from the resistant mutants and DNA sequenced using the following primer pairs (all 5′–3′ sequence): for *S. aureus gyrA*: CGTTGTAGAAAACCGTAGACA and GGAATTTCAGTGACAACA; *gyrB* CAAACATGGTGATCCTCA and CGGTCTCATAAATCGATAGAAG; *parC* GTATGCAAGAGGACCAAAGT and CCTTTACCTGATTCATAAGCT, and *parE* CGTGAAGGTTTAACAGCTGTTGTG and CTCTTCGTCTGTCCAAGC, respectively. For *S. pneumoniae gyrA*: AGCACCATCACCGACAAG and ATCCCAACCGCGATACCAG; *gyrB*: CAAGATTACCAATCGCCTCTT and CTGCTTCTGCAGCATCATCTA; *parC*: CTTAAGGGAGCGGTTAAGAC and TCTTGGAACGAACAACCACG, and *parE*: AGTAGCCCTCCAGTACAATG and ACGTGTTTCTGGGTTCATGG, respectively. *S*. *pneumoniae* 1629 genomic DNA harbouring a GyrB L407–S410 deletion or R447C mutation were transformed into *S. pneumoniae* R6 strain to regenerate mutants resistant to etoposide according to the procedures described^52^, except AGCH was replaced by THY (Todd–Hewitt+0.5% yeast extract) medium. Mutations in etoposide-resistant transformants were confirmed by PCR and DNA analysis.

### Refinement of DNA complexes

The optimization of DNA sequences, crystallization and initial structure determinations of the six complexes are described in an accompanying paper^22^.To refine structures, cycles of rebuilding and refinement were carried out with coot^53^, Refmac^54^, Phenix^55^ and Buster^56^, to give models with good geometry (Supplementary Table 1). The Ramachandran plots of the structures determined had the following percentages of residues in Ramachandran favoured and Ramachandran outlier regions: (i) 97.7 and 0.15% 2.5 Å QPT-1 structure (pdb code 5CDM), (ii) 96.3 and 0.04% 3.15 Å QPT-1 structure (pdb code 5CDO), (iii) 97.5% and 0.26% 2.8 Å etoposide structure (pdb code 5CDN), (iv) 98.1 and 0.08% 2.45 Å etoposide structure (pdb code 5CDP), (v) 96.7 and 0.64% 2.95 Å moxifloxacin structure (pdb code 5CDQ), and (vi) 97.6 and 0.0% 2.65 Å binary complex (pdb code: 5CDR).

To help determine which QPT-1 tautomer was bound at each of the six QPT-1-binding sites in the three QPT-1 complexes, the compounds were removed at the end of refinement and redocked with Afitt^57^. Afitt has four criteria for evaluating a docking pose: (i) a real space correlation coefficient for the fit of the pose to the electron density and (ii) a ligand strain score (the lower the better) and two scores of the ligand protein interactions (piece-wise linear potential (PLP) and Chemscore, based only on heavy, non-hydrogen, atom positions). The differences between the heavy (non-hydrogen) atom positions in the eight possible QPT-1 tautomers are quite small; thus, in docking runs all eight tautomers often gave similar binding poses in a particular binding site with only small differences in real space correlation coefficient (typically ∼0.65–0.68). In Afitt, differences between neutral QPT-1 tautomers with hydroxyl (O–H) groups on the barbituric acid and the equivalent unprotonated (O^−^) compound (Supplementary Table 9) were evident only in the PLP and Chemscore values. The central ring of QPT-1 contains a nitrogen, which can be either *sp*^2^ or *sp*^3^—an ‘anilino-type' nitrogen. The degree of ligand strain of a particular pose depended, to some extent, on the forcefield used in the run, either MMff94 (anilino nitrogen restraints *sp*^3^) or MMff94s (anilino nitrogen restraints *sp*^2^). Differences in the relative positions of atoms in the different tautomeric forms of QPT-1 are not large and the resolutions of our *S. aureus* gyrase QPT-1 complexes are not high; thus, there is some experimental uncertainty in the QPT-1 tautomers, which we have tentatively assigned to the different QPT-1-binding sites (Fig. 4, Supplementary Fig. 6, and Supplementary Tables 2 and 9). Barbituric acid oxygens, which point towards a delta-negative region of a *π*-cloud of a base pair, may be more likely to adopt a protonated (enol form), whereas if they point towards a delta-positive region of the *π*-cloud they may be more likely to adopt the unprotonated (keto form)^25^. We have not carried out detailed calculations on the electrostatics of interactions between the different tautomeric forms of QPT-1 and the surrounding bases.

In the 2.5-Å QPT-1 complex ba_ba'^2–QPT^, both binding sites appeared to have the same binding mode and we modelled in tautomer 1 (Fig. 4c, Supplementary Fig. 6c and Supplementary Tables 2, 9A) with the nitrogen in the central ring restrained to be *sp*^2^ hybridized. The 3′-OH of the cleaved DNA is close enough to donate a hydrogen bond to the side chain of Glu 435', suggesting that the water between the 3′-OH and the scissile phosphate most probably accepts a hydrogen bond from an N–H group on the barbituric acid of QPT-1. Docking studies suggested that one of the QPT-1 molecules in the 3.15 Å *BA*_*BA*'^*2–QPT*^ complex exists with the nitrogen in the central ring in an *sp*^3^ configuration, accepting an internal hydrogen bond from an O–H on the barbituric acid moiety, and we modelled in tautomer 6 (Fig. 4d, Supplementary Fig. 6c, and Supplementary Tables 2 and 9C) into this binding site. We modelled tautomer 4 into the other QPT-1-binding site in the asymmetric *BA*_*BA'*^2–QPT^complex and tautomers 5 and 6 into the two binding sites in the BA_BA'^2–QPT^ complex (Fig. 4, Supplementary Fig. 6c and Supplementary Table 9; all three have the central ring nitrogen in a planar *sp*^2^ configuration; Supplementary Table 2). Two of the oxygens on the barbituric acid point towards bases in all the QPT-1-binding sites and interact with *π*-electrons from the bases. In tautomers 4, 5 and 6 (Fig. 4, Supplementary Fig.6 and Supplementary Table 2), one or both of the oxygens pointing at bases have been modelled as hydroxyls (enol rather than ketone tautomer).

### Structural analysis

To analyze the different configurations observed at the DNA gate, structures were superposed using Cα atoms from subsets of residues corresponding to core secondary structural elements from the catalytic TOPRIM and winged helical domains (residues underlined in Supplementary Fig. 5). The structures were superposed and root mean square (RMS) fits were calculated with LSQKAB^58^. Areas buried between protein subunits at the DNA gate (Fig. 6 and Supplementary Fig. 9) were calculated with Pisa^38^ as implemented in Coot and are ‘interface areas', defined as half-sum of surface area that becomes inaccessible to solvent when subunits form the interface.

The analysis (Supplementary Fig. 7) showed two clusters of structures: CRsym and Casym. Five structures formed the CRsym cluster with relatively large areas buried between the two subunits at the DNA gate (1,625–1,959 Å^2^). In the CRsym structures the angle between two subunits at the DNA gate is 179.4–179.9° (Supplementary Table 8). The structures in the CRsym cluster have either one or no compounds bound and are three NBTI (GSK299423) complexes^9^ with intact DNA (Y123F mutation), the 2.45-Å etoposide complex with the doubly nicked DNA and only one compound (ba_ba'^1–etop^), and a 2.65-Å binary complex with doubly nicked DNA and no compound (ba_ba'). In the binary complex the four DNA base pairs stretched^9^ between the two cleavage sites are not well ordered. The second cluster Casym contained three asymmetric structures (angle between two subunits at DNA gate 173.5±0.6°) and had the smallest areas buried between the two subunits at the DNA gate (852–897 Å^2^). The three structures in the Casym cluster were the two 2.80 Å etoposide-cleavage complexes (BA_BA'^2–etop^ and *BA_BA'*^*2–etop*^) and one of the 3.15-Å QPT-1-cleavage complexes (*BA_BA'*^*2–QPT*^). The remaining four cleavage complexes, two QPT-1 complexes (ba_ba'^2–QPT^ and BA_BA'^2–QPT^) and the two 2.95-Å moxifloxacin-cleavage complexes (BA_BA'^2–moxi^ and *BA_BA'*^*2–moxi*^), do not form a cluster but have a range of configurations intermediate between those of the two clusters and also have buried areas intermediate between those observed for the two clusters (1,203–1,526 Å^2^).These four intermediate structures had angles between domains at the DNA gate of 177.5–179.9° and RMS fits of 0.732–0.902 Å (excluding the two moxifloxacin complexes, which were quite similar to each other and had an RMS fit 0.282 Å; Supplementary Table 8).

### Modelling of AZD0914 in *N. gonorrhoeae* DNA gyrase

A homology model of the DNA-cleavage gate of the Gram-negative *N. gonorrhoeae* DNA gyrase was built using domains from the crystal structure of the DNA gate of *E. coli* DNA gyrase (pdb code: 3NUH^59^). The domains of *N. gonorrhoeae* DNA gyrase were superposed on the equivalent domains in the 2.5 Å *S. aureus* DNA gyrase complex with QPT-1. Homology and chemical modelling were carried out in MOE^60^ and the structures were minimized using the mmff94 forcefield.

## Additional information

**Accession codes**: Structures have been deposited in the Protein Data Bank under accession numbers 5CDM, 5CDO, 5CDN, 5CDP, 5CDQ and 5CDR.

**How to cite this article:** Chan, P. F. *et al.* Structural basis of DNA gyrase inhibition by antibacterial QPT-1 anticancer drug etoposide and moxifloxacin. *Nat. Commun.* 6:10048 doi: 10.1038/ncomms10048 (2015).

## Supplementary Material

Supplementary Information Supplementary Figures 1-16, Supplementary Tables 1-9, Supplementary Discussion, Supplementary Methods and Supplementary References

## Figures and Tables

**Table 1:** Summary of six co-crystal structures.

	**QPT-1-2.5**	**QPT-1-3.15**	**Etop-2.8**	**Etop-2.45**	**Moxi-2.95**	**Binary**
PDB CODE	5CDM	5CDO	5CDN	5CDP	5CDQ	5CDR
Compound	QPT-1	QPT-1	Etoposide	Etoposide	Moxifloxacin	None
*S. aureus* gyrase^core^ protein	Wild type	Wild type	Wild type	GyrA Y123F	Wild type	GyrA Y123F
Cleavage complex*	Yes	Yes	Yes	No	Yes	No
DNA	20–447 T	20–447 T	20–447	20–12p-8	20–448 T	20–12p-8
Space group	P6_1_	P2_1_	P2_1_	P6_1_	P2_1_	P6_1_
Resolution (Å)	2.50	3.15	2.80	2.45	2.95	2.65
No. of compounds bound per complex	2	2	2	1	2	0
No. of complexes in AU	1	2	2	1	2	1
Names of complexes in AU†	ba_ba'^2–QPT^	BA_BA'^2–QPT^ *BA_BA'*^*2*–*QPT*^	BA_BA'^2–etop^ *BA_BA'*^*2*–*etop*^	ba_ba'^1–etop^	BA_BA'^2–moxi^ *BA_BA'*^*2*–*moxi*^	ba_ba'

AU, asymmetric unit; Etop, etoposide; Moxi, moxifloxacin; QPT-1, quinoline pyrimidine trione-1.

See Supplementary Table 1 and Srikannathasan *et al.*^22^ for crystallographic details.

^*^The four cleavage complexes in the Table are all double-strand cleaved with a covalent phosphotyrosine bond linking the cleaved DNA to the protein (Y123). Crystallization with DNAs, with artificial nicks in the DNA at both cleavage sites, more readily gives crystals; two of the complexes in the Table have nicked DNA, these are not cleavage complexes.

^†^DNA complexes were named with BA or ba representing one covalently fused B27-A56 gyrase^CORE^ subunit and BA' or ba' the second subunit in the dimer. In the space group P2_1_, the second complex is distinguished from the first by being in *italics*. The number of molecules and the name of the inhibitor bound in each complex are indicated in suffix. Hence, the two QPT-1 cleaved complexes in the P2_1_ cells are identified as BA_BA'^2–QPT^ and *BA_BA'*^*2*–*QPT*^, and the one in the P6_1_ cell as ba_ba'^2–QPT^.

**Table 2:** Target potencies and antibacterial activities of inhibitors.

**a**	**CC_50_ or IVR IC**_**50**_ (μM)*		
	**QPT-1**	**Etoposide**	**Moxifloxacin**
*DNA cleavage*			
* S. aureus* DNA gyrase†	8.8	107	2.5‡
* *Human Top2α	>800	21	>800
* E. coli* DNA gyrase	8.6	113	0.6‡
*E. coli* gyrase-dependent IVR assay	0.34	4.3	0.17

**b**	**MIC (μg** **ml**^−**1**^**)**		
	**QPT-1**	**Etoposide**	**Moxifloxacin**
*S*. *aureus* RN4220	0.5	64	0.063
*S*. *aureus* RN4220 GyrB D437N	4	128	0.063
*S*. *aureus* RN4220 GyrB D437A	2	256	0.063
*S*. *pneumoniae* 1629	—	2	0.031
*S*. *pneumoniae* 1629 GyrB R447C	—	16	0.094
*S. pneumoniae* 1629 GyrB ΔL407–S410	—	16	0.047
*E. coli* 7623	8	>256	≤0.016
*E. coli* 7623 ΔtolC	0.063	0.75	≤0.016

IC_50_, half-maximal inhibitory concentration; IVR, *in vitro* DNA replication; MIC, minimum inhibitory concentration; QPT-1, quinoline pyrimidine trione-1; ‘—', not measured.

(**a**) Activity in topoisomerase biochemical assays.

(**b**) Antimicrobial susceptibility against *S. aureus*, *S. pneumoniae* and *E. coli* strains.

See Supplementary Tables 3–6 for a full set of enzyme activity and MIC data.

^*^CC_50_ is defined as amount of compound that causes half-maximal induction of linear DNA (double-stranded DNA breaks). IC_50_ is the amount of compound required to inhibit 50% of maximal DNA replication activity. Values are averages of at least two independent studies.

^†^Full-length, wild-type form of *S. aureus* DNA gyrase protein.

^‡^DNA cleavage data from Black *et al.*^61^.

**Table 3:** Antimicrobial susceptibility to anticancer topo2A inhibitors.

***S. aureus*** **strain**	**MIC(μg** **ml**^−**1**^**)**							
	**Etoposide**	**Teniposide**	**Doxorubicin**	**Daunorubicin**	**Mitoxantrone**	**Amsacrine**	**Ellipticine**	GSK299423 (NBTI)*
RN4220 parent	64	16	8	8	32	>128	1	0.016
RN4220 GyrB D437N†	128	32	8	8	32	>128	1	**0.125**
RN4220 GyrB D437A†	**256**	32	8	8	*8*	*32*	1	**0.125**
RN4220 GyrB D437V†	**256**	**64**	8	8	*8*	*64*	1	**0.125**
RN4220 GyrB P456L†	**256**	32	4	4	*4*	*32*	1	**0.125**
RN4220 GyrB ΔT403-K406	**256**	32	3	*1.5*	*6*	128	1	0.016

MIC, minimum inhibitory concentration; topo2A, type IIA topoisomerase.

See Supplementary Table 6 for MIC data to DNA gyrase etoposide-resistant mutants in *S. pneumoniae.*

MIC values are averages of two independent determinations. Significant (≥4-fold) resistance and hypersensitivity are highlighted in bold and italic, respectively.

^†^Mutants isolated with NBTI.

^*^GSK299423 is a control compound.
