# Supplementary material for: Structural basis of DNA gyrase inhibition by antibacterial QPT-1, anticancer drug etoposide and moxifloxacin
Source: Nat Commun. 2015 Dec 7;6:10048. doi: 10.1038/ncomms10048 (PMC4686662; doi:10.1038/ncomms10048)
Supplement: Supplementary Information — Supplementary Figures 1-16, Supplementary Tables 1-9, Supplementary Discussion, Supplementary Methods and Supplementary References [file ncomms10048-s1.pdf]

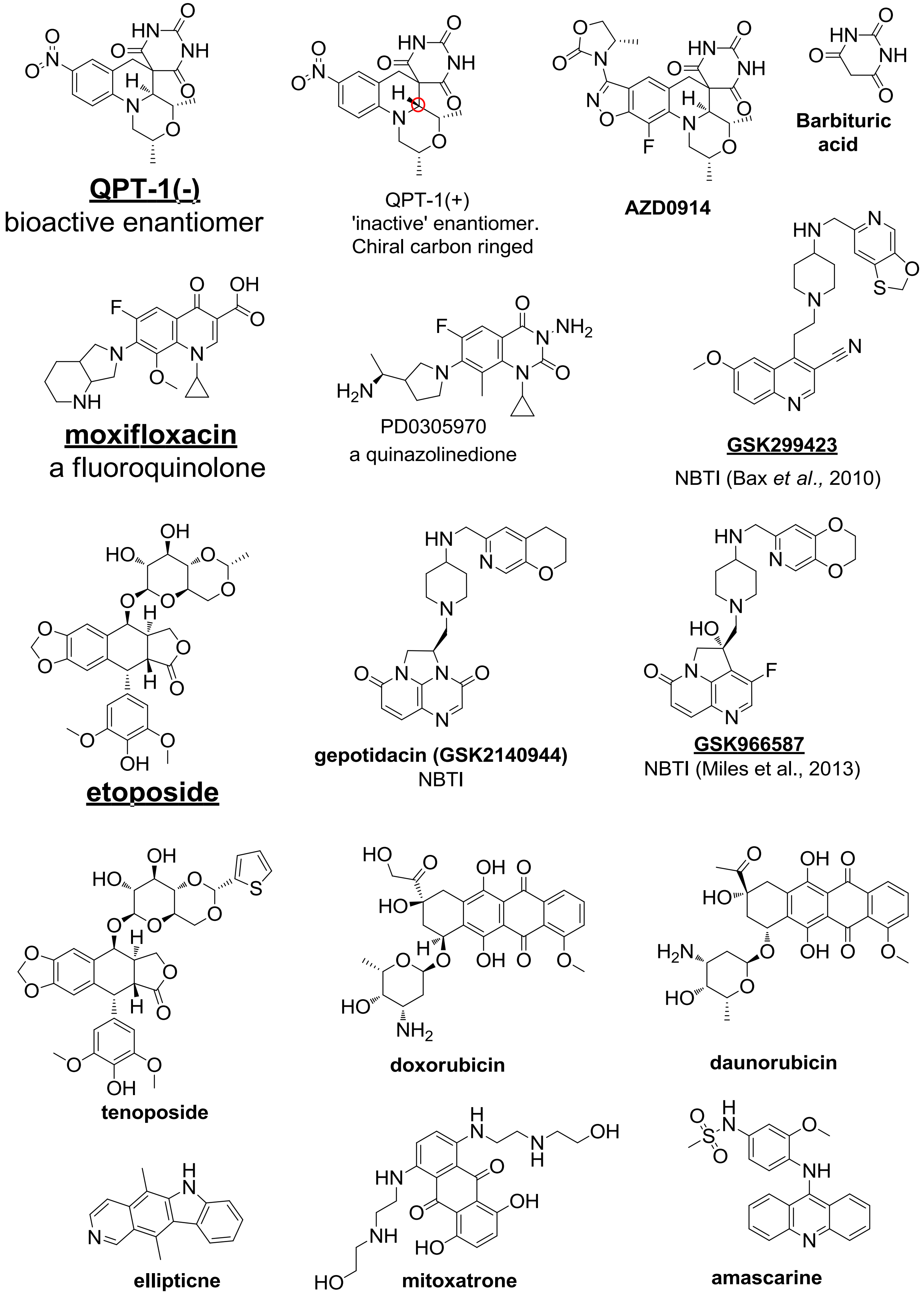

Supplementary Fig. 1. Structures of compounds. Compounds co-crystallised with *S.aureus* DNA Gyrase and DNA at GSK are underlined.

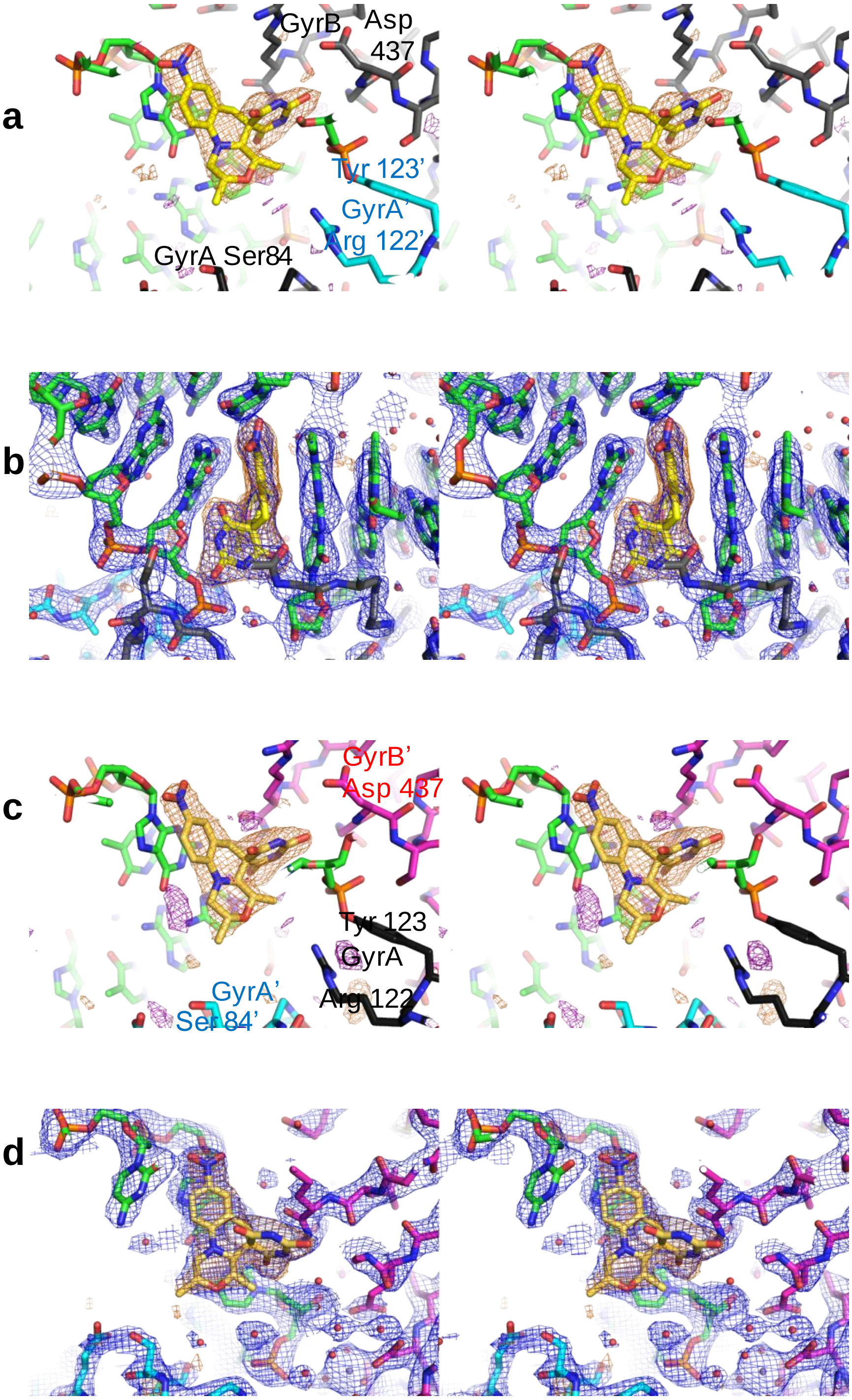

Supplementary Fig. 2. Electron density (stereo) for compounds: 2.5 Å QPT-1 structure (ba_ba’ $^{2\text{-QPT}}$ complex). (a,b) Fo-Fc density (plus 3 sigma – orange, minus 3 sigma - purple) and final 2Fo-Fc density (1.5 sigma blue) for the QPT-1 site adjacent to the GyrB b subunit in the 2.5 Å QPT-1 structure (ba_ba’ $^{2\text{-QPT}}$). (**c,d**) Fo-Fc density (plus 3 sigma – orange, minus 3 sigma - purple) and final 2Fo-Fc density (1.0 sigma blue) for the QPT-1 site adjacent to the GyrB b’ subunit (the 2nd monomer) in the 2.5 Å QPT-1 structure.

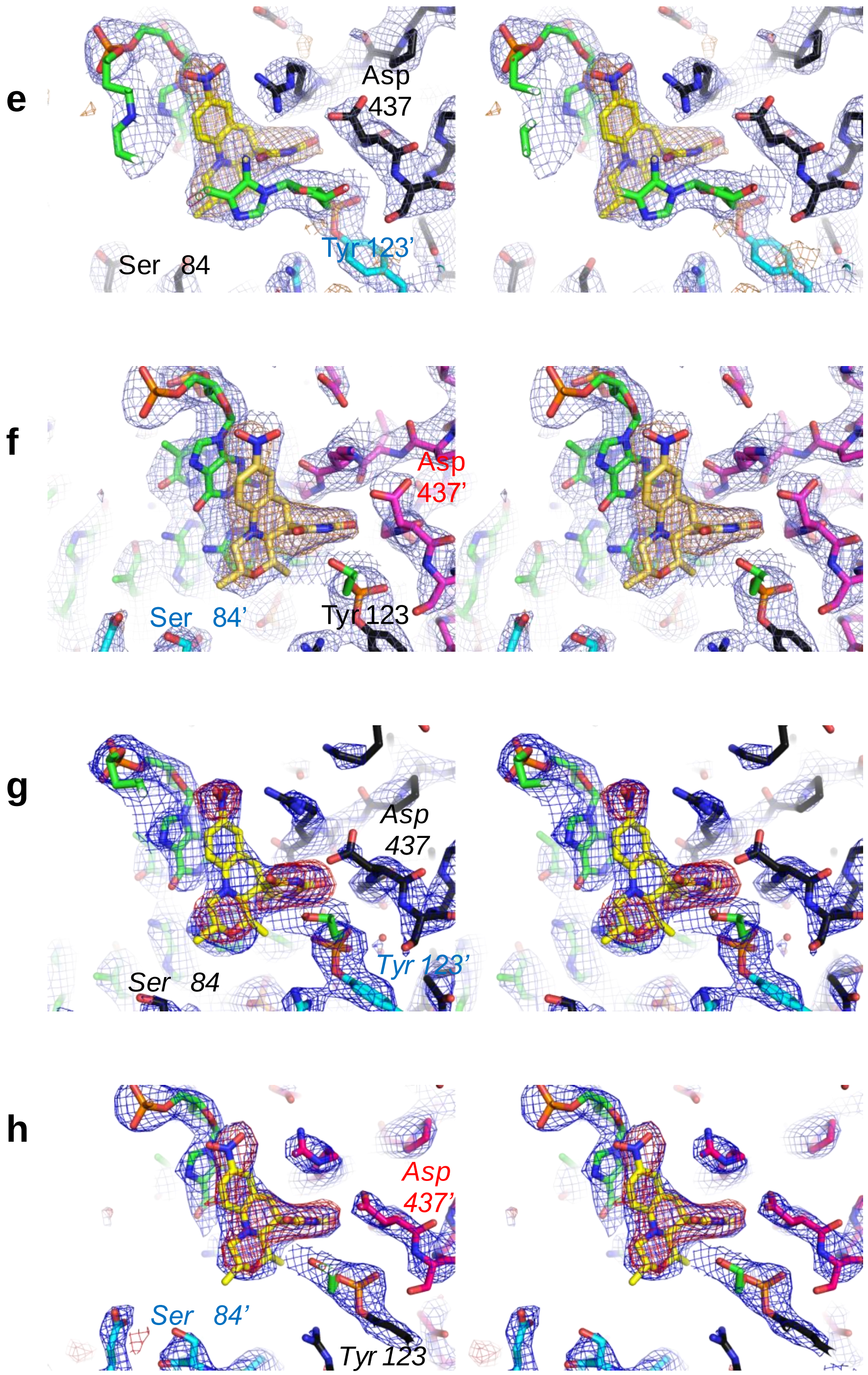

Supplementary Fig. 2. Electron density (stereo) for compounds (continued): 3.15 Å QPT-1 structure, two complexes in asymmetric unit (BA_BA'$^{2\text{-}QPT}$ and *BA_BA'$^{2\text{-}QPT}$*). **(e,f)** Fo-Fc density (plus 3 sigma – orange) and final 2fo-Fc density (1.0 sigma blue) for the QPT-1 site adjacent to the GyrB B (e) and B' (f) subunits of the BA_BA'$^{2\text{-}QPT}$. **(g,h)** Fo-Fc density (plus 3 sigma – red) and final 2Fo-Fc density (1.0 sigma blue) for the QPT-1 site adjacent to the *GyrB B* (g) and *B'* (h) subunits of the *BA_BA$^{2\text{-}QPT}$* complex.

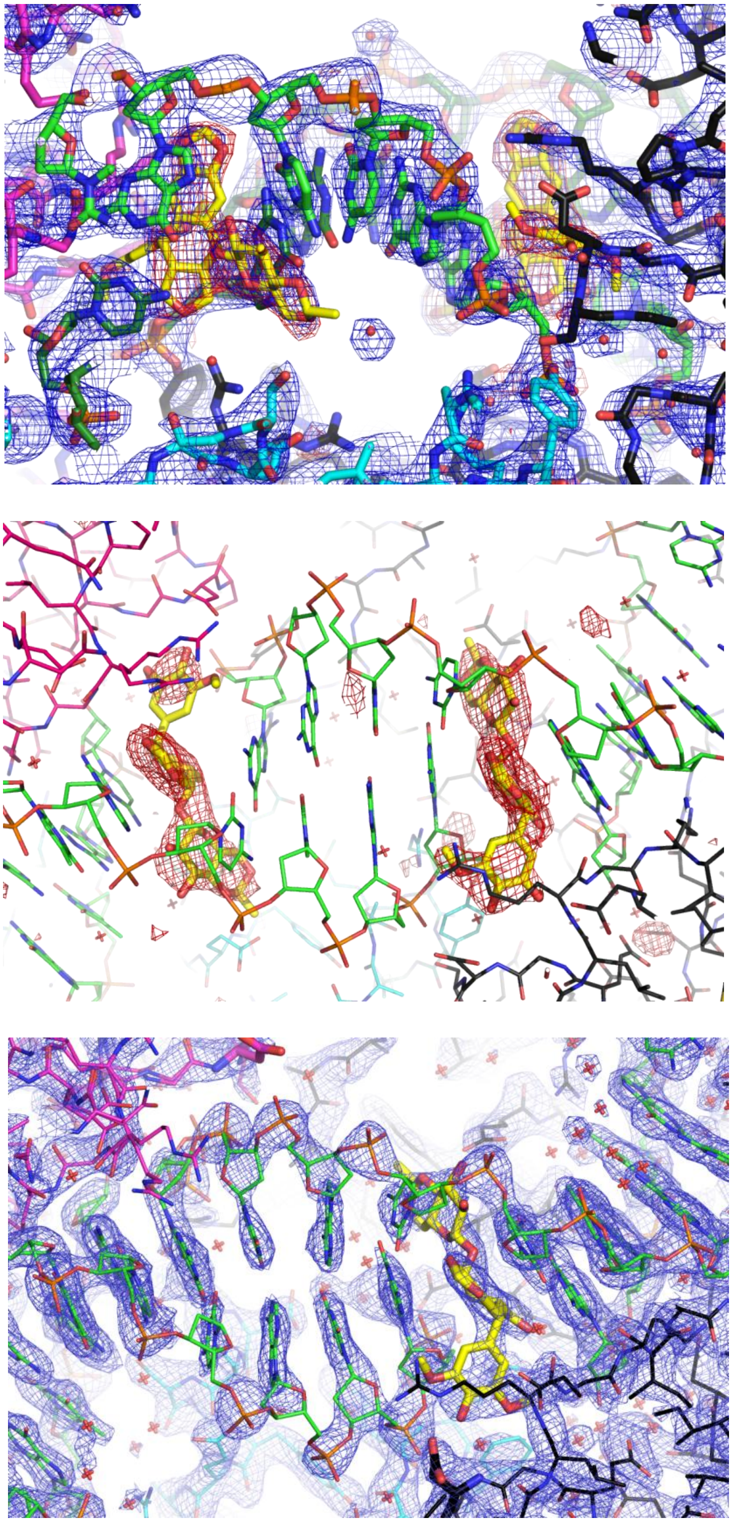

Supplementary Fig. 2. Electron density for compounds (cont'd): 2.8 Å etoposide structures (BA_BA'$^{2\text{-etop}}$ and *BA_BA'*$^{2\text{-etop}}$ complexes) and 2.45 Å etoposide structure (ba_ba'$^{1\text{-etop}}$ complex). (i) Fo-Fc density (plus 3 sigma – red) and final 2Fo-Fc density (1.0 sigma blue) for the etoposide sites in the BA_BA'$^{2\text{-etop}}$ complex. (**j**) Fo-Fc density (plus 3 sigma – red) for the etoposide sites in the *BA_BA*^{2etop} complex. (**k**) Final 2Fo-Fc density (1.5 sigma - blue) for the 2.45 Å ba_ba'$^{1\text{-Etop}}$ complex.

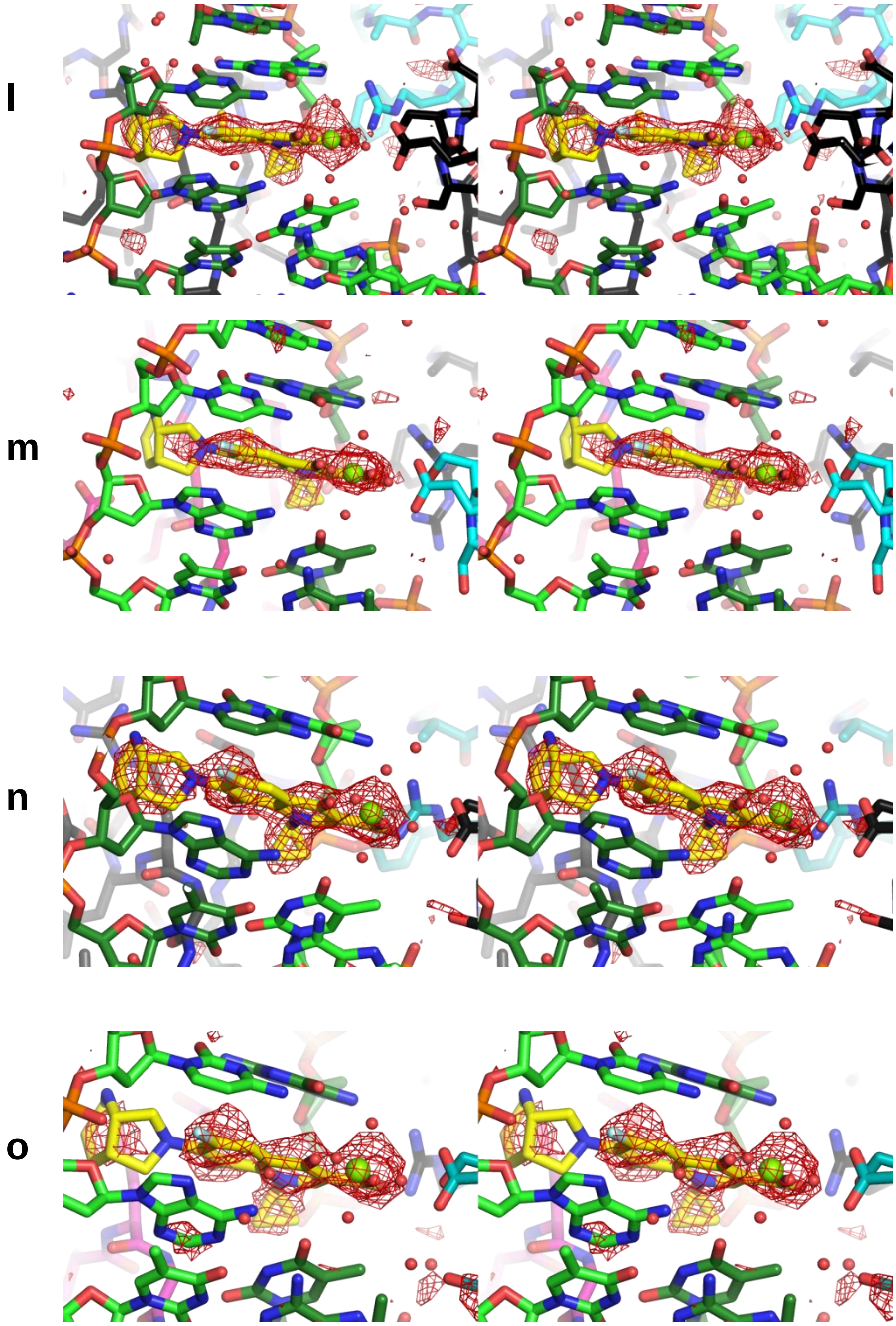

Supplementary Fig. 2. Electron density (stereo) of compounds (cont'd): 2.95 Å moxifloxacin structure (BA_BA'$^{2\text{-}moxi}$ and *BA_BA'$^{2\text{-}moxi}$* complexes).
(l,m,n,o) Stereo Fo-Fc density (plus 3 sigma – red) for the moxifloxacin sites adjacent to the GyrB B (l), B' (m), *B* (n) and *B'* (o) subunits in the 2.95 Å moxifloxacin structure.

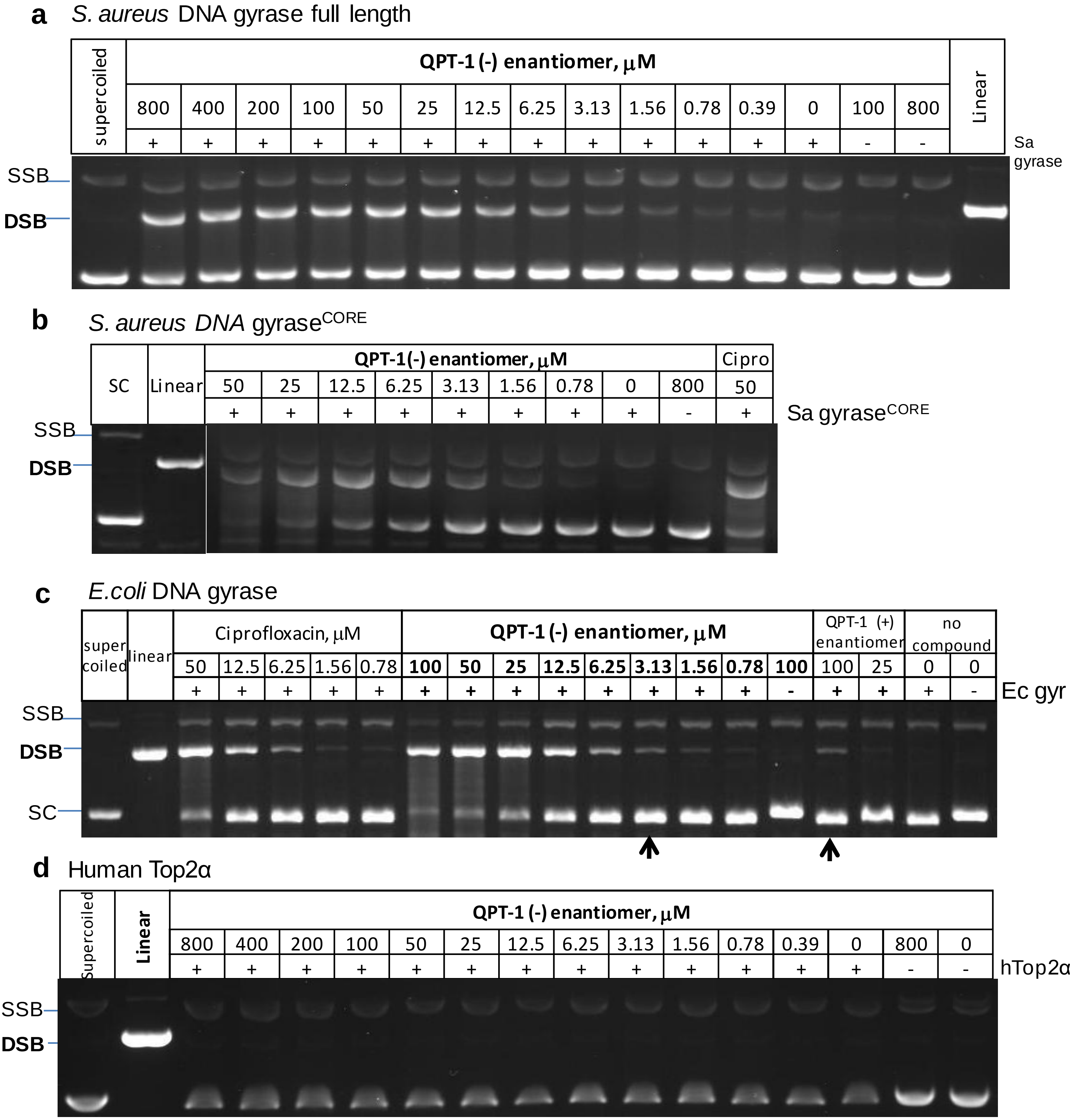

Supplementary Fig. 3. QPT-1 induces double-stranded breaks with DNA gyrase but not with human Top2α. DNA cleavage gel assays in which QPT-1 (at concentrations shown) was incubated with supercoiled pBR322 in the presence of **(a)** *S. aureus* DNA gyrase, **(b)** *S. aureus* DNA gyrase^{CORE} , **(c)** *E.coli* DNA gyrase, **(d)** Human Top2α. DNA cleavage assays were performed as described in Methods. 200 nM of each enzyme was used. The intensities of the linear DNA bands (DSB) were determined by densitometry, and CC_{50} fitted on these values using Grafit program. CC_{50} of QPT-1 were 8.8, 3.2, 8.6 and > 800 μM against *S. aureus* gyrase full length, *S. aureus* gyrase^{CORE}, *E. coli* gyrase and human Top2α enzymes, respectively**.** Ciprofloxacin or etoposide (not shown) were included as positive controls. Note, in Fig (c) the (-) enantiomer of QPT-1 was ~ 30-fold more active in DNA cleavage activity than the (+) enantiomer (arrows mark concentrations showing similar intensities of DSB). SC, supercoiled; SSB, single-stranded breaks; DSB, double-stranded breaks; +, with enzyme; -, no enzyme.

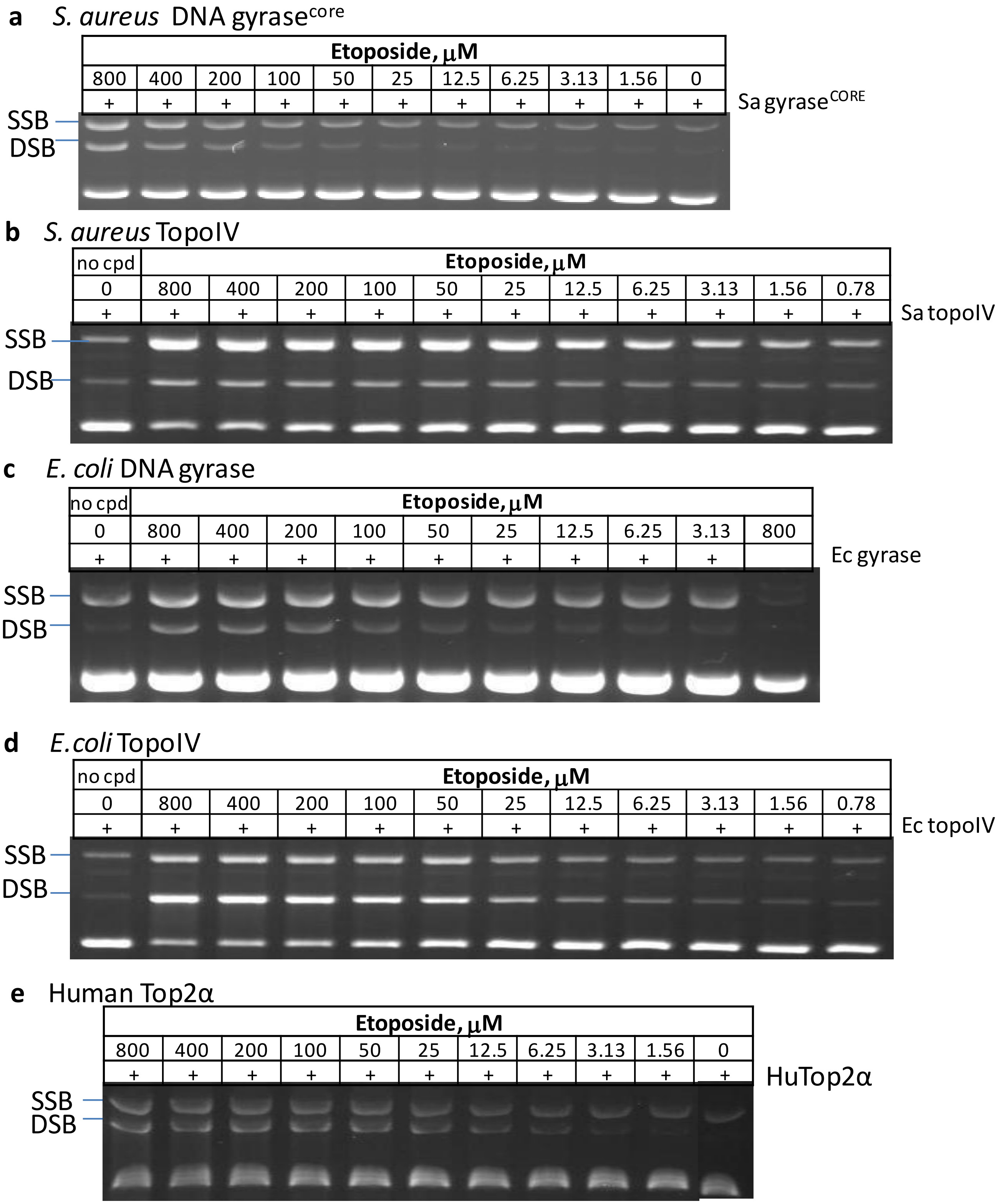

Supplementary Fig. 4. Etoposide induces double- and single-stranded breaks with bacterial and human topo2As.
DNA cleavage gel assays in which etoposide (at concentrations shown) was incubated with supercoiled pBR322 in the presence of **(a)** *S.aureus* DNA gyrase^core (construct used for crystallography), (**b**) *S. aureus* TopoIV, (**c**) *E. coli* DNA gyrase, (**d**) *E. coli* TopoIV and (**e**) Human Top2α as described in Methods (SSB, single-stranded breaks; DSB, double-stranded breaks). Etoposide induces both single-stranded DNA breaks (SSB) and double-stranded DNA breaks (DSB) with *S. aureus* DNA gyrase^core , *S.aureus* TopoIV, *E.coli* Gyrase, *E.coli* TopoIV and human Top2α enzymes giving CC_{50} for DSB of 144, 126, 113, 103 and 21 μM respectively. Values are average of two independent experiments. +, with enzyme; -, no enzyme.

Supplementary Fig. 5. Multiple alignment of amino acid sequences highlighting residues at the DNA-gate.

Legend on next page

```
                              Bβ1          Bβ2   *   Bα2            Bβ3               Bα  TOPRIM Structure
SaGyrB   rksaldvaslpgKLADCSSKSPEECEIFLVEGDSAGGSTKSGR---DSRTQAILPLRGKILNVEKARLDRI  B472   QPT-1
TbGyrB   RKSATDIGGLPGKLADCRSTDPRKSELYVVEGDSAGGSAKSGR---DSMFQAILPLRGKIINVEKARIDRV  B496   Etop.
EcGyrB   rkgaldlaGLPGKLADCQERDPALSELYLVEGDSAGGSAKQGR---NRKNQAILPLKqkilnvekarfdkm  B461   Etop.'
SpParE   KKNKKDKGLLSGKLTPAQSKNPAKNELYLVEGDSAGGSAKQGR---DRKFQAILPLRGKVVNTAKAKMADI  E470   NBTI
AbParE   rkkivsgpALPGKLADCVGQTREESELFIVEGDSAGGSAKQAR---DKNFQAIMPIRGKILNTWEVSSDEV  E432   Moxi.
ScTopII  RKSRITNYPKLEDANKAGTKEGYKCTLVLTEGDSALSLAVAGLAVVGRDYYGCYPLRGKMLNVREASADQI  489    3LJ4
HuTOP2A  KHNRIKGIPKLDDANDAGGRNSTECTLILTEGDSAKTLAVSGLGVVGRDKYGVFPLRGKILNVREASHKQI  501    4FM9
HuTOP2B  KYSKIKGIPKLDDANDAGGKHSLECTLILTEGDSAKSLAVSGLGVIGRDRYGVFPLRGKILNVREASHKQI  517  Etop.(3QX3)
Conser.                                EGDSA                  PLRGK   N

         2'  Bα3                        Bβ4    * *    Bα4                Bα5     Bβ  TOPRIM Structure
SaGyrB   LNNNEIRQMITAFGTGI----GGDFDLAKARYHKIVIMTDADVDGAHIRTLLLTFFYRFMRPLIEAG-YVY  B538   QPT-1
TbGyrB   LKNTEVQAIITALGTGI----HDEFDIGKLRYHKIVLMADADVDGQHISTLLLTLLFRFMRPLIENG-HVF  B562   Etop.
EcGyrB   LSSQEVATLITALGcgi---grdeynpdklRYHSIIIMTDADVDGSHIRTLLLTFFYRQMPEIVERG-HVY  B528   Etop.'
SpParE   LKNEEINTMIYTIGAGV----GADFSIEDANYDKIIIMTDADTDGAHIQTLLLTFFYRYMRPLVEAG-HVY  E536   NBTI
AbParE   LASQEVHDIAIAIGVDP----GSD-DLSELRYGKICILADADSDGLHIATLLCALFVKHFPALVEEG-HLY  E497   Moxi.
ScTopII  LKNAEIQAIKKIMGLQHRKKYEDT---KSLRYGHLMIMTDQDHDGSHIKGLIINFLESSFPGLLDIQGFLL  557    3LJ4
HuTOP2A  MENAEINNIIKIVGLQYKKNYEDEDSLKTLRYGKIMIMTDQDQDGSHIKGLLINFIHHNWPSLLRHR-FLE  571    4FM9
HuTOP2B  MENAEINNIIKIVGLQYKKSYDDAESLKTLRYGKIMIMTDQDQDGSHIKGLLINFIHHNWPSLLKHG-FLE  587  Etop.(3QX3)
Conser.      E         G                   Y         D  D  DG HI   L

         5     Bβ6         Bβ7    Bα6            Bβ8          Bα8           Bβ  T(GK)M Sructure
SaGyrB   IAQPPlykltqgk----qkyyvyndreldklkselnptpkwsiarYKGLGEMNADQLWETTMNPEHRALLQ  B605   QPT-1
TbGyrB   LAQPPLYKLKWQR---SDPEFAYSDRERDGLLEAGL[8]EDGIQRYKGLGEMDAKELWETTMDPSVRVLRQ  B635   Etop.
EcGyrB   IAQPPLYKVKKGK----QEQYIKDDEAMDQYQIS[174]GLSIQRYKGLGEMNPEQLWETTMDPESRRMLR  B764   Etop.'
SpParE   IALPPLYKMSKGKGKKEEVAYAWTDGELEELRKQFG--KGATLQRYKGLGEMNADQLWETTMNPETRTLIR  E605   NBTI
AbParE   VAMPPLFRIDIGKDV----HYALDDEELETILKNVKGNKNPQITRFKGLGEMNAIQLRETTMDPNTRRLVQ  E564   Moxi.
ScTopII  EFITPIIKVSITKPTK-NTIAFYNMPDYEKWREEESHKFTWKQKYYKGLGTSLAQEVREYFSNLDRHLKIF  627    3LJ4
HuTOP2A  EFITPIVKVSKNK----QEMAFYSLPEFEEWKSSTPNHKKWKVKYYKGLGTSTSKEAKEYFADMKRHRIQF  638    4FM9
HuTOP2B  EFITPIVKASKNK----QELSFYSIPEFDEWKKHIENQKAWKIKYYKGLGTSTAKEAKEYFADMERHRILF  654  Etop.(3QX3)
Conser.      P                                           YKGLG       E

         9    Bα9           Bα10                                Aβ0     Aα1   TOPRIM Structure
SaGyrBA  VKLEDAIEADQTFEMLMG-DVVENRRQFIEDNAVYanldf*----*maelpqsriNERNITSEMRESFLDY  A25    QPT-1
TbGryBA  VTLDDAAAADELFSILMG-EDVDARRSFITRNAKDVRFLDV*-*[7]PDDSLDRIEPVDIQQEMQRSYIDY  A31    Etop.
EcGyrBA  VTVKDAIAADQLFTTLMG-DAveprrafieenalkaanidi*----*msdlareitpvnieeelkSSYLDY  A24    Etop.'
SpParEC  VTIEDLARAERRVNVLMG-DKVEPRRKWIEDNVKFTLEETTVF*------*MSNIQNMSLEDIMGERFGRY  C20    NBTI
AbParEC  LDLDDAHLTAGLLDKLLAKKRAADRKQWLEQKgnladitv*----*mtslahhateNRSVAEFTEQAYLNY  C25    Moxi.
ScTopII  HSLQ--GNDKDYIDLAFSKKKADDRKEWLRQYEPG-------------TVLDPTLKEIPISDFINKELILF  683    3LJ4
HuTOP2A  KYSG--PEDDAAISLAFSKKQIDDRKEWLTNFMEDRR-QRKLLGLPEDYLYGQTTTYLTYNDFINKELILF  706    4FM9
HuTOP2B  RYAG--PEDDAAITLAFSKKKIDDRKEWLTNFMEDRR-QRRLHGLPEQFLYGTATKHLTYNDFINKELILF  722  Etop.(3QX3)
Conser.                              R

         Aα1            Aα2             Aβ1   Aα3              Aα4            WHD   Structure
SaGyrA   AMSVIVARALPDVRDGLKPVHRRILYGLNEQGMTPDKSYKKSARIVGDVMGK-YHPHGDSSIYEAMVRMAQD  A96    QPT-1
TbGryA   AMSVIVGRALPEVRDGLKPVHRRVLYAMFDSGFRPDRSHAKSARSVAETMGN-YHPHGDASIYDSLVRMAQP  A102   Etop.
EcGyrA   AMSVIVGRALPDVRDGLKPVHRRVLYAMNVLGNDWNKAYKKSARVVGDVIGK-YHPHGDSAVYDTIVRMAQP  A95    Etop.'
SpParC   SKYIIQDRALPDIRDGLKPVQRRILYSMNKDSNTFDKSYRKSAKSVGNIMGN-FHPHGDSSIYDAMVRMSQN  C91    NBTI
AbParC   AMYVIMDRALPHISDGLKPVQRRIVYAMSELGLKSSGKPKKSARTVGDVLGK-YHPHGDSACYEAMVLMAQP  C96    Moxi.
ScTopII  SLADNI-RSIPNVLDGFKPGQRKVLYGCFKKNL---KSELKVAQLAPYVSECTAYHHGEQSLAQTIIGLAQN  751    3LJ4
HuTOP2A  SNSDNE-RSIPSMVDGLKPGQRKVLFTCFKRND---KREVKVAQLAGSVAEMSSYHHGEMSLMMTIINLAQN  774    4FM9
HuTOP2B  SNSDNE-RSIPSLVDGFKPGQRKVLFTCFKRND---KREVKVAQLAGSVAEMSAYHHGEQALMMTIVNLAQN  790  Etop.(3QX3)
Conser.          R   P    DG  KP   R                 K A                HG              Q

               Aβ2                **  Aβ3   Aα7         Aβ4      Aβ5     WHD   Structure
SaGyrA   FS--YRYPLVDGQGNFGS-MDG-DGAAAMRYTEARMTKITLELLRDINKDTIDFIDNYDGNEREPSVLPAR  A163   QPT-1
TbGryA   WS--LRYPLVDGQGNFGS-PGN-DPPAAMRYTEARLTPLAMEMLREIDEETVDFIPNYDGRVQEPTVLPSR  A169   Etop.
EcGyrA   FS--LRYMLVDGQGNFGS-IDG-DSAAAMRYTEIRLAKIAHELMADLEKETVDFVDNYDGTEKIPDVMPTK  A162   Etop.'
SpParC   WK--NREILVEMHGNNGS-MDG-DPPAAMRYTEARLSEIAGYLLQDIEKKTVPFAWNFDDTEKEPTVLPAA  C158   NBTI
AbParC   FS--YRYPLIEGQGNWGS-PDDPKSFAAMRYTEAKLSAYSELLLSELGQGTSEWQDNFDGSLKEPITLPAR  C164   Moxi.
ScTopII  FVGSNNIYLLLPNGAFGTRATGGKDAAAARYIYTELNKLTRKIFHPADDPLYKYIQ-EDEKTVEPEWYLPI  821    3LJ4
HuTOP2A  FVGSNNLNLLQPIGQFGTRLHGGKDSASPRYIFTMLSSLARLLFPPKDDHTLKFLY-DDNQRVEPEWYIPI  844    4FM9
HuTOP2B  FVGSNNINLLQPIGQFGTRLHGGKDAASPRYIFTMLSTLARLLFPAVDDNLLKFLY-DDNQRVEPEWYIPI  860  Etop.(3QX3)
Conser.           L     G  G          A   RY                          D     EP
```

```
        Aα9   Aβ6      Aβ7      Aα10             Aα10'            Aβ8  Aα10"  WHD    Structure
SaGyrA  FPNLLANGASGIAVGMATNIPPHNLTELINGVLSLSKNPDISIAELMEDIEGPDFPT-AGLILGKSGIRRA A233  QPT-1
TbGryA  FPNLLANGSGGIAVGMATNIPPHNLRELADAVFWALE[7]ETLAAVMGRVKGPDFPT-AGLIVGSQGTADA A243  Etop.
EcGyrA  IPNLLVNGSSGIavgmATNIPPHNLTEVINGCLAYIDDEDISIEGLMEHIPGPDFPT-AAIINGRRGIEEA A232  Etop.'
SpParC  FPNLLVNGSTGISAGYATDIPPHNLAEVIDAAVYMIDHPTAKIDKLMEFLPGPDFPT-GAIIQGRDEIKKA C228  NBTI
AbParC  VPNILLNGTTGIAVGMATDIPPHNLREVVKGTIALIRNPQTSDEKLAEYIPAPDLPTKAEIITPPEELLKI C235  Moxi.
ScTopII LPMILVNGAEGIGTGWSTYIPPFNPLEIIKNIRHLMNDEEL----------EQMHPWFRGWTGTIEEIEP- 881   3LJ4
HuTOP2A IPMVLINGAEGIGTGWSCKIPNFDVREIVNNIRRLMDGEEP----------LPMLPSYKNFKGTIEELAP- 904   4FM9
HuTOP2B IPMVLINGAEGIGTGWACKLPNYDAREIVNNVRRMLDGLDP----------HPMLPNYKNFKGTIQELGQ- 920  Etop.(3QX3)
Conser.   P  L NG  GI  G     P                         P

        "   Aβ9    Aβ10      Aβ11        Aα4               Aβ12          Tower  Structure
SaGyrA  YETGRGSIQMRSRAVIEERGGGRQRIVVTEIPFQVNKARMIEKIAELVRDKK---IDGITDLRDETSLRTG A301  QPT-1
TbGryA  YKTGRGSIRMRGVVEVEEDSRGRTSLVITELPYQVNHDNFITSIAEQVRDGK---LAGISNIEDQSSDRVG A311  Etop.
EcGyrA  YRTGRGKVYIRARAEVEV[4]gRETIIVHEIPYQVNKARLIEKIAELVKEKR---VEGISALRDESDK-DG A300  Etop.'
SpParC  YETGKGRVVVRSKTEIEKLKGGKEQIVIIEIPYEINKANLVKKIDDVRVNNK---VAGIAEVRDESDRD-G C295  NBTI
AbParC  QTTGRGSYRMRAVYTIE-----KNEIVITELPYQVSGSKVITQIADQMQAKK---LPLVVDVRDESDHENP C298  Moxi.
ScTopII ----LRYRMYGRIEQIG-----DNVLEITELPARTWTSTIKEYLLLG-LSGNDKIKPWIKDMEEQHDD--N 940   3LJ4
HuTOP2A ----NQYVISGEVAILN-----STTIEISELPVRTWTQTYKEQVLEPMLNGTEKTPPLITDYREYHTDT-T 965   4FM9
HuTOP2B ----NQYAVSGEIFVVD-----RNTVEITELPVRTWTQVYKEQVLEPMLNGTDKTPALISDYKEYHTDT-T 981  Etop.(3QX3)
Conser.                              E P

          Aβ13      Aα12      Aβ14   Aβ15  Aβ16   Aα14   ..  Tower/WHD..  Structure
SaGyrA  VRVVIDVRKD-ANASVILNNLYKQTPLQTSFGVNMIALVN-GRPKLIN-LKEA A351          QPT-1
TbGryA  LRIVIEIKRD-AVAKVVINNLYKHTQLQTSFGANMLAIVD-GVPRTLR-LDQL A361          Etop.
EcGyrA  MRIVIEVKRD-AVGEVVLNNLYSQTQLQVSFGINMVALHH-GQPKIMN-LKDI A350          Etop.'
SpParC  LRIAIELKKD-ANTELVLNYLFKYTDLQINYNFNMVAIDN-FTPRQVG-IVPI C345          NBTI
AbParC  TRLVIVLRSNRIDAEAVMSHLFATTDLESSYRVNLNMIGEDGRPQVKS-IRRI C350          Moxi.
ScTopII IKFIITLSPEEMAKTRKIGFYERFKLISPISLMNMVAFDPHGKIKKYNSVNEI 993           3LJ4
HuTOP2A VKFVVKMTEEKLAEAERVGLHKVFKLQTSLTCNSMVLFDHVGCLKKYDTVLDI 1018          4FM9
HuTOP2B VKFVVKMTEEKLAQAEAAGLHKVFKLQTTLTCNSMVLFDHMGCLKKYETVQDI 1034         Etop.(3QX3)
Conser.
```

Supplementary Fig. 5. Multiple alignment of amino acid sequences highlighting residues at the DNA-gate. (cont.)

The figure shows a structurally based alignment of the amino acid sequences of: *S.aureus* GyrB/GyrA (SaGyrBA), *M. tuberculosis* GyrBA (TbGyrBA), *E. coli* GyrB/GyrA (EcGyrBA), *S. pneumoniae* ParE/ParC (SpParEC), *A. baumannii* ParE/ParC (AbParEC), *S. cerevisiae* topoII (ScTopII), human topoIIα (HuTOP2A) and human topoIIβ (HuTOP2B). The residues in the Conser. line are conserved in all eight sequences in the alignment (the five baterial topo2As and the three eucayotic topo2As).

Amino acids are highlighted on the five bacterial sequences if they contact (< 3.8 Å) either the compound (orange), or the DNA (green), or make protein:protein interactions (blue) with the other subunit at the DNA gate. Purple residues contact both DNA and the opposing subunit. If a residue contacts a compound it is in orange, even if it also contacts DNA or opposing protein subunit.

The contacts mapped onto the five bacterial sequences are from *S.aureus* DNA gyrase complexes with four different classes of inhibitors: QPT-1 = contacts from the ba subunit in the 2.5Å ba_ba'$^{2\text{-}QPT}$ structure (pdb code 5CDM). **Note that two key interactions with QPT-1 come from two highly conserved sequence motifs in the TOPRIM domain of GyrB, EGDSA and PLR/KGK (R/K is a conservative Arginine or Lysine change).** Etop. = contacts from the BA subunit in the 2.8Å BA_BA'$^{2\text{-}etop}$ complex (first, BA, subunit). Etop.' = contacts from the BA' subunit in the 2.8Å BA_BA'$^{2\text{-}etop}$ complex (second, BA', subunit) (pdb code 5CDN). NBTI = contacts from the BA subunit in the 3.5Å BA_BA'$^{1\text{-}NBTI}$ complex (pdb code 2XCR). Moxi. = contacts from the BA subunit in the 2.95Å BA_BA'$^{2\text{-}moxi}$ complex (note for moxifloxacin the Mg^{2+} ion and waters of the water ion bridge were counted as part of the compound (pdb code 5CDQ). The contacts in the 3.25Å *A. Baumannii* TopoIV moxifloxacin complex structure, 2XKK, are not shown but are nearly identical to those from the 2.95Å *S.aureus* moxifloxacin complex – (here mapped onto the *A. baumanii* TopoIV sequences in the alignment). Note, because the BA_BA'$^{2\text{-}etop}$ complex is not C2 symmetric the contacts from the BA and BA' subunits are not the same.

The contacts mapped onto the three eucaryotic structures are from two binary complexes with DNA (pdb codes: 3LJ4 and 4FM9) and the 2.16Å etoposide complex with human top2β (pdb code: 3QX3).

The secondary structural elements in *S. aureus* gyrase complex with DNA and GSK299423 are shown above the sequence alignment (Bax *et al*., 2010), * or * above the sequence alignment indicate the positions of the catalytic residues (Glu B435, Asp B508, Asp B510, Arg A122, Tyr A123) in *S. aureus* GyrB or GyrA. Residues underlined on the SaGyrBA sequence (in the WHD and TOPRIM domains) were used in superpositions (Supplementary Table 8). When the DNA has been cleaved the catalytic tyrosine (Tyr 123 in *S.aureus* Gyrase A) is covalently attached to the scissile phosphate, and the residue was considered as a phosphotyrosine when calculating contacts.

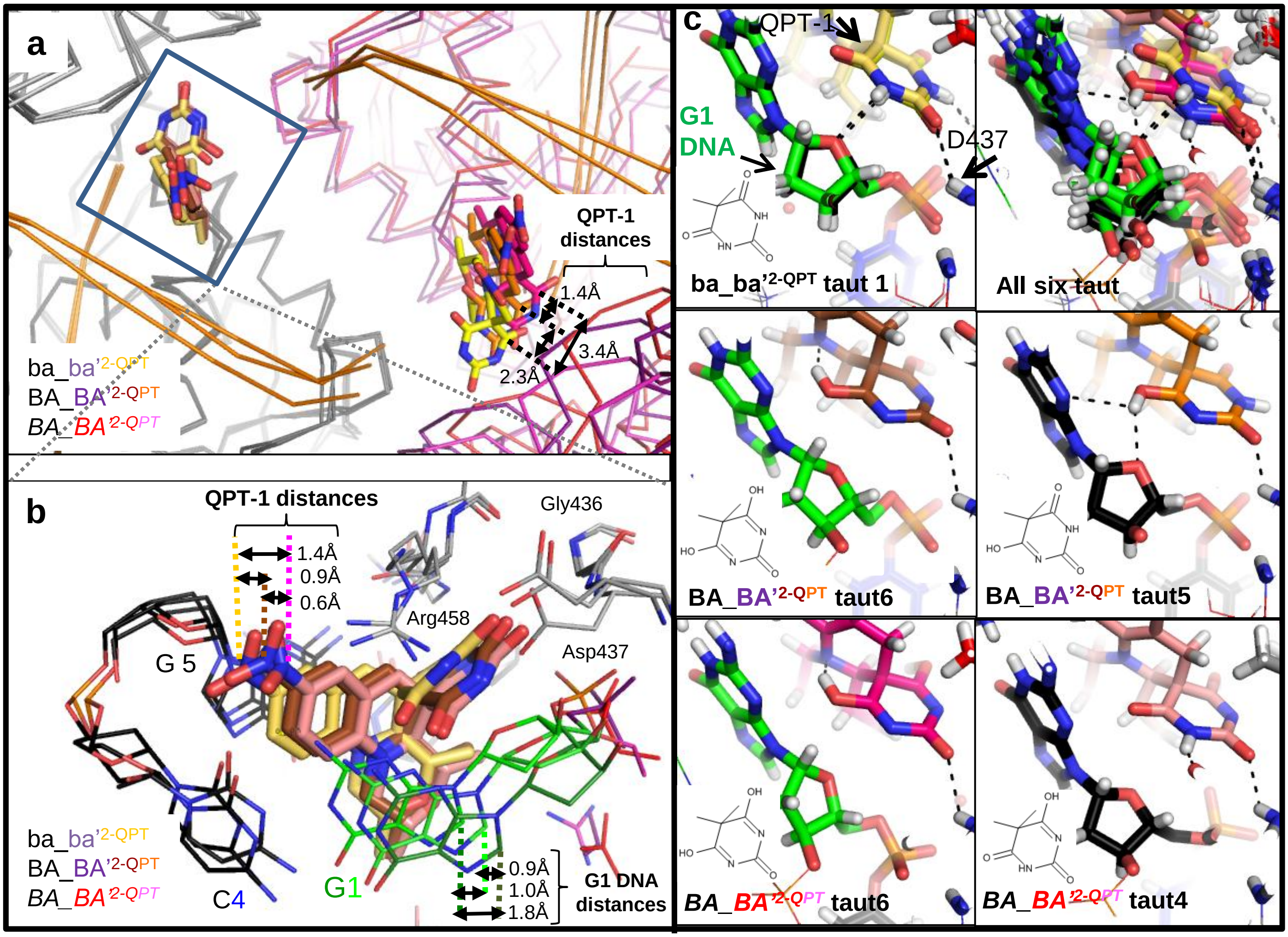

Supplementary Fig. 6. Comparison of QPT-1 binding sites with different tautomeric states. (**a**) The three QPT-1 cleaved complexes ba_ba'$^{2\text{-QPT}}$, BA_BA'$^{2\text{-QPT}}$ and *BA_BA'*$^{2\text{-QPT}}$ from 2.5Å and 3.15 Å QPT-1 crystal structures, each with two QPT-1 molecules bound, were superposed using the ba (BA, BA) subunits (grey) (see Methods for details). Relative shifts between the two subunits at the dimer interface means the QPT-1 inhibitors adjacent to the ba' (BA', BA') subunits have moved relative to each other by between 1.4 and 3.4 Å (bottom right of panel a). (**b**) An enlarged view from a different angle of the three QPT-1 binding sites adjacent to the ba (BA, BA) subunits (boxed in panel a). Differences in the relative positions of QPT-1 (0.6 to 1.4 Å) and of the guanosine nucleotide at the +1 position (G1 DNA, green) (0.9 to 1.8Å) are highlighted. (**c**) Multiple tautomeric states of QPT-1 interacting similarly with the main-chain N-H of the conserved Asp437 residue of GyrB (right edge of panels), but differently with the guanosine nucleotide (G1) at the +1 position. Note the variability in the position of the guanosine nucleotide and the phosphate linking it to the catalytic tryosine. As the G1 base moves, the tautomers on QPT-1 may change. Tautomers of QPT-1 used in docking experiments are also shown in Supplementary Table 2.

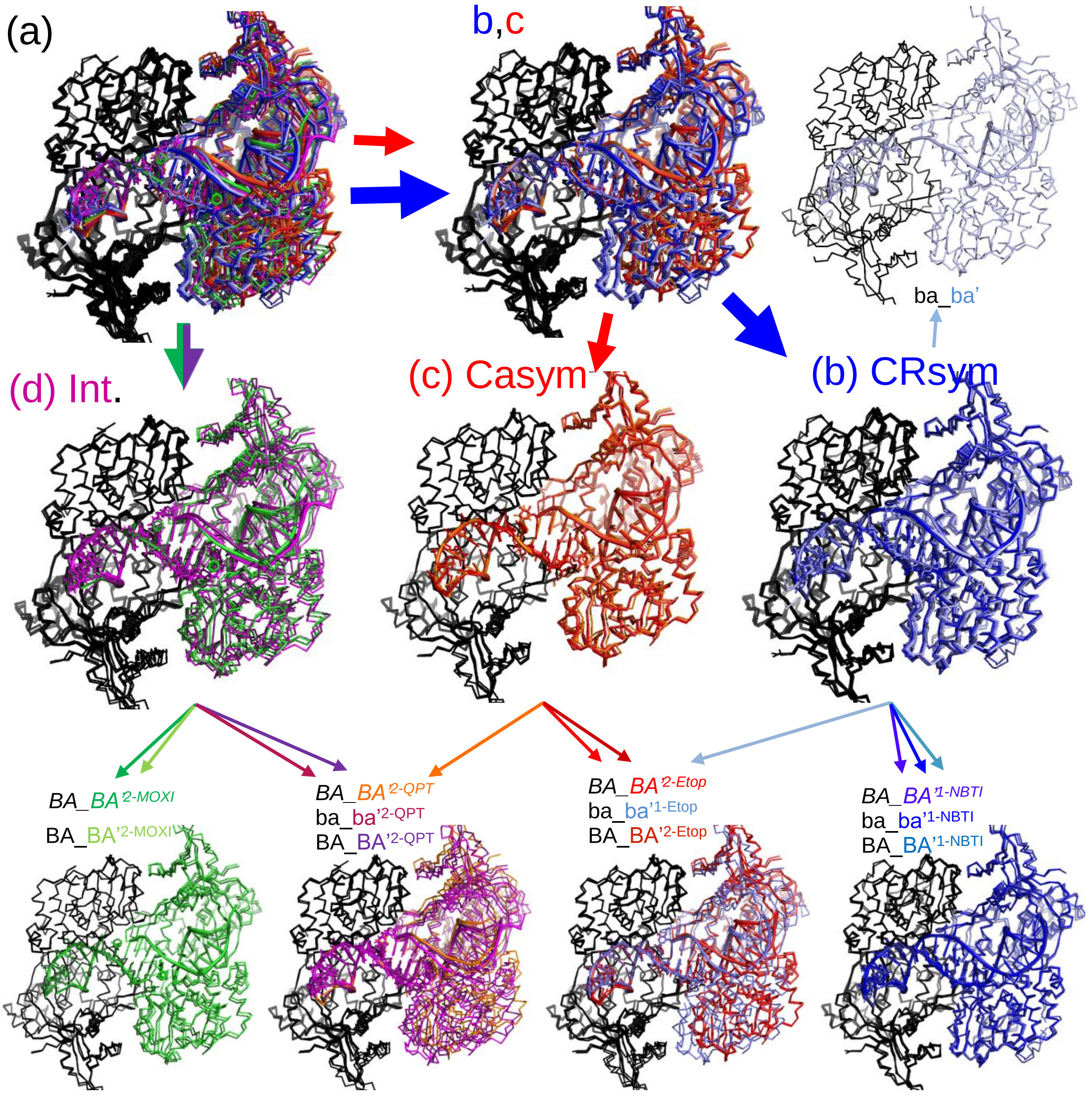

Supplementary Fig. 7. Comparison of DNA gate in twelve *S. aureus* gyrase complexes with DNA. (**a**) Twelve *S. aureus* gyrase complexes with DNA were superposed using the ba (BA or *BA*) subunits (black) – see Methods and Supplementary Table 8 for details. The second subunits in each complex, ba’ (BA’ or *BA’*) vary in position. The twelve structures can be broken into two major clusters, **b** and **c**, and a third group, **d**. (**b**) **CRsym cluster** contains five structures (in blue). Of these, three are complexes with uncleaved DNA and the NBTI GSK299423 (ba_ba’$^{1\text{-NBTI}}$, BA_BA’$^{1\text{-NBTI}}$, *BA_BA’*$^{1\text{-NBTI}}$), a complex with a doubly nicked DNA and one etoposide bound (ba_ba’$^{1\text{-Etop}}$), and a binary complex with doubly nicked DNA and no compound bound, (ba_ba’). **(c) Casym cluster** contains three structures (in red/orange). Of these, two are cleavage complexes with two etoposide molecules bound (*BA_BA’*$^{2\text{-Etop}}$, BA_BA’$^{2\text{-Etop}}$) and the third a cleavage complex with two QPT-1 molecules bound (*BA*_BA’$^{2\text{-QPT}}$). **(d) Intermediate** - the four remaining cleavage complexes (two QPT-1 complexes, ba_ba’$^{2\text{-QPT}}$, BA_BA’$^{2\text{-QPT}}$, and the two moxifloxacin complexes, *BA_BA’*$^{2\text{-MOXI}}$, BA_BA$^{2\text{-MOXI}}$) do not form a cluster but have a range of configurations between those of the two clusters. CRsym = cleavage and/or religation competent symmetrical conformation. Casym = common asymmetrical conformation.

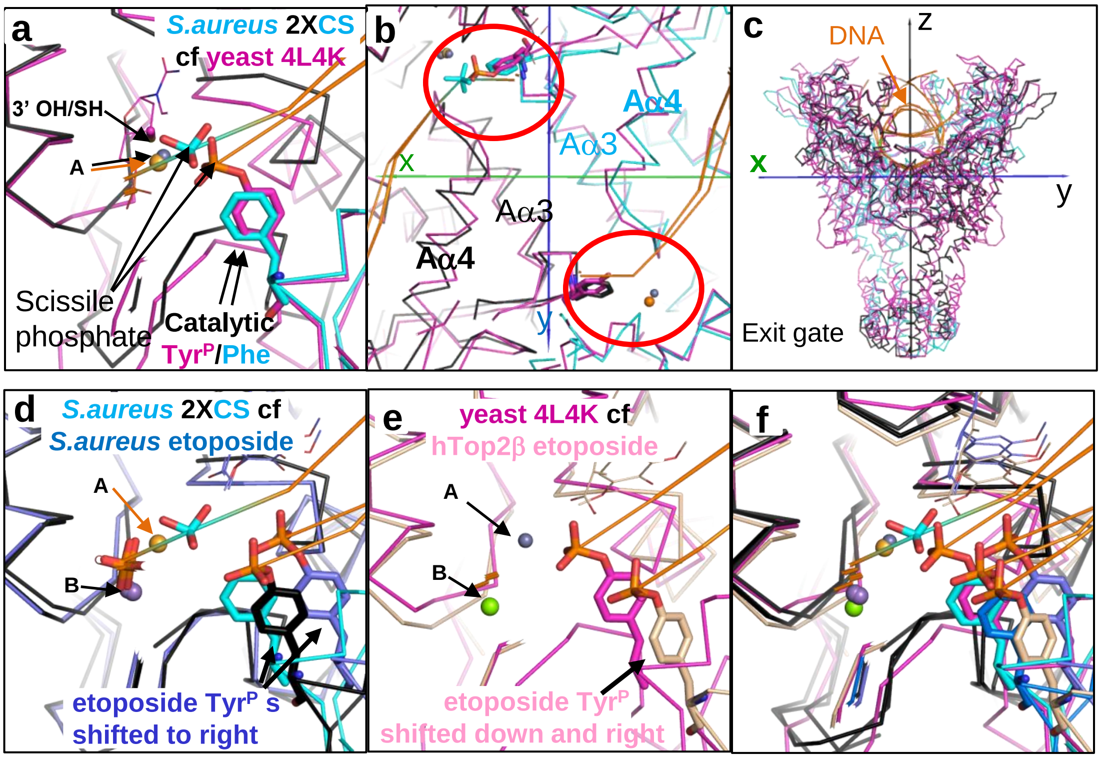

Supplementary Fig. 8. Similarities and differences between procaryotic and eucaryotic topo2A structures.

(a,b,c) Comparisons of 'catalytically-competent' (CRsym type) conformations of a procaryotic and a eucaryotic topo2A. Superposition of *S.aureus* DNA gyrase (2XCS – black/cyan) and yeast topo II structure (4L4K – magenta). A metal ion is present at the catalytic metal binding site (the A or 3' site) in both structures. In the *S.aureus* structure the catalytic tyrosine has been mutated to a phenylalanine (cyan), and the DNA is uncleaved (path of DNA backbone indicated by cyan and orange line passing through scissile phosphate). In the yeast structure the 3' hydroxyl (3' OH/SH) has been replaced with a sulfur to inhibit religation, and the DNA has been cleaved by the catalytic tyrosine (Tyr^P). In our study, a CRsym cluster of five structures were all similar in conformation to 2XCS, a structure proposed to be in a cleavage competent conformation prior to the first cleavage step. b, and c are views at lower resolution down different axis of the same comparison.

(d) Comparison of an asymmetric (Casym) *S. aureus* etoposide cleavage-complex (black/blue) with *S. aureus* 2XCS (black/cyan). Note that because the *S.aureus* etoposide complex is asymmetric, superposing the BA subunit, or the BA' subunit gives different results – both are shown. Note that one of the etoposide catalytic Tyr^Ps is closer to the catalytic (cyan) position than the other. In the etoposide cleavage complex a single metal ion is seen at the non-catalytic B position (where it interacts with the DNA backbone phosphate on the 3' side of the cleavage site, via a water).

(e) Comparison of the human Top2β etoposide complex (light pink) with yeast topoII (4L4K - magenta). Note the downward shift of the catalytic tyrosine in the human Top2β etoposide structure. Metal ion is at the B site in the etoposide complex. The complex is in a different conformation relative to the bacterial etoposide structure.

(**f**) Comparison of all structures in panels d and e.

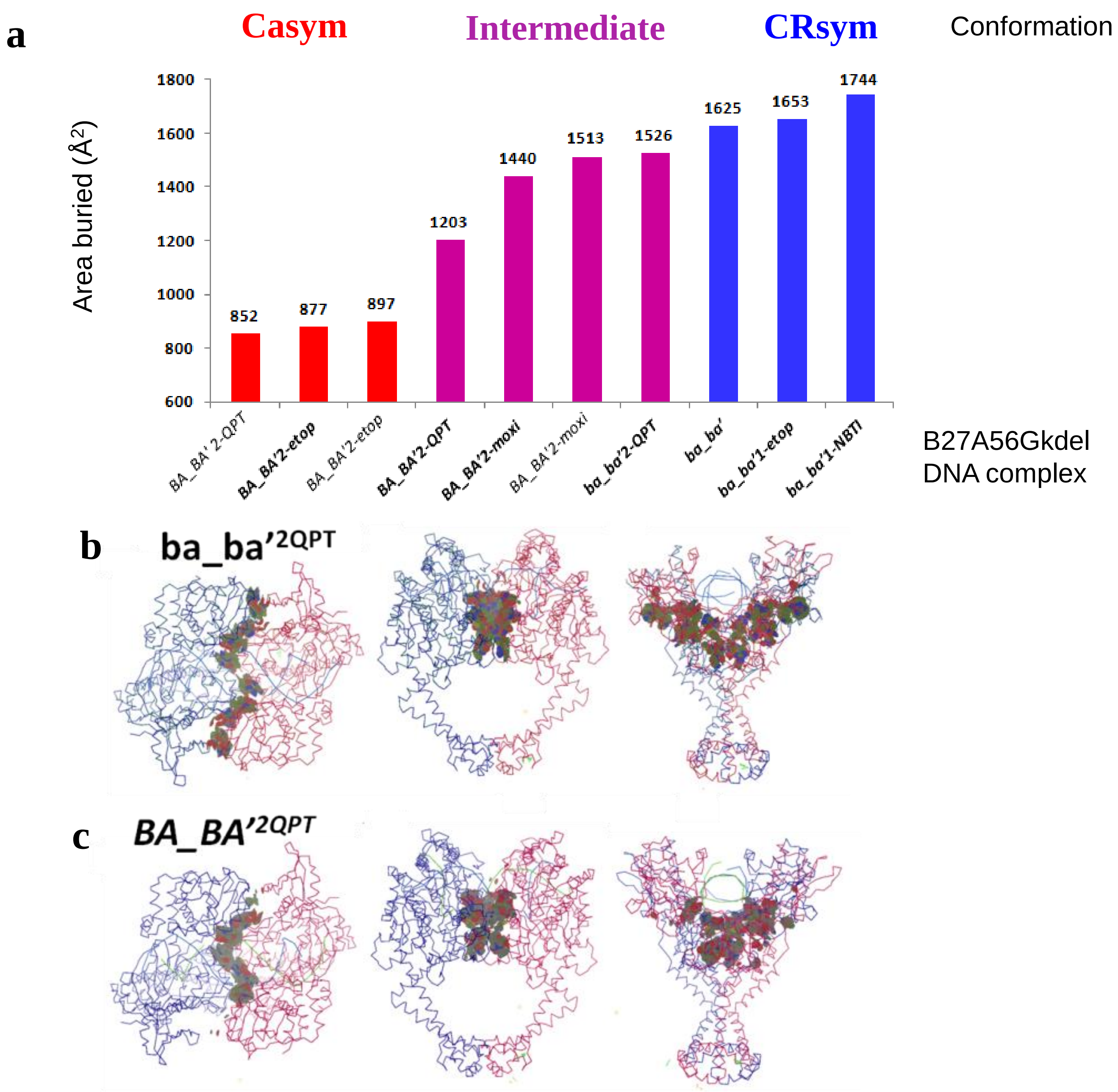

Supplementary Fig. 9. Areas buried between protein subunits at DNA-gate in DNA gyrase complexes. (a) Area buried (Å^2) at DNA-gate in nine *S.aureus* B27A56Gkdel complexes with doubly cleaved or doubly nicked DNA (this study) and one *S.aureus* B27A56Gkdel complex with uncleaved DNA (**ba_ba'$^{1-NBTI}$**) (Bax *et al*., 2010). Note that although the protein-protein interactions between subunits at the DNA-gate are almost identical in the three GSK299423 complexes (Supplementary Fig. 7), the buried area is some 200 Å^2 larger in the two complexes with the greek key domain intact (BA_BA'$^{1-NBTI}$, *BA_BA'$^{1-NBTI}$* from 2XCR; 1927 Å^2 and1959 Å^2) compared with that in which the greek key domain was deleted (ba_ba'$^{1-NBTI}$, 1744 Å^2). In the apo *S.aureus* B27A56 dimer the area buried at the DNA-gate is some 2864 Å^2. All structures compared in panel a have the greek key domain deleted. **(b)** Three orthogonal views of the 1526 Å^2, buried between the two protein subunits at the DNA gate in the **ba_ba'^{2QPT}** complex. **(c)** Three orthogonal views of the 852 Å^2, buried between the two protein subunits, in the ***BA_BA'$^{2-QPT}$*** complex. The areas buried between the two protein subunits at the DNA-gate dimer interface was calculated by Pisa, with all waters removed (see Methods for details). This does not include the interaction between the exit gate domains (which is about the same ~1150 Å^2 in all complexes).

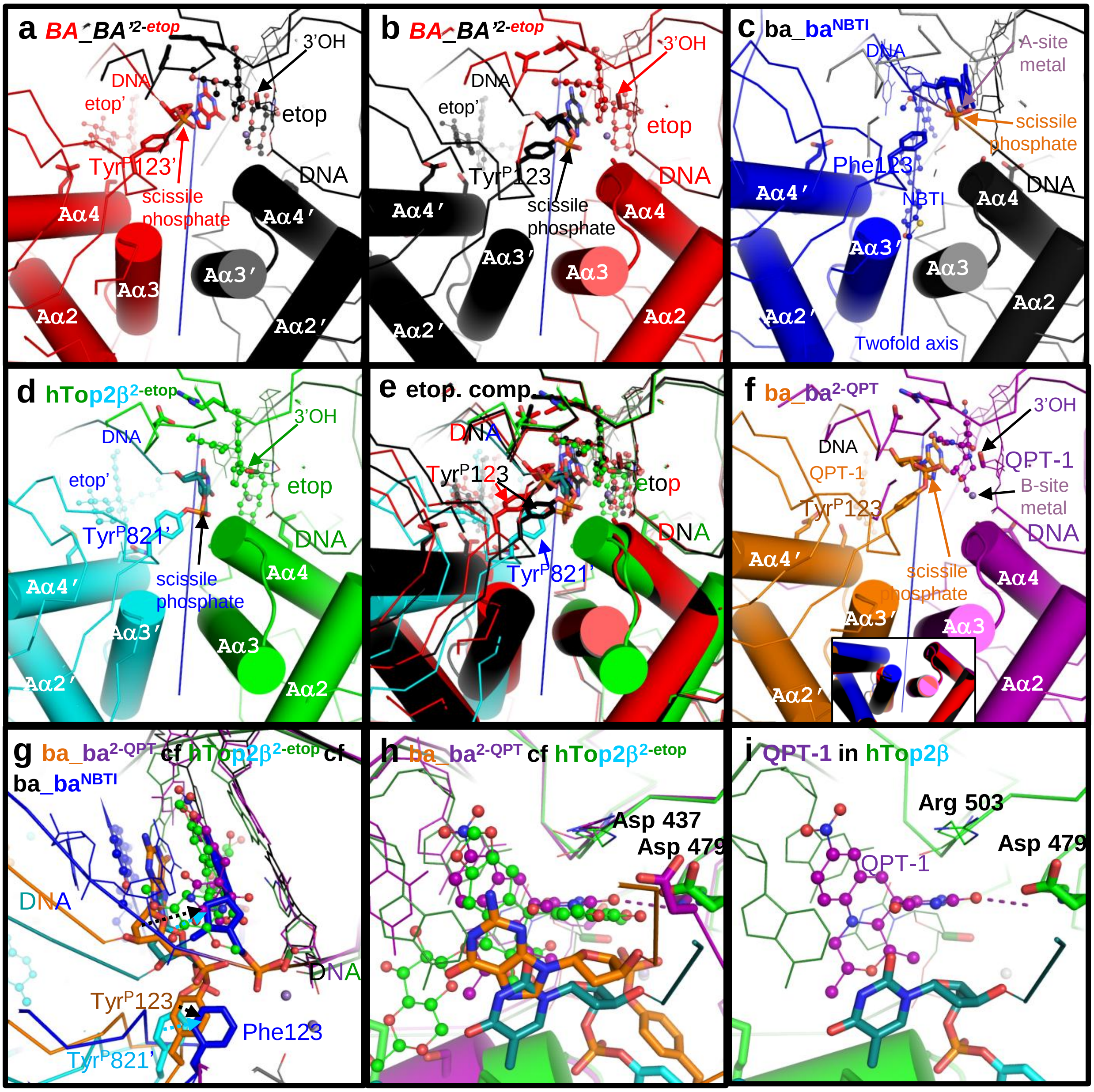

Supplementary Fig. 10. Comparison of bacterial etoposide, NBTI , QPT-1 and human etoposide structures.

(a,b) Views of the two cleavage sites in the ***BA_BA'***$^{2\text{-}etop}$ complex. Note because the ***BA_BA'***$^{2\text{-}etop}$ complex is asymmetric the two cleavage sites are not identical. The three α-helices from the WHDs of the two subunits are shown as cylinders. Compounds are shown in 'ball and stick' view. (**c**) View of a cleavage site in the **ba_ba**^{NBTI} complex (2cxs). Note that the DNA is uncleaved and that the NBTI sits on the twofold axis (blue line). The twofold axis (blue line) relating the two subunits in the ba_ba^{NBTI} complex is also shown in all other panels (a-f). In some other complexes, such as the ***BA_BA'***$^{2\text{-}etop}$ (panels a,b) the two subuinits are related by a 173.5°, rather than by a 180° (twofold) rotation. (**d**) View of a cleavage sites in the hTop2β etoposide complex (3qx3). (**e**) Comparison of three etoposide cleavage sites from panels a, b, and d. Note the different angle between the Aα3 and Aα4 helices in human Top2β versus *S. aureus* gyrase structures correlates with an extra amino acid (see Supplementary Fig. 5). (**f**) View of a cleavage site in the **ba_ba**$^{2\text{-}QPT}$ complex. Inset panel at bottom of figure shows same view with only helices from panels a,b,c and f shown. (**g)** Different view comparing structures of **ba_ba**$^{2\text{-}QPT}$ **cf hTop2β**$^{2\text{-}etop}$ **cf ba_ba**^{NBTI} which shows the different positions of Tyr^{P}123 (*S. aureus* gyrase) and Tyr^{P}821' (human Top2β) – see also Supplementary Fig. 8). (**h,i**) View of a superposition of **ba_ba**$^{2\text{-}QPT}$ and **hTop2β**$^{2\text{-}etop}$ Note in panel **i** QPT-1 is shown in the human Top2β etoposide binding site.

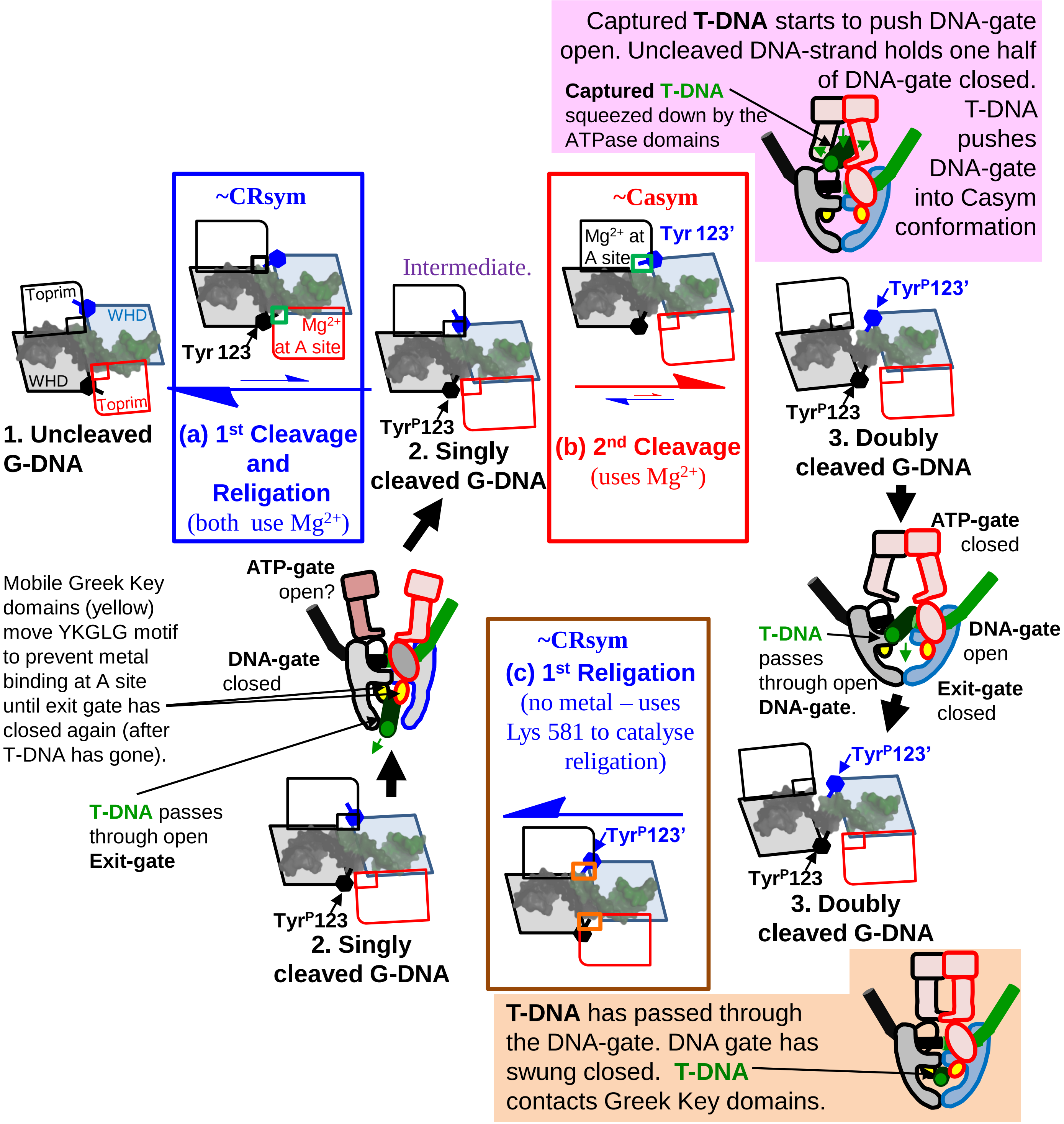

Supplementary Fig.11. Swing-doors mechanism for T-DNA regulation of G-DNA cleavage and religation. Schematic showing the catalytic TOPRIM and WHD domains moving through CRsym (blue box – first DNA cleavage and second DNA religation), Casym (red box – second DNA cleavage) and CRsym (orange box – first DNA religation) conformations to catalyze the two DNA cleavage and religation steps of *S.aureus* DNA gyrase. DNA is shown in green/black space-fill. When the catalytic tyrosine is covalently attached to the DNA, it is labeled (Tyr^{P}123). The TOPRIM domain is shown transparent black or red, with its active site a smaller square in one corner. For DNA cleavage /religation to occur the catalytic tyrosine from the WHD domain of the opposite subunit needs to be correctly aligned with the TOPRIM domain active site (and the substrate DNA). See Supplementary Discussion for details.

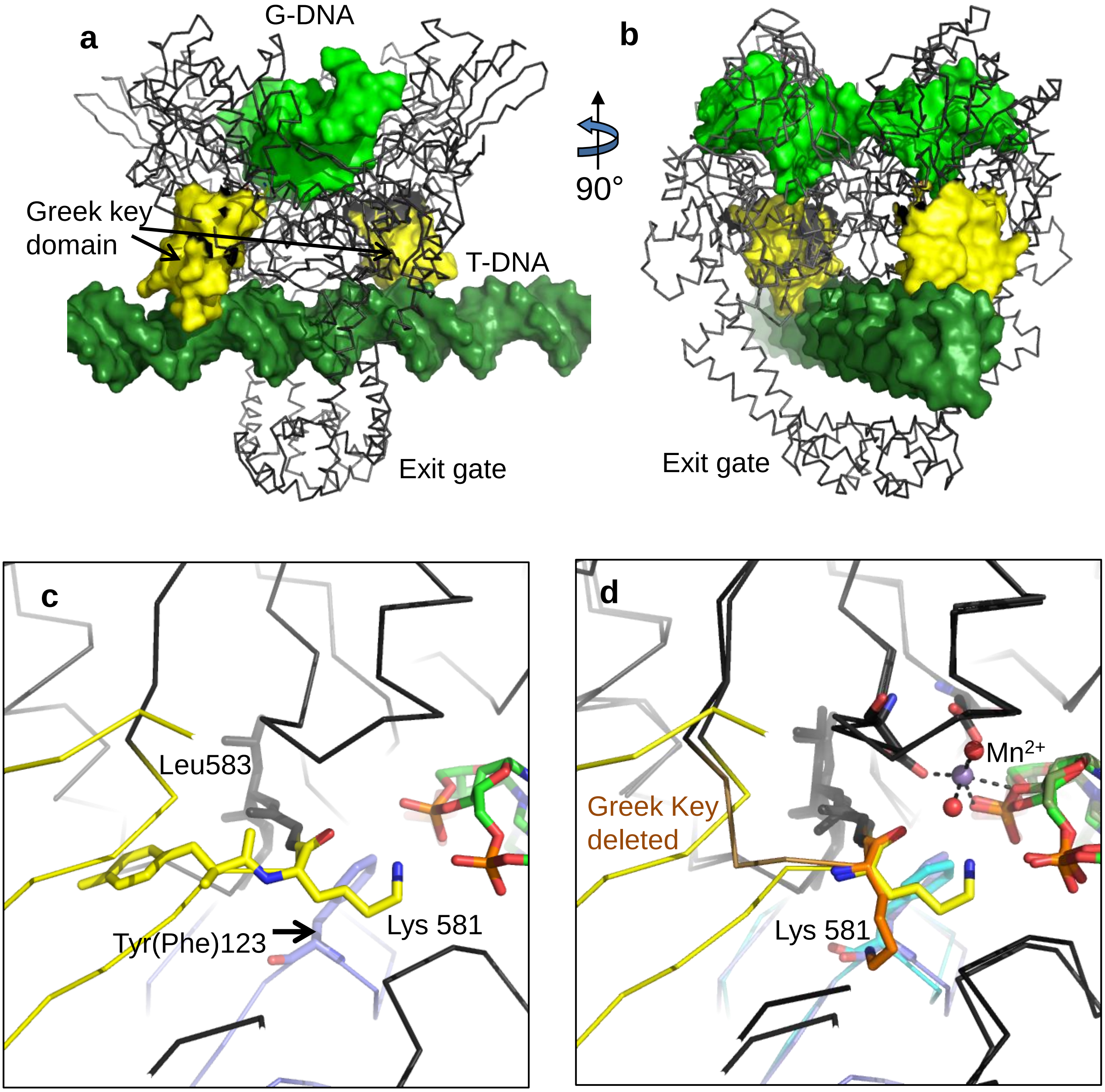

Supplementary Fig. 12. YKGLG motif links Greek key domain to active site in *S. aureus* DNA gyrase.

(**a,b**) Two orthogonal views of a 3.5Å complex of *S.aureus* gyrase with DNA and GSK299423 (2xcr) with a transport DNA (T-DNA) segment modelled in to illustrate how the Greek key domains (yellow) may interact with the transport DNA once it has passed through the DNA gate**.** (**c**) In 2xcr complex no metal ion was observed at the active site, but the side-chain of Lys 581 (yellow carbons) pointed in towards the scissile phosphate. Lys 581 residue is at the C-terminal end of the Greek Key domain (from conserved sequence motif YKGLG). (**d**) In the 2.1Å structure of *S. aureus* gyrase Greek key deletion mutant with DNA and GSK299423 (2xcs – superposed on the 3.5Å structure from panel (c), a metal ion is observed at the catalytic position (A or 3' site) and the side-chain conformation of Lys 581 (orange carbon) is different.

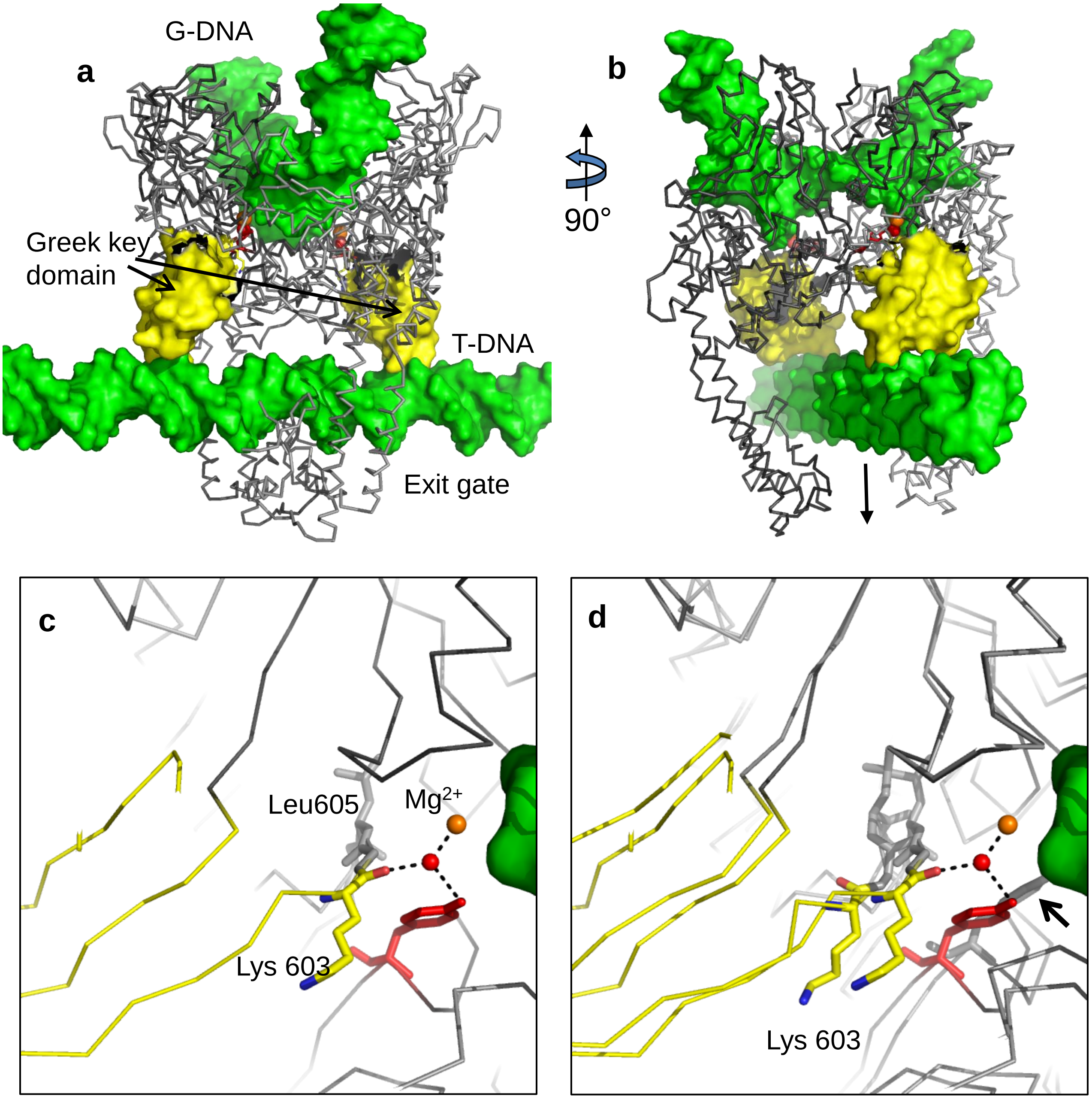

Supplementary Fig. 13. YKGLG motif links the Greek key domain to active site in Yeast topo II.

(**a,b**) Two orthogonal views of a yeast complex with DNA (2rgr) with a transport DNA (T-DNA) segment modelled in to illustrate how the Greek key domains (yellow) may interact with the transport DNA once it has passed through the DNA gate**.** (**c**) In 2RGR complex the metal ion is in the inactive position (the B or Y site) – it is stabilised in this position by interactions through a water with the main chain carbonyl of Lys 603, at the C-terminal end of the Greek key domain (from conserved sequence motif YKGLG). Note in 2RGR complex, the gate-DNA lacks the scissile phosphate and is artificially nicked at the cleavage site. (**d**) In an active conformation of the yeast topoII with DNA (2LK4 - superposed on 2RGR structure from panel (c), the main-chain carbonyl of Lys 603 points away from the metal, and the catalytic tyrosine (arrowed) does not point towards the B (Y) site. In 2LK4 the Greek key domain is in a different position than in 2RGR.

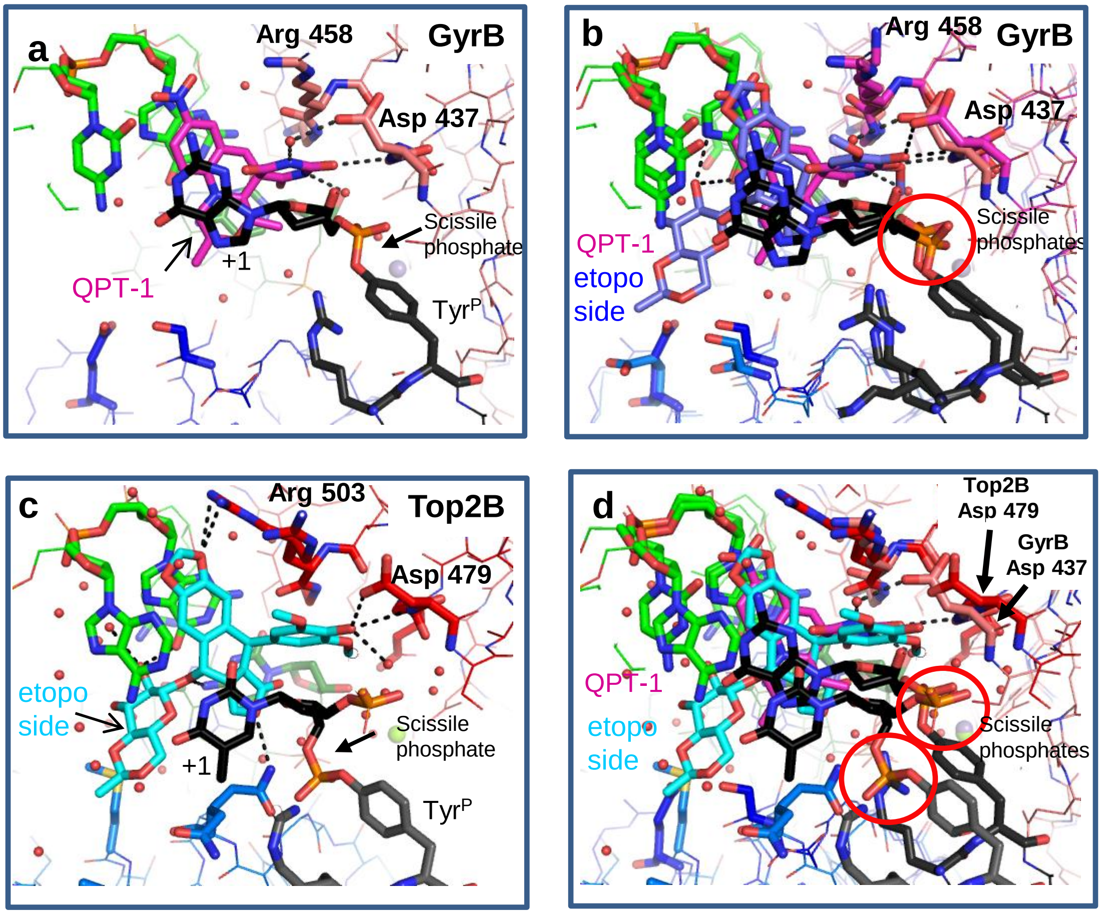

Supplementary Fig. 14. Comparison of the scissile phosphate in human and *S. aureus* topo2A structures to explain QPT-1's bacterial specificity. (**a**) *S. aureus* gyrase QPT-1 structure (2.5 Å). The scissile phosphate is marked and arrowed. QPT-1 in pink carbons. DNA in green and black carbons. Catalytic tyrosine (Tyr^{P}). (**b**) Comparison of the *S. aureus* gyrase structures of QPT-1 (panel a) and etoposide (2.8 Å, Fig. 3c) showing the scissile phosphates are at similar positions (red circle). Etoposide in light blue carbons. **(c**) Human top2β etoposide structure (3QX3)[6]. Etoposide in cyan carbons. (**d**) Comparison of *S. aureus* gyrase QPT-1 (panel a) and human top2β etoposide (panel c) structures showing a dramatic difference in the position of the two scissile phosphates (red circles) and a change in the positions of Asp 437 and Asp 479 residues of *S. aureus* and HuTop2B respectively (black arrows) that may explain the bacterial specificity of QPT-1.

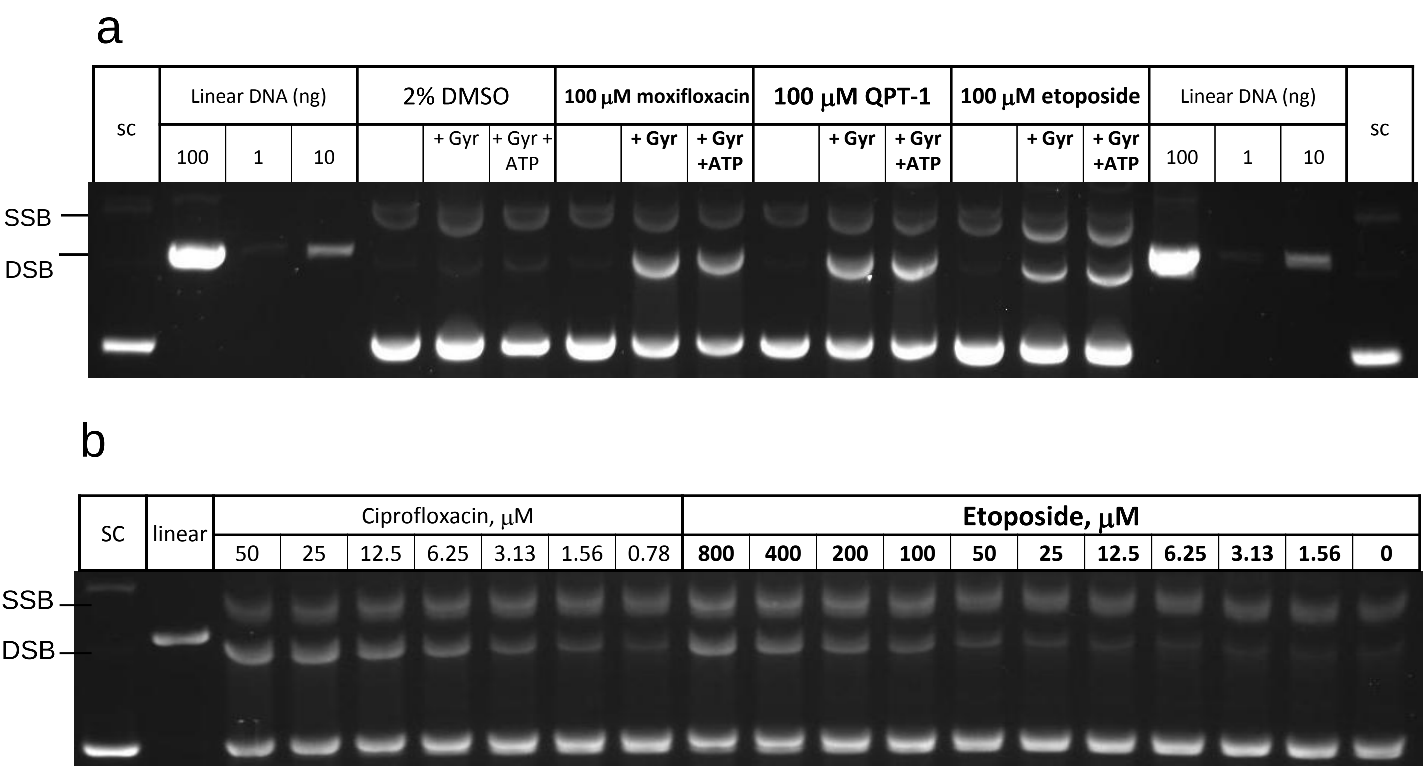

Supplementary Fig. 15. ***S. aureus*** **gyrase DNA cleavage activity with three inhibitors.**
Full scans of the gels used in Figures 2e and 2f respectively: **(a)** Whereas**,** QPT-1 and moxfloxacin induced double-stranded DNA breaks (DSB), etoposide enhanced both single-stranded DNA breaks (SSB) and DSB. Note: all three inhibitors induced DNA gyrase cleavage in an ATP-independent manner. Reactions contained100 ng supercoiled (SC) pBR322 DNA, 50 nM wild -type *S. aureus* gyrase, 100 μM of compound (in 2% DMSO) and 1.5 mM ATP as indicated (see Methods). Linearised pBR322 DNA included as marker. (**b**) A representative gel showing etoposide stabilized both SSB and DSB over a wide concentration range with *S. aureus* gyrase. On this gel, ciprofloxacin is included as the positive control compound.

Synthetic Scheme

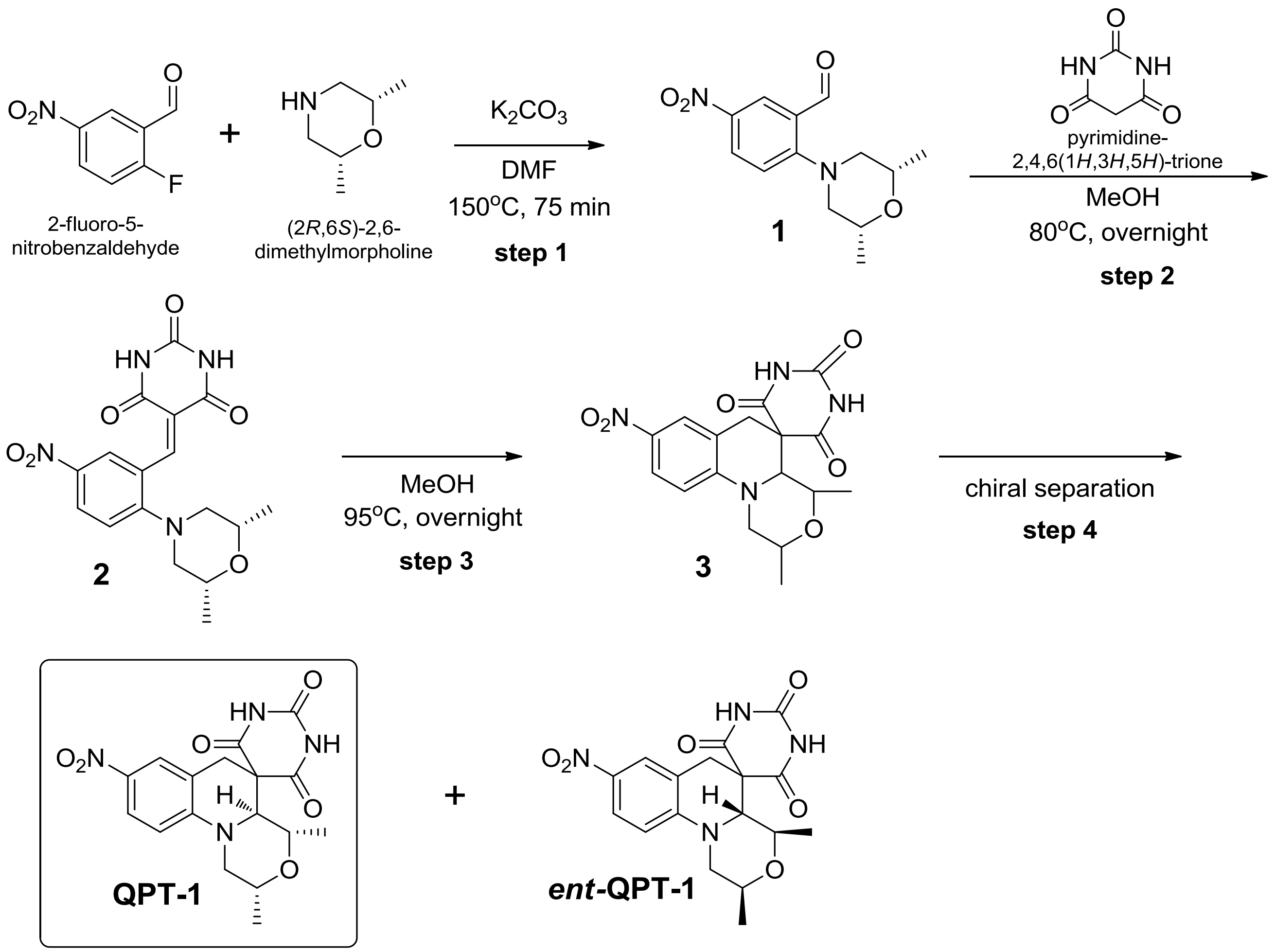

Supplementary Fig. 16. Synthetic scheme for QPT-1

The bioactive (-) enantiomer of QPT-1 is boxed. See Supplementary Methods for details.

SUPPLEMENTARY TABLES

Supplementary Table 1. Structure refinement statistics.

	QPT1-2.5	QPT1-3.15	Etop-2.8	Etop-2.45	Moxi-2.95	Binary
PDB CODE	5CDM	5CDO	5CDN	5CDP	5CDQ	5CDR
Compound	QPT-1	QPT-1	Etoposide	Etoposide	Moxifloxacin	No cmpnd.
Protein	Wild type	Wild type	Wild type	Y123F	Wild type	Y123F
DNA	20-447T	20-447T	20-447	20-12p-8	20-448T	20-12p-8
Space group	$P6_1$	$P2_1$	$P2_1$	$P6_1$	$P2_1$	$P6_1$
Cell dimensions	a = 93.9<br>b = 93.9<br>c = 412.5<br>90,90,120	a = 90.5<br>b = 170.2<br>c = 124.6<br>90,102.8,90	a = 89.8<br>b = 170.1<br>c = 124.5<br>90,102.3,90	a = 93.4<br>b = 93.4<br>c = 411.2<br>90,90,120	a = 87.9<br>b = 170.6<br>c = 125.7<br>90,103.3,90	a=93.4<br>b=93.4<br>c=410.6<br>90,90,120
Resolution (Å)	2.50	3.15	2.80	2.45	2.95	2.65
No. of complexes in asym. unit	1	2	2	1	2	1
Names. of cmplxes in asym. unit	ba_ba'^2-QPT	BA_BA'^2-QPT<br>*BA_BA'* ^2-QPT	BA_BA'^2-etop<br>*BA_BA'* ^2-etop	ba_ba'^1-etop	BA_BA' ^2-moxi<br>*BA_BA'* ^2-moxi	ba_ba'
**Refinement***						
Resolution (Å)	36.8-2.50<br>(2.54-2.50)	58.4-3.15<br>(3.21-3.15)	40.0-2.80<br>(2.86-2.80)	20.0-2.45<br>(2.51-2.45)	20.0-2.95<br>(3.03-2.95)	39.7-2.65<br>(2.72-2.65)
No. reflections	67361 (3394)	62307 (3362)	87157 (5951)	70923 (4932)	74584 (4890)	58050<br>(4310)
$R_{work/}$ R_{free} (%)	16.3/19.2<br>(27.8/31.2)	21.5/24.6<br>(29.6/33.4)	21.8/24.4<br>(24.4/26.5)	17.7/21.1<br>(21.8/24.9)	17.5/21.8<br>(23.2/27.2)	18.8/21.0<br>(22.3/25.4)
No. Atoms[a]						
Protein	11892	23318	20825	10998	20975	11001
DNA	783	1605	1585	829	1564	753
Ligand/ion	74	162	196	48	258	39
Water	327	82	361	770	276	644
B-factors						
Protein	51.2	47.4	76.6	37.3	92.0	48.9
DNA	46.9	41.0	65.2	43.2	85.3	51.6
Ligand/ion	41.2	30.7	67.8	46.3	76.6	52.0
Water	41.5	22.3	54.1	38.7	63.3	45.1
R.m.s deviations						
Bond lengths (Å)	0.010	0.005	0.008	0.008	0.009	0.008
Bond angles (°)	1.135	0.96	0.99	1.03	1.04	0.98

Each dataset was collected from a single frozen crystal at the ESRF or the DLS (see accompanying paper, [2] for details on crystallisation and data collection, and sequences of 20mer DNA homoduplexes).
*Highest resolution shell is shown in parenthesis. [a] The number of atoms does not include hydrogens and B-factors do not include hydrogens.

Supplementary Table 2. Tautomers of QPT-1 evaluated in docking experiments.

Taut No.	Neutral tautomer	Equivalent tautomer with central OH of barbituric acid O⁻. Or picture	Comments
1	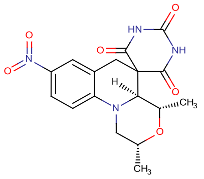		Tautomer 1. The barbituric acid is a pyrimidine trione in tautomer 1, but not in other tautomeric forms of QPT-1. **Tautomer 1 was used at both sites in the 2.5Å ba_ba'$^{2\text{-}QPT}$ complex.**
2	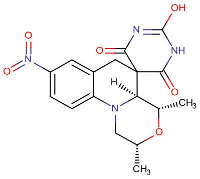	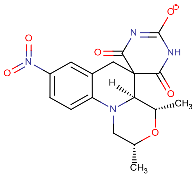	Tautomers 2 and 3 are diasteromers.
3	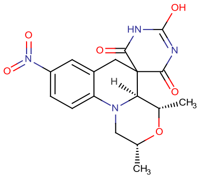	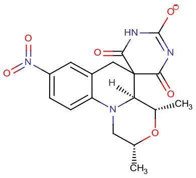	Tautomers 2 and 3 are diasteromers.
4	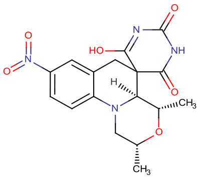		Tautomers 4 and 5 are diasteromers. **Tautomer 4 was used at one site in the 3.15Å *BA_BA'*$^{2\text{-}QPT}$ complex.**
5	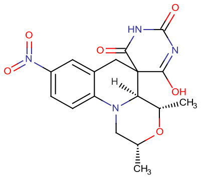		Tautomers 4 and 5 are diasteromers. **Tautomer 5 was used at one site in the 3.15Å BA_BA'$^{2\text{-}QPT}$ complex.**
6	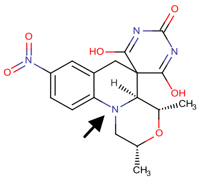	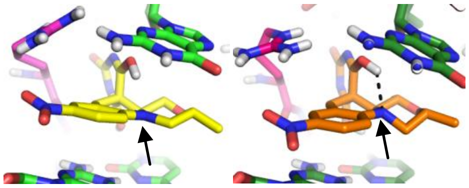	**Tautomer 6 was used at one site in the 3.15Å BA_BA'$^{2\text{-}QPT}$ complex (central nitrogen sp^2 -yellow). Tautomer 6 was used at one site in the 3.15Å *BA_BA'*$^{2\text{-}QPT}$ complex (central nitrogen sp^3 -orange).**
7	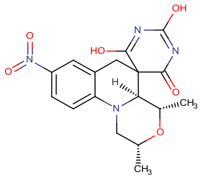	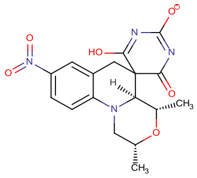	Tautomers 7 and 8 are diasteromers.
8	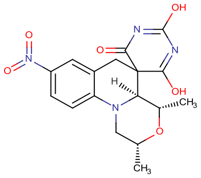	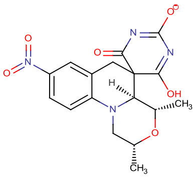	Tautomers 7 and 8 are diasteromers.

Note: The nitrogen (arrowed in tautomer number 6) shared between the central and the dimethyl morpholine rings was modelled as **sp^3** in only one of of six QPT-1 binding sites. Note that the carbon shared between the central ring and the barbituric acid ring becomes chiral if the barbituric acid tautomer is not symmetric.

Supplementary Table 3. Target potencies of QPT-1, etoposide and moxifloxacin in topoisomerase enzyme assays.

Enzyme	Reaction	Compound IC_{50} or CC_{50} (µM)[a]		
		QPT-1	Etoposide	Moxifloxacin
**IC_{50}**				
E.coli DNA gyrase	supercoiling	0.65	134 [c]	1.1 [h]
E.coli Topo IV	decatanation	30 [g]	107 [d]	0.6 [h]
S.aureus DNA gyrase[b]	supercoiling	-	48 [e]	17 [h]
S.aureus Topo IV	decatanation	-	246 [f]	3.8 [h]
Human Top2α	relaxation	>500	45	>2000 [i]
**Cleaved complex CC_{50}**				
E.coli DNA gyrase	DNA cleavage	8.6	113	0.6 [h]
E.coli Topo IV	DNA cleavage	-	103	0.5 [h]
S.aureus DNA gyrase[b]	DNA cleavage	8.8	107	2.5 [h]
S.aureus Topo IV	DNA cleavage	-	126	0.8 [h]
Human Top2α	DNA cleavage	>800	21	>800

[a] IC_{50} is the amount of compound required to inhibit 50 % of the topoisomerase catalytic activity. Cleaved-complex CC_{50} is defined as amount of compound that causes half-maximal induction of linear DNA (double-stranded DNA breaks). Values are the average of at least two independent experiments.

[b] Full length, wild-type form of *S. aureus* DNA gyrase protein.

[c, d, e, f] Fitted IC_{50}s curves plateaued at approx. 60, 75, 75 and 50 % of maximal inhibition, respectively.

[g] Data from [3]

[h] All enzyme data for moxifloxacin taken from [4] except [i]

[i] Human top2α taken from [5]

- not tested

Supplementary Table 4. Antibacterial spectrum of QPT-1, etoposide and teniposide.

**Bacterial strain**	**MIC[a] (μg/ml)**		
	QPT-1	Etoposide	Teniposide
**Gram-positive**			
Staphylococcus aureus Oxford	0.25	32	8
Staphylococcus aureus WCUH29	0.25	32	8
Streptococcus pneumoniae 1629	2	1.5	1
Streptococcus pneumoniae ERY2	4	4	0.25
Streptococcus pyogenes 1307006P	4	8	8
Streptococcus pyogenes 1308007P	4	8	8
Enterococcus faecalis I	4	64	32
**Gram-negative**			
Escherichia coli 7623	8	>256	>128
Escherichia coli 7623 Δ*tolC*	0.063	0.75	0.5
Haemophilus influenzae H128	≤0.125	128	16
Haemophilus influenzae H128 Δ*acrB*	≤0.125	1	1
Moraxella catarrhalis 1502	0.25	256	32
Klebsiella pneumoniae 1161486	> 32	>256	>128
Klebsiella pneumoniae 1161486 Δ*tolC*	0.125	48	64
Pseudomonas aeruginosa PAO1	>32	>256	>128
Pseudomonas aeruginosa PAO322 Δ(*mexAB-oprM*) Δ(*mexCD-oprJ*) Δ(*mexEF-oprN*)	0.5	48	96
Acinetobacter baumannii BM4454	16[b]	>256	>128
Acinetobacter baumannii BM4652 Δ*adeABC* Δ*adeIJK*	0.125[c]	48	32

[a] MIC values are the averages of two independent determinations

[b] *A. baumannii* ACM1010 wild-type

[c] *A. baumannii* ACM1015 ΔadeABC ΔadeIJK

Supplementary Table 5. Antimicrobial susceptibility of quinolone-resistant *Streptococcus pneumoniae* strains to etoposide.

Streptococcus pneumoniae	MIC[a] (μg/ml)	
	**Etoposide**	**Moxifloxacin**
**Isogenic strain:**		
R6 wild-type	4	0.063
R6 ParC S79F	4	0.125
R6 ParC S79Y	16	0.5
R6 ParC D83N	8	2
R6 ParC D83Y	2 / 1	2
R6 GyrA S81F	1 / 0.5	0.25
R6 GyrA S81Y	1	0.5
R6 ParC S79Y, GyrA S81Y	4	8
R6 ParC S79Y, GyrA E85K	8	8
**Clinical strain:**		
925118 ParC S79F, GyrB E474K	8	0.25
205118 ParC S79Y, K137N, GyrB E474K	8	0.5
5303 ParC S79Y, GyrB D435N	16	2

[a] MIC values are from two independent determinations.

Supplementary Table 6. Antimicrobial susceptibility of etoposide-resistant *S. pneumoniae* to other topo2A inhibitors.

Bacterial strain	MIC[a] (μg/ml)									
	Etop-oside	Moxifl-oxacin	Novo-biocin	GSK299423 (NBTI)	Tenip-oside	Doxo-rubicin	Dauno rubicin	Mitox antro ne	Ams acrin e	Ellipti-cine
S. pneumoniae										
1629 parent	2	0.031	1	0.016	2	0.063	0.125	4	64	1
1629 GyrB R447C	16	0.094	0.125	0.031	4	0.031	0.063	2	32	0.5
1629 GyrB ΔL407 - S410	16	0.047	0.5	0.006	8	0.031	0.094	1.5	48	0.5
1629 GyrB Q594P	16	0.063	0.25	0.008	4	0.063	0.063	2	32	0.5
1629 GyrB L412V	8	0.063	2	0.016	2	0.063	0.125	2	64	0.5
1629 GyrB I522S	8	0.063	0.5	0.031	8	0.031	0.063	2	32	0.5
1629 GyrA E14D	8	0.063	0.5	0.016	4	0.063	0.063	2	64	0.5
1629 GyrA K40T[b]	4	0.031	$\leq$0.031	0.016	4	0.016	0.016	0.25	2	0.125
1629 GyrA R44C[b]	8	0.031	0.125	0.008	4	0.063	0.063	1	32	0.5
1629 GyrA E52A	8	0.063	2	0.031	4	0.063	0.125	2	64	1
1629 GyrA I83S[d]	8	0.063	0.25	0.031	4	0.063	0.063	1	16	0.25

[a]MIC values are averages of two independent determinations. Significant ($\geq$ 4-fold) resistance and hypersensitivity are highlighted in red and green, respectively.

[b]The mutations also result in slower growth of bacterial cells.

Supplementary Table 7. Sequence conservation at the DNA gate.

	% of amino acid residues conserved at DNA gate	
**a. Protein:DNA interactions**	**Procaryotes**	**Eucaryotes**
Procaryotes	54% (19/35)	49% (17/35)
Eucaryotes		83% (29/35)
**b. Protein:protein interactions**	**Procaryotes**	**Eucaryotes**
Procaryotes	50% (12/24)	25% (6/24)
Eucaryotes		58% (14/24)

Conservation of amino-acids at the DNA gate between procayrotic and eucaryotic topo2As. Absolutely conserved amino-acid residues involved in (**a**) protein:DNA interactions and (**b**) protein:protein interactions at the DNA gate, between the five procaryotic and three eucaryotic sequences shown in Supplementary Fig. 5.

In the table residues are counted as contacting the DNA (or protein) if they are contact residues in at least one of the three eucaryotic structures <u>and</u> at least one of the five *S. aureus* structures, whose contacts are mapped onto five different bacterial sequences in Supplementary Fig. 5 (*S. aureus* Gyrase, *M. tuberculosis* Gyrase, *E.coli* Gyrase, *S. pneumonia*e Topo IV and *A. baumanii* Topo IV).

Catalytic RY residues are not included in the contact sets.

Supplementary Table 8. RMS fits of catalytic domains from twelve *S.aureus* DNA gyrase complexes with DNA (and compounds).

PDB ID<br>ESD- Å	2xcs<br>0.17	5cdq<br>0.41		5cdm<br>0.30	5cdo<br>0.43		5cdn<br>0.40		5cdp<br>0.28	5cdr<br>0.33	2xcr<br>0.47	2xcq<br>0.42
Reso.<br>Cmpnd.<br>DNA<br>Name	2.1 Å<br>NBTI<br>Intact<br>ba_ba’	2.95Å<br>Moxi<br>Cleavd<br>BA_BA’	2.95Å<br>Moxi<br>Cleavd<br>*BA_BA'*	2.5 Å<br>QPT-1<br>Cleavd<br>ba_ba’	3.15 Å<br>QPT-1<br>Cleavd<br>BA_BA’	3.15 Å<br>QPT-1<br>Cleavd<br>*BA_BA'*	2.8Å<br>ETOP<br>Cleavd<br>BA_BA’	2.8 Å<br>ETOP<br>Cleavd<br>*BA_BA'*	2.45 Å<br>ETOP<br>Nicked<br>ba_ba’	2.65Å<br>-<br>Nicked<br>ba_ba’	3.5Å<br>NBTI<br>Intact<br>BA_BA’(*BA_BA’*)	2.98Å<br>-<br>-<br>BA_BA
2.1 Å<br>NBTI<br>ba_ba’	[0.150]<br>179.9°<br>[0.188]	0.284<br>0.978<br>[0.992]	0.278<br>1.06<br>[1.082]	0.418<br>1.129<br>[1.135]	0.346<br>1.488<br>[1.484]	0.420<br>1.807<br>[1.823]	0.480<br>1.624<br>[1.631]	0.463<br>1.685<br>[1.702]	0.205<br>0.268<br>[0.325]	0.270<br>0.498<br>[0.540]	0.409 (0.420)<br>0.514 (0.481)<br>[0.522] (0.488)	2.529<br>3.480<br>[3.480]
2.95Å<br>Moxi<br>BA_BA’		[0.315]<br>179.0°<br>[0.467]	0.096<br>0.282<br>[0.656]	0.407<br>0.731<br>[0.746]	0.288<br>0.844<br>[0.852]	0.319<br>1.259<br>[1.429]	0.360<br>1.117<br>[1.237]	0.353<br>1.209<br>[1.363]	0.253<br>0.824<br>[0.876]	0.269<br>0.717<br>[0.781]	NC	NC
2.95Å<br>Moxi<br>*BA_BA'*			0.365<br>177.5°<br>[0.822]	0.430<br>0.825<br>[0.852]	0.323<br>0.840<br>[0.841]	0.330<br>1.110<br>[1.483]	0.395<br>0.999<br>[1.271]	0.370<br>1.063<br>[1.408]	0.257<br>0.901<br>[0.985]	0.272<br>0.770<br>[0.881]	NC	NC
2.5 Å<br>QPT-1<br>ba_ba’				0.248<br>179.6°<br>[0.292]	0.334<br>0.902<br>[0.901]	0.397<br>1.202<br>[1.180]	0.381<br>1.065<br>[1.029]	0.402<br>1.152<br>[1.133]	0.298<br>0.953<br>[0.971]	0.284<br>0.738<br>[0.773]	NC	NC
3.15 Å<br>QPT-1<br>BA_BA’					0.267<br>179.9°<br>[0.272]	0.318<br>0.916<br>[0.944]	0.316<br>0.803<br>[0.831]	0.364<br>0.892<br>[0.931]	0.266<br>1.301<br>[1.296]	0.296<br>1.133<br>[1.121]	NC	NC
3.15 Å<br>QPT-1<br>*BA_BA'*						0.378<br>172.9°<br>[1.454]	0.252<br>0.352<br>[1.332]	0.180<br>0.289<br>[1.409]	0.345<br>1.646<br>[1.678]	0.309<br>1.430<br>[1.465]	NC	NC
2.8 Å<br>ETOP<br>BA_BA’							[0.379]<br>174.1°<br>[1.190]	0.164<br>0.222<br>[1.248]	0.372<br>1.466<br>[1.475]	0.344<br>1.248<br>[1.248]	NC	NC
2.8 Å<br>ETOP<br>*BA_BA'*								0.306<br>173.5°<br>[1.320]	0.367<br>1.526<br>[1.555]	0.341<br>1.315<br>[1.353]	NC	NC
2.45 Å<br>ETOP<br>ba_ba’									0.159<br>179.8°<br>[0.293]	0.159<br>0.328<br>[0.440]	NC	NC
2.65Å<br>-<br>ba_ba’										0.158<br>179.9°<br>[0.369]	NC	NC
3.5Å<br>*NBTI*<br>BA_BA’											0.352<br>179.4°<br>[0.374]	NC
2.98Å<br>-<br>BA												0.0<br>180.0<br>[0.0]

PDB codes and estimated coordinate errors (ESD) are at top of the table. The resolution (Reso.), compound (Cmpnd.), and state of the DNA (DNA) is given. The DNA is: cleaved (at both sites), intact (catalytic Tyr mutated to Phe), or nicked (artificial gaps in DNA at both cleavage sites). The names (Name) of the complexes being compared are given as: ba_ba’, BA_BA’, *BA_BA’*, or BA_BA (the later is for the apo dimer which sits on a crystallograhic twofold and therefore has two equivalent subunits). Structures are highlighted as in Supplementary Fig. 7: CRsym (blue), Casym (red) and int. (purple).

Numbers in cells on table diagonal give: (i) internal RMS fit of 68 ca atoms (34 from the GyrB TOPRIM domain and 34 from the GyrA WHD) of one covalently fused subunit (ba,BA,*BA*) onto the other subunit (ba’,BA’,*BA’*) in the dimer (ii) angle to rotate the ba’/BA’/*BA’* subunit onto the ba/BA/*BA* subunit (iii) RMS fit of 136 ca atoms [BA’_BA] onto [BA_BA’].

In the main part of the table: (i) the first number is the RMS (root mean square) fit of the 68 ca atoms of the ba subunit from first complex - onto the ba subunit of the second. (ii) The second number is the RMS fit of the 136 (68+68) ca atoms from both subunits of the first complex (ba_ba’) onto the same 136 ca atoms of the second complex (ba_ba’). (iii) The third number [in square brackets] is for the fit of ba_ba’ onto ba’_ba. For fits where one or both of the complexes being compared are nearly C2 symmetric, the fit of ba_ba’ onto ba_ba’, and the fit of ba_ba’ onto ba’_ba give very similar results. The two complexes in the 3.5Å NBTI structure are similar, and only comparisons with the 2.1Å were calculated. NC = not calculated.

Supplementary Table 9A. Docking eight QPT-1 tautomers into two binding sites in 2.5Å ba_ba'$^{2\text{-}QPT}$ complex.

Tautomer number	Docking Parameter	SITE 1 – next to **b** GyrB subunit			SITE 2 – next to **b'** GyrB subunit		
		Forrcefield for nitrogen in central ring.			Forrcefield for nitrogen in central ring.		
		SP2	SP3 chiral α	SP3 chiral αα	SP2	SP3 chiral α	SP3 chiral αα
1	RSCC	**0.660**	0.654	0.656	**0.625**	0.622	0.625
	Local Strain	**0.98**	4.31	2.69	**3.33**	4.80	3.59
	Chemscore	**-3.77**	-3.78	-4.05	**6.31**	4.20	4.06
	PLP	**-61.2**	-62.9	-62,7	**-42.8**	-45.5	-45.8
2	RSCC	0.658	0.636	0.656	0.642	0.637	0.635
	Local Strain	4.17	5.24	3.78	4.46	5.42	4.47
	Chemscore	-5.58	-5.78	-5.56	0.61	0.76	0.11
	PLP	-66.2	-66.5	-64.3	-45.1	-45.4	-45.2
3	RSCC	0.657	0.638	0.641	0.626	0.621	0.621
	Local Strain	0.84	4.11	3.97	4.64	5.04	4.42
	Chemscore	-5.39	-4.95	-5.86	3.59	2.38	3.64
	PLP	-56.4	-57.3	-57.6	-38.1	-38.5	-38.3
4	RSCC	0.678	0.653	0.638	0.684	0.62	0.668
	Local Strain	1.15	4.79	0.94	2.62	5.91	1.88
	Chemscore	-6.86	-4.90	-4.52	-2.45	0.199	1.47
	PLP	-68.3	-65.1	-63.5	-57.1	-45.8	-54.9
5	RSCC	0.663	0.652	0.651	0.643	0.645	0.653
	Local Strain	5.13	5.19	5.12	4.66	4.97	5.69
	Chemscore	-6.00	-6.65	-6.62	1.15	1.12	-0.37
	PLP	-63.2	-63.3	-63.2	-42.7	-43.55	-43.5
6	RSCC	0.664	0.654	0.653	0.641	0.652	0.653
	Local Strain	5.35	5.93	5.87	5.06	6.56	6.63
	Chemscore	-7.56	-7.93	-7.87	-2.96	-3.10	-3.25
	PLP	-67.0	-67.3	-67.3	-45.3	-45.5	-45.7
7	RSCC	0.660	0.641	0.655	0.638	0.636	0.624
	Local Strain	4.91	5.47	5.21	4.89	5.02	6.00
	Chemscore	-6.40	-8.00	-7.91	-1.52	-1.69	-1.37
	PLP	-61.9	-63.0	-62.2	-40.4	-41.1	-42.1
8	RSCC	0.633	0.633	0.633	0.645	0.639	0.641
	Local Strain	5.37	5.72	5.72	5.16	5.42	5.47
	Chemscore	-8.15	-8.48	-8.17	-2.54	-4.03	-3.74
	PLP	-65.7	-66.7	-66.4	-45.2	-45.3	-45.71

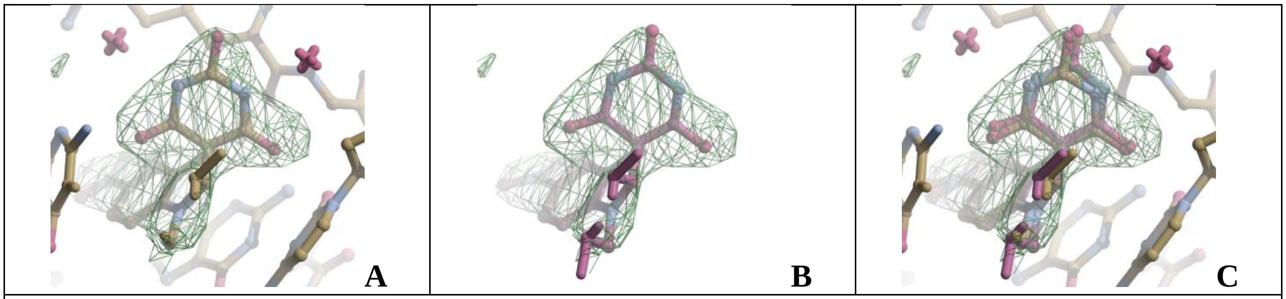

Note the differences in coordinates between different tautomers are small (e.g. refined coordinates of (A) tautomer 3 and (B) tautomer 4 as fit by Afitt with 3.5 sigma Fo-Fc density from diffence map shown. In (C) the structures are superposed). Note also in Afitt docked structures the pucker on the dimethylmorpholino ring was not always correct.

Supplementary Table 9B. Docking eight QPT-1 tautomers into two binding sites in 3.15Å BA_BA'$^{2\text{-QPT}}$ complex.

Tautomer number	Docking Parameter	SITE 1 – next to B GyrB subunit			SITE 2 – next to B' GyrB subunit		
		Forrcefield for nitrogen in central ring.			Forrcefield for nitrogen in central ring.		
		SP2	SP3 chiral α	SP3 chiral αα	SP2	SP3 chiral α	SP3 chiral αα
1	RSCC	0.532	0.530	0.533	0.465	0.489	0.493
	Local Strain	2.40	2.55	1.19	1.31	2.41	0.67
	Chemscore	4.42	4.76	5.04	4.54	2.78	2.85
	PLP	-39.5	-37.8	-38.0	-40.3	-45.7	-45.1
2	RSCC	0.518	0.467	0.463	0.415	0.511	0.478
	Local Strain	2.71	0.567	0.54	1.10	2.85	1.75
	Chemscore	1.56	9.80	9.63	10.5	-1.61	-0.14
	PLP	-37.9	-12.43	-12.8	-16.3	-49.7	-48.4
3	RSCC	0.578	0.481	0.556	0.437	0.494	0.430
	Local Strain	0.09	2.67	2.18	1.87	2.52	0.88
	Chemscore	-1.25	4.47	4.38	3.79	0.27	4.66
	PLP	-43.7	-31.6	-32.0	-33.0	-56.2	-30.8
4	RSCC	0.625	0.548	0.545	0.578	0.500	0.576
	Local Strain	0.187	3.16	0.237	0.43	2.17	0.56
	Chemscore	-3.59	2.06	-2.28	-3.67	-1.50	-1.29
	PLP	-54.6	-42.1	-52.4	-60.3	-55.1	-59.9
5	RSCC	0.514	0.496	0.497	**0.645**	0.618	0.653
	Local Strain	4.10	3.37	3.63	**0.346**	1.45	0.415
	Chemscore	1.30	0.67	0.48	**-3.02**	-2.12	-2.02
	PLP	-46.2	-45.38	-45.6	**-59.6**	-55.34	-56.2
6	RSCC	**0.684**	0.556	0.676	0.568	0.556	0.570
	Local Strain	**0.295**	4.57	0.225	0.58	2.36	0.64
	Chemscore	**-6.37**	-0.78	-6.10	-5.78	-5.04	-4.69
	PLP	**-59.89**	-46.7	-58.0	-62.4	-59.8	-61.48
7	RSCC	0.486	0.587	0.481	0.558	0.478	0.529
	Local Strain	0.26	-0.84	0.17	0.36	2.54	0.43
	Chemscore	-4.58	-0.83	-2.72	-3.27	-3.56	-1.67
	PLP	-47.9	-37.8	-47.5	-50.7	-49.5	-49.1
8	RSCC	0.523	0.502	0.504	0.603	0.575	0.570
	Local Strain	4.35	3.78	3.75	0.47	2.56	0.47
	Chemscore	-1.15	-0.33	-0.25	-5.77	-5.16	-5.00
	PLP	-45.2	-42.83	-42.8	-59.9	-57.8	-58.8

Red highlights the tautomers used in the model.

Supplementary Table 9C. Docking eight QPT-1 tautomers into two binding sites in 3.15Å *BA_BA*'$^{2\text{-QPT}}$ complex.

Tautomer number	Docking Parameter	SITE 1 – next to *B* GyrB subunit			SITE 2 – next to *B'* GyrB subunit		
		Forrcefield for nitrogen in central ring.			Forrcefield for nitrogen in central ring.		
		SP2	SP3 chiral α	SP3 chiral αα	SP2	SP3 chiral α	SP3 chiral αα
1	RSCC	0.509	0.526	0.527	0.570	0.518	0.560
	Local Strain	4.32	4.18	2.75	-2.79	4.03	0.856
	Chemscore	-3.62	-3.37	-3.37	0.47	6.93	3.41
	PLP	-44.80	-43.2	-43.3	-40.99	-30.34	-39.6
2	RSCC	0.542	0.570	0.566	0.519	0.464	0.521
	Local Strain	4.39	4.69	4.82	-2.55	4.07	0.654
	Chemscore	-5.85	-5.19	-5.13	-0.35	4.45	0.12
	PLP	-50.11	-47.3	-47.3	-40.12	-32.56	-39.8
3	RSCC	0.517	0.456	0.456	0.583	0.527	0.581
	Local Strain	4.01	4.27	4.23	0.88	4.54	0.921
	Chemscore	-0.66	1.49	0.839	4.83	6.52	5.00
	PLP	-37.8	-34.6	-34.5	-34.78	-26.69	-32.9
4	RSCC	**0.700**	0.602	0.685	0.586	0.574	0.582
	Local Strain	**-5.42**	5.00	0.383	-5.11	4.21	0.536
	Chemscore	**-5.44**	-4.79	-4.47	2.39	2.23	-1.70
	PLP	**-54.9**	-50.9	-52.1	-44.86	-36.1	-43.9
5	RSCC	0.485	0.524	0.521	0.565	0.537	0.579
	Local Strain	5.24	5.34	5.26	0.563	4.60	0.632
	Chemscore	-1.88	-5.68	-0.613	3.84	7.33	3.96
	PLP	-44.5	-43.7	-43.8	-41.19	-31.96	-40.6
6	RSCC	0.525	0.586	0.586	0.589	**0.591**	0.587
	Local Strain	5.56	5.97	5.96	0.564	**-5.68**	0.516
	Chemscore	-3.97	-4.13	-4.10	0.325	**0.969**	0.713
	PLP	-51.86	-53.1	-53.1	-45.04	**-43.75**	-44.0
7	RSCC	0.532	0.610	0.595	0.621	0.593	0.604
	Local Strain	5.10	-0.199	-42.10	-3.68	4.56	0.616
	Chemscore	-3.26	-1.01	-1.38	1.47	5.90	1.57
	PLP	-43.6	-41.7	-42.1	-37.21	-28.9	-36.5
8	RSCC	0.525	0.552	0.548	0.522	0.515	0.524
	Local Strain	5.61	5.70	5.72	0.53	-3.97	0.464
	Chemscore	-5.64	-3.34	-3.37	2.08	2.08	1.91
	PLP	-49.83	-49.1	-49.1	-41.16	-39.45	-39.7

Note: Fit to FEM map at end of refinement. Variation in strain sometimes may be due to diimethyl morpholino-ring.

SUPPLEMENTARY DISCUSSION

A 'swing-doors mechanism' for DNA gyrase – in which movement of the transport-DNA regulates formation and religation of the double-stranded DNA break in the gate-DNA.

Type IIA topoisomerases (topo2As) are essential enzymes that regulate DNA topology by: creating a double-stranded break in one DNA segment (the gate or G-DNA), then passing a DNA duplex (the transport or T-segment) through this break, before religating the break (Fig. 1). In bacteria, the creation of a temporary double-stranded DNA break is essential for the function of DNA gyrase but it also poses a risk to the cell. If the two 'halves' of the enzyme become separated while the DNA is doubly cleaved, the genomic integrity and viability of the cell may be lost. The 'pair of swing-doors mechanism' that we propose for DNA gyrase (Supplementary Fig. 11) suggests when doubly cleaved DNA is bound across the DNA gate, the gate will tend to automatically swing closed and will tend to remain closed until the T-DNA is pushed through [6], and will then swing closed immediately after the passage of the T-DNA. To the best of our knowledge, crystal structures with an open DNA gate have only been observed for 'apo' topo2A structures which have no DNA bound at the DNA-gate [7-9]. Our 'swing-doors' mechanism is consistent with election microscopy studies of DNA gyrase with doubly-cleaved DNA that did not show any particles with the DNA gate open [10], with SAX studies of DNA gyrase [11] and with other X-ray crystal structures [12, 13]. Our 'swing-doors mechanism' is distinct from a previously proposed 'mechanism for coordinating inter-subunit interactions with DNA cleavage' [14].

Two alternative, but quite similar, mechanisms for metal catalysed DNA-cleavage and religation by topo2As have been proposed: a single moving metal mechanism [1, 15] and a two metal mechanism [16]. In the two metal mechanism it is proposed that both metal binding sites (sites A and B) on the TOPRIM domain can be occupied at the same time, while in the single moving metal mechanism it is proposed that the metal ion moves between the B (also called Y) site and the catalytic A (also called 3') site and cannot occupy both sites at the same time. In both the two metal and single moving mechanisms a metal (Mg^{2+}) ion is required at the A site for metal catalysed DNA-cleavage or religation (see supplementary Fig. 8). The 'swing-doors mechanism' described here requires a metal at the A site for metal catalysed DNA-cleavage or religation (Supplementary Fig. 11), but is compatible with either the two metal or single moving metal mechanisms. A fuller description of the 'swing-doors mechanism' is given below.

In our scheme in Supplementary Fig. 11, prior to the initial cleavage step, the catalytic TOPRIM and WHD domains are 'shown schematically' in a configuration similar to that observed in the apo

S.aureus DNA gyrase structure [1]. The binding of the DNA changes the relative orientations of the TOPRIM and WHD domains within a gyrase^{CORE} subunit, but once the DNA is bound the relative positions of the TOPRIM and WHD domains in a *S.aureus* gyrase subunit remain fixed (to a good approximation) through the rest of the catalytic cycle. In the first DNA cleavage step (Supplementary Fig. 11) the catalytic domains move through the CRsym conformation, to cleave the first DNA strand. DNA cleavage can only occur when the two 'half active sites', from opposite subunits, are aligned across the dimer interface and a catalytic metal (Mg^{2+}) ion is bound at the A site.

Once one DNA strand has been cleaved the stretched gate-DNA [1] is proposed (in the absence of a captured T-DNA segment) to relax, moving the enzyme [17] to a slightly asymmetric Int. (intermediate) conformation to immediately religate the just cleaved first strand. However, if the ATP gate has captured a T-DNA segment, the T-DNA segment will push the transducer domains of the ATPase domains apart [6] and following cleavage of the first strand, will push the DNA-gate into the 'half-open' Casym conformation where the second strand can be cleaved. Furthermore, in the presence of a captured T-DNA segment, the ATPase gate will then push the T-DNA through the DNA-gate temporarily opening 'swing doors' (Supplementary Fig. 11). Once the transport DNA has passed through the DNA gate, the 'swing doors' will swing close and adopt the CRsym conformation to religate the first DNA strand. In the mechanism shown in Supplementary Fig. 11, it is proposed that when the transport T-DNA is sitting between the DNA-gate and the exit gate, the Greek key domain moves the YKGLG motif to 'switch off' the DNA cleavage mechanism, by preventing the catalytic metal moving to the A site. However, the mechanism proposes that at this stage of the catalytic cycle the CRsym conformation religates the doubly cleaved-DNA using a catalytic lysine residue; as discussed below.

Consistent with our proposed mechanism, a recent paper suggests that DNA gyrase can accomplish the first DNA religation step in the absence of metal ions [18] - since incubating quinolone cleavage complexes with EDTA produced singly cleaved DNA. In addition type 1A topoisomerases, which cleave a single DNA strand to modify DNA topology and have a TOPRIM domain and a catalytic tyrosine on a WHD at an active site that resembles that of type 2A topoisomerases [1], can use a lysine residue in catalysis [19]. While the type IA catalytic lysine is not present in topo2As, in our 3.5 Å GSK299423 structure [1] a lysine residue was observed pointing at the scissile phosphate (Supplementary Fig. 12). This lysine residue (residue 581 in *S. aureus* DNA gyrase) is from a topo2A conserved sequence motif, Y<u>K</u>GLG (Supplementary Fig. 5) that is just C-terminal to the small mobile Greek key domain. We suggest that *S. aureus* DNA gyrase may be able to use this lysine residue to catalyse the

first DNA religation step. Mutation of the equivalent lysine in yeast Topo II (Lys 603 – Supplementary Fig. 13) gives a mutant which is capable of cleaving DNA but is defective in DNA religation [20]. The position of the Greek key domain (which is deleted in many of our structures) suggests that it may function to help prevent the exit gate from opening while both strands of the gate DNA are cleaved. If lysine 581 can indeed catalyse the first DNA religation step (but not DNA cleavage), positioning this lysine and reordering the YKGLG motif to prevent a divalent metal from occupying the catalytic A site could ensure that the gate DNA cannot be re-cleaved while the exit gate is open.

The YKGLG motif also adopts different conformations in different crystal structures of eukaryotic topo2As (Supplementary Fig. 13), suggesting that this motif may play a similar role in eukaryotic topo2As. However, the Casym conformation has not been observed in eucaryotic topo2A crystal structures, and the dropped trap-door WHD conformation [16] has not been observed in bacterial topo2A structures. Structural and sequence data (Supplementary Fig. 5) suggest that there are both similarities and differences in the way that bacterial and eucaryotic topo2As move their DNA gates during the catalytic cycle. Although the proposed 'swing doors mechanism' is still somewhat speculative – it does suggest further experiments that can be done to help both differentiate and develop emerging new classes of antibacterial topo2A poisons.

During the course of making revisions to this paper there have been several new publications on QPT-1 and related compounds [21-25], reflecting the growing interest in the new QPT-1 like class of topoisomerase antibacterials.

SUPPLEMENTARY METHODS

QPT-1 synthesis. A scheme for the synthesis of QPT-1[26] (***(2R,4S,4aS)-2,4-dimethyl-8-nitro-2,4,4a,6-tetrahydro-1H,1'H-spiro[[1,4]oxazino[4,3-a]quinoline-5,5'-pyrimidine]-2',4',6'(3'H)-trione)*** is shown in Supplementary Fig. 16.

Step 1: **synthesis of rel-*2-((2R,6S)-2,6-dimethylmorpholino)-5-nitrobenzaldehyde* (1)**. The mixture of 2-fluoro-5-nitrobenzaldehyde (8.62 g, 51.0 mmol), (2R,6S)-2,6-dimethylmorpholine (6.80 g, 59.0 mmol), and potassium carbonate (8.24 g, 59.6 mmol) in N,N-dimethylformamide (DMF) (51.0 mL) was heated in a 150 ^{o}C bath for 75 min. LC/MS showed no more aldehyde starting material remained. The reaction mixture was cooled to room temperature and diluted with EtOAc. The organic layer was washed with water/brine (1/1, 3x), and brine. The organics were dried over Na_2SO_4, filtered, and concentrated to crude **1** (13.5 g, 100 % yield) which was used in the next step without purification. LCMS: M+1=265.1; ^{1}H NMR (400 MHz, CHLOROFORM-*d*) δ ppm 1.19 - 1.37 (m, 6 H) 2.77 - 2.92 (m, 2 H) 3.32 (d, *J*=11.87 Hz, 2 H) 3.85 - 4.00 (m, 2 H) 7.07 (d, *J*=9.09 Hz, 1 H) 8.32 (dd, *J*=9.09, 2.78 Hz, 1 H) 8.64 (d, *J*=2.78 Hz, 1 H) 10.02 - 10.15 (s, 1 H).

Step 2: **synthesis of rel-*5-(2-((2R,6S)-2,6-dimethylmorpholino)-5-nitrobenzylidene)pyrimidine-2,4,6(1H,3H,5H)-trione* (2)**. A mixture of *rel*-2-[(2R,6S)-2,6-dimethyl-4-morpholinyl]-5-nitrobenzaldehyde (13.48 g, 51 mmol) and 2,4,6(1H,3H,5H)-pyrimidinetrione (6.53 g, 51.0 mmol) in methanol (300 mL) was heated to 80 ^{o}C overnight [note: during this time the heat shut off unintentionally]. An orange solid had precipitated which was collected by filtration and washed with methanol (3x) yielding crude **2** (17 g, 85 % yield). The LC/MS showed indicated high purity, but the ^{1}H NMR spectrum showed several impurities. A 2 g portion of crude **2** was suspended in MeOH/CH_2Cl_2 (2/1) then concentrated onto silica gel. Purified by flash column chromatography (40g silica gel column, 0-20% MeOH in CH_2Cl_2). The product precipitated out in the test tube after sitting overnight. The solid was collected, washed with CH_2Cl_2 (3x) to provide the pure **2** (1.3 g, 6.47 % yield) as orange/red solid. LCMS: M+1=375.2; ^{1}H NMR (400 MHz, DMSO-d_6) δ ppm 1.11 (d, *J*=6.06 Hz, 6 H) 2.69 (t, *J*=11.12 Hz, 2 H) 3.30 (d, *J*=12.38 Hz, 2 H) 3.65 - 3.81 (m, 2 H) 7.15 - 7.25 (m, 1 H) 8.01 (s, 1 H) 8.18 - 8.28 (m, 1 H) 8.70 - 8.81 (m, 1 H) 11.26 (s, 1 H) 11.35 (s, 1 H).

Step 3: **synthesis of rel-*(2R,4S,4aS)-2,4-dimethyl-8-nitro-2,4,4a,6-tetrahydro-1H,1'H-spiro[[1,4]oxazino[4,3-a]quinoline-5,5'-pyrimidine]-2',4',6'(3'H)-trione* (3)**. An orange suspension of **2** (0.9 g, 2.404 mmol) in methanol (80 mL) was heated to reflux overnight after which the reaction mixture was a clear yellow solution. The mixture was cooled to rt and a bright yellow solid precipitated. The solid was collected by filtration to give **3** (0.78 g, 1.979 mmol, 82 % yield) as yellow powder. LCMS and NMR indicated a single diastereomer and ^{1}H NMR indicated some methanol was trapped in the solid. The material was used without further purification. LCMS: M+1=375.1; ^{1}H NMR (400 MHz, DMSO-d_6) δ ppm 0.90 - 0.99 (m, 3 H) 1.16 (dd, *J*=6.06, 1.77 Hz, 3 H) 2.87 (d, *J*=14.91 Hz, 1 H) 2.94 - 3.05 (m, 1 H) 3.48 - 3.68 (m, 3 H) 3.92 (dd, *J*=8.97, 1.64 Hz, 1 H) 4.30 (d, *J*=13.39 Hz, 1 H) 6.96 - 7.13 (m, 1 H) 7.83 (s, 1 H) 7.94 - 8.02 (m, 1 H) 11.59 (br. s., 1 H) 11.89 (br. s., 1 H).

Step 4: **Chiral separation to *(2R,4S,4aS)-2,4-dimethyl-8-nitro-2,4,4a,6-tetrahydro-1H,1'H-spiro[[1,4]oxazino[4,3-a]quinoline-5,5'-pyrimidine]-2',4',6'(3'H)-trione* (QPT-1)** and ***(2S,4R,4aR)-2,4-dimethyl-8-nitro-2,4,4a,6-tetrahydro-1H,1'H-spiro[[1,4]oxazino[4,3-a]quinoline-5,5'-pyrimidine]-2',4',6'(3'H)-trione* (*ent*-QPT-1)**. 230 mg of **3** was put on the column with 10 injections (23mg/5mL) (column: chiralpak IA, 5 micron, 21x250mm; mobile phase: heptane/EtOH, 50%B isocratic; flow rate: 20mL/min). The major peaks were collected and coded E1 and E2 respectively (retention times: 9 min & 19 min). It should be noted that there were solubility issues with the sample in EtOH/heptane. 4mL of ETOH and 1 mL heptane was used to dissolve the sample with warming. The sample was then filtered through a 0.25 micron acrodisc and quickly injected onto the column. The sample was warm but not hot upon injection. Each sample had to be prepared just before injection. The final enantiomers were collected and the solvent was evaporated. The yield was lower than expected likely because of the solubility issues. Much remained on the filter and sides of the vials which was recovered as possible (total of 67 mg recovered). The fractions enantiomer 1 and enantiomer 2 were combined appropriately and the solvent was removed under vacuo to give QPT-1 (enantiomer 1: 42 mg, 17% yield, bright yellow solid) and *ent*-QPT-1 (enantiomer 2: 39 mg, 16% yield, bright yellow solid). A reverse phase system was used to evaluate chemical purity and the chiral normal phase system was used to evaluate the enantiomeric purity.

Enantiomeric purity (HPLC): Instrument: HPLC 15 1210 Chiral NP**,** Column: 4.6x150mm**,** Mobile phase: 50/50 Heptane/EtOH no modifier**.** QPT-1: RT=5.029 min, >99.55% ee (42 mg, 17% yield, bright yellow solid). *ent*-QPT-1: RT=11.396 min, =97.2% ee (39 mg, 16% yield, bright yellow solid).

QPT-1: ^{1}H NMR (400 MHz, DMSO-d_6) δ ppm 0.93 (d, *J*=6.57 Hz, 3 H) 1.16 (d, *J*=6.32 Hz, 3 H) 2.86 (d, *J*=14.91 Hz, 1 H) 2.98 (dd, *J*=13.52, 10.74 Hz, 1 H) 3.40 - 3.66 (m, 4 H) 3.91 (d, *J*=8.84 Hz, 1 H) 7.04 (d, *J*=9.60 Hz, 1 H) 7.82 (d, *J*=2.27 Hz, 1 H) 7.97 (dd, *J*=9.22, 2.65 Hz, 1 H).

ent-QPT-1: ^{1}H NMR (400 MHz, DMSO-d_6) δ ppm 0.93 (d, *J*=6.32 Hz, 3 H) 1.16 (d, *J*=6.06 Hz, 3 H) 2.86 (d, *J*=14.91 Hz, 1 H) 2.98 (dd, *J*=13.64, 10.86 Hz, 1 H) 3.44 (dd, *J*=6.95, 3.92 Hz, 1 H) 3.48 - 3.66 (m, 3 H) 3.91 (d, *J*=8.84 Hz, 1 H) 7.03 (d, *J*=9.35 Hz, 1 H) 7.82 (d, *J*=2.27 Hz, 1 H) 7.97 (dd, *J*=9.22, 2.65 Hz, 1 H).

Enantiomer 1, henceforth called QPT-1 is the bioactive (-) enantiomer of QPT-1 used in all studies unless stated. *ent*-QPT-1 is the inactive (+) enantiomer.

SUPPLEMENTARY REFERENCES

1. Bax,B.D. *et al.* Type IIA topoisomerase inhibition by a new class of antibacterial agents. *Nature* **466**, 935-940 (2010).

2. Srikannathasan,V. *et al.* Crystallization and preliminary X-ray crystallographic analysis of covalent DNA cleavage complexes of Staphyloccocus Aureus DNA Gyrase with QPT-1, Moxilfloxacin and Etoposide. *Acta Crystallogr. Sect. F Struct. Biol. Cryst. Commun.* **71**, 1242-1246 (2015).

3. Miller,A.A. *et al.* Discovery and characterization of QPT-1, the progenitor of a new class of bacterial topoisomerase inhibitors. *Antimicrob. Agents Chemother.* **52**, 2806-2812 (2008).

4. Black,M.T. *et al.* Mechanism of action of the antibiotic NXL101, a novel nonfluoroquinolone inhibitor of bacterial type II topoisomerases. *Antimicrob. Agents Chemother.* **52**, 3339-3349 (2008).

5. Lawrence,L.E. *et al.* The inhibition and selectivity of bacterial topoisomerases by BMS-284756 and its analogues. *J. Antimicrob. Chemother.* **48**, 195-201 (2001).

6. Agrawal,A. *et al.* Mycobacterium tuberculosis DNA gyrase ATPase domain structures suggest a dissociative mechanism that explains how ATP hydrolysis is coupled to domain motion. *Biochem. J.* **456**, 263-273 (2013).

7. Fass,D., Bogden,C.E., & Berger,J.M. Quaternary changes in topoisomerase II may direct orthogonal movement of two DNA strands. *Nat. Struct. Biol.* **6**, 322-326 (1999).

8. Berger,J.M., Gamblin,S.J., Harrison,S.C., & Wang,J.C. Structure and mechanism of DNA topoisomerase II. *Nature* **379**, 225-232 (1996).

9. Corbett,K.D., Schoeffler,A.J., Thomsen,N.D., & Berger,J.M. The structural basis for substrate specificity in DNA topoisomerase IV. *J. Mol. Biol.* **351**, 545-561 (2005).

10. Papillon,J. *et al.* Structural insight into negative DNA supercoiling by DNA gyrase, a bacterial type 2A DNA topoisomerase. *Nucleic Acids Res.* **41**, 7815-7827 (2013).

11. Baker,N.M., Weigand,S., Maar-Mathias,S., & Mondragon,A. Solution structures of DNA-bound gyrase. *Nucleic Acids Res.* **39**, 755-766 (2011).

12. Laponogov,I. *et al.* Structure of an 'open' clamp type II topoisomerase-DNA complex provides a mechanism for DNA capture and transport. *Nucleic Acids Res.* **41**, 9911-9923 (2013).

13. Schmidt,B.H., Osheroff,N., & Berger,J.M. Structure of a topoisomerase II-DNA-nucleotide complex reveals a new control mechanism for ATPase activity. *Nat. Struct. Mol. Biol.* **19**, 1147-1154 (2012).

14. Wendorff,T.J., Schmidt,B.H., Heslop,P., Austin,C.A., & Berger,J.M. The structure of DNA-bound human topoisomerase II alpha: conformational mechanisms for coordinating inter-subunit interactions with DNA cleavage. *J. Mol. Biol.* **424**, 109-124 (2012).

15. Laponogov,I. *et al.* Structural basis of gate-DNA breakage and resealing by type II topoisomerases. *PLoS. One.* **5**, e11338 (2010).

16. Schmidt,B.H., Burgin,A.B., Deweese,J.E., Osheroff,N., & Berger,J.M. A novel and unified two-metal mechanism for DNA cleavage by type II and IA topoisomerases. *Nature* **465**, 641-644 (2010).

17. Fogg,J.M. *et al.* Bullied no more: when and how DNA shoves proteins around. *Q. Rev. Biophys.* **45**, 257-299 (2012).

18. Drlica,K. *et al.* Bypassing Fluoroquinolone Resistance with Quinazolinediones: Studies of Drug-Gyrase-DNA Complexes Having Implications for Drug Design. *ACS Chem. Biol.* **9**, 2895-2904 (2014).

19. Schoeffler,A.J. & Berger,J.M. DNA topoisomerases: harnessing and constraining energy to govern chromosome topology. *Q. Rev. Biophys.* **41**, 41-101 (2008).

20. Liu,Q. & Wang,J.C. Similarity in the catalysis of DNA breakage and rejoining by type IA and IIA DNA topoisomerases. *Proc. Natl. Acad. Sci. U. S. A* **96**, 881-886 (1999).

21. Jacoby,G.A., Corcoran,M.A., & Hooper,D.C. The Protective Effect of Qnr on Agents Other than Quinolones that Target DNA Gyrase. *Antimicrob. Agents Chemother.*(2015).

22. Basarab,G.S. *et al.* Responding to the challenge of untreatable gonorrhea: ETX0914, a first-in-class agent with a distinct mechanism-of-action against bacterial Type II topoisomerases. *Sci. Rep.* **5**, 11827 (2015).

23. Basarab,G.S. *et al.* Discovery of Novel DNA Gyrase Inhibiting Spiropyrimidinetriones: Benzisoxazole Fusion with N-Linked Oxazolidinone Substituents Leading to a Clinical Candidate (ETX0914). *J. Med. Chem.* **58**, 6264-6282 (2015).

24. Unemo,M. *et al.* High In Vitro Susceptibility to the Novel Spiropyrimidinetrione ETX0914 (AZD0914) among 873 Contemporary Clinical Neisseria gonorrhoeae Isolates from 21 European Countries from 2012 to 2014. *Antimicrob. Agents Chemother.* **59**, 5220-5225 (2015).

25. Kern,G. *et al.* Inhibition of Neisseria gonorrhoeae Type II Topoisomerases by the Novel Spiropyrimidinetrione AZD0914. *J. Biol. Chem.* **290**, 20984-20994 (2015).

26. Ruble,J.C. *et al.* Synthesis of (-)-PNU-286607 by asymmetric cyclization of alkylidene barbiturates. *J. Am. Chem. Soc.* **131**, 3991-3997 (2009).
